# Structure-Guided Approach for the Development of MUC1-Glycopeptide-Based Cancer Vaccines with Predictable Responses

**DOI:** 10.1021/jacsau.3c00587

**Published:** 2023-11-21

**Authors:** Iris A. Bermejo, Ana Guerreiro, Ander Eguskiza, Nuria Martínez-Sáez, Foivos S. Lazaris, Alicia Asín, Víctor J. Somovilla, Ismael Compañón, Tom K. Raju, Srdan Tadic, Pablo Garrido, Josune García-Sanmartín, Vincenzo Mangini, Ana S. Grosso, Filipa Marcelo, Alberto Avenoza, Jesús H. Busto, Fayna García-Martín, Ramón Hurtado-Guerrero, Jesús M. Peregrina, Gonçalo J. L. Bernardes, Alfredo Martínez, Roberto Fiammengo, Francisco Corzana

**Affiliations:** † Department of Chemistry and Instituto de Investigación en Química de la Universidad de La Rioja (IQUR), Universidad de La Rioja, Logroño 26006, Spain; ‡ Instituto de Medicina Molecular João Lobo Antunes, Faculdade de Medicina, Universidade de Lisboa, Lisboa 1649-028, Portugal; § Department of Biotechnology, University of Verona, Verona 37134, Italy; ∥ Departamento de Tecnología y Química Farmacéuticas, Universidad de Navarra, Pamplona 31008, Spain; ⊥ Angiogenesis Group, Oncology Area, Center for Biomedical Research of La Rioja (CIBIR), Logroño 26006, Spain; # Center for Biomolecular Nanotechnologies@UniLe, Istituto Italiano di Tecnologia (IIT), Arnesano, Lecce 73010, Italy; ∇ Applied Molecular Biosciences Unit UCIBIO, Department of Chemistry, NOVA School of Science and Technology, Caparica 2829-516, Portugal; ○ Associate Laboratory i4HB - Institute for Health and Bioeconomy, NOVA School of Science and Technology, Caparica 2829-516, Portugal; ◆ Institute of Biocomputation and Physics of Complex Systems, University of Zaragoza, Zaragoza 50018, Spain; ¶ Copenhagen Center for Glycomics, Department of Cellular and Molecular Medicine, Faculty of Health Sciences, University of Copenhagen, Copenhagen 2200, Denmark; & Fundación ARAID, Zaragoza 50018, Spain; ● Yusuf Hamied Department of Chemistry, University of Cambridge, Cambridge CB2 1EW, U.K.

**Keywords:** glycopeptides, mucins, antigen, cancer vaccine, gold nanoparticles, NMR, MD simulations, X-ray crystallography

## Abstract

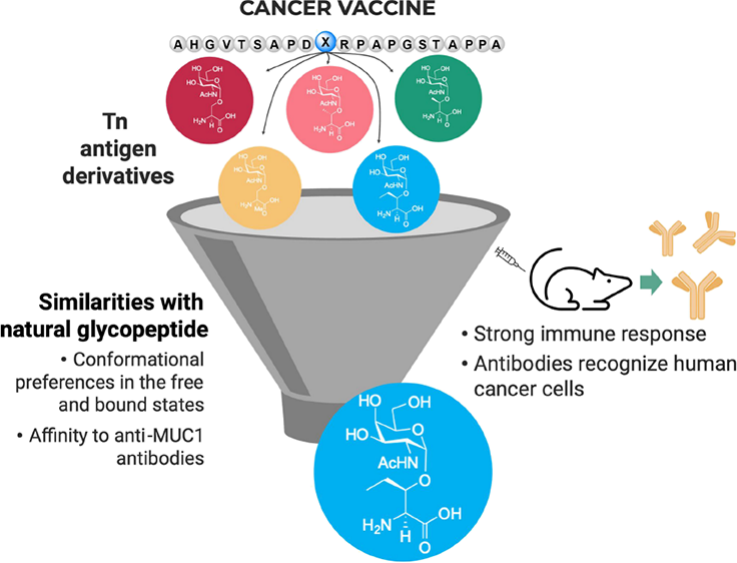

Mucin-1 (MUC1) glycopeptides are exceptional candidates for potential cancer vaccines. However, their autoantigenic nature often results in a weak immune response. To overcome this drawback, we carefully engineered synthetic antigens with precise chemical modifications. To be effective and stimulate an anti-MUC1 response, artificial antigens must mimic the conformational dynamics of natural antigens in solution and have an equivalent or higher binding affinity to anti-MUC1 antibodies than their natural counterparts. As a proof of concept, we have developed a glycopeptide that contains noncanonical amino acid (2*S*,3*R*)-3-hydroxynorvaline. The unnatural antigen fulfills these two properties and effectively mimics the threonine-derived antigen. On the one hand, conformational analysis in water shows that this surrogate explores a landscape similar to that of the natural variant. On the other hand, the presence of an additional methylene group in the side chain of this analog compared to the threonine residue enhances a CH/π interaction in the antigen/antibody complex. Despite an enthalpy–entropy balance, this synthetic glycopeptide has a binding affinity slightly higher than that of its natural counterpart. When conjugated with gold nanoparticles, the vaccine candidate stimulates the formation of specific anti-MUC1 IgG antibodies in mice and shows efficacy comparable to that of the natural derivative. The antibodies also exhibit cross-reactivity to selectively target, for example, human breast cancer cells. This investigation relied on numerous analytical (e.g., NMR spectroscopy and X-ray crystallography) and biophysical techniques and molecular dynamics simulations to characterize the antigen–antibody interactions. This workflow streamlines the synthetic process, saves time, and reduces the need for extensive, animal-intensive immunization procedures. These advances underscore the promise of structure-based rational design in the advance of cancer vaccine development.

## Introduction

Mucin-1 (MUC1) is a highly *O*-glycosylated glycoprotein expressed on the surface of epithelial cells. The extracellular domain of MUC1 comprises tandem repeats of 20 amino acids (AHGVTSAPDTRPAPGSTAPP) that contain five *O*-glycosylation sites.^1,2^ Although this protein displays complex oligosaccharides in healthy tissues, MUC1 expression dramatically increases in tumor cells, which results in their decoration with simple and truncated carbohydrates.^3−5^ This change is mainly the result of mutations in COSMC, a chaperone required for glycosyltransferase C1GalT activity,^6^ and, to a lesser extent, the malfunction or translocation of GalNAc-transferases.^2^ As a result, the most immunogenic epitope (APDTRP), recognized by most anti-MUC1 antibodies,^7−9^ and several tumor-associated carbohydrate antigens (TACAs), such as Tn antigens (α-*O*-GalNAc-Ser/Thr), become exposed and may elicit a weak immune response.^10^ This fact, in combination with the observation that cancer patients can generate anti-MUC1 antibodies in the early stages of disease.^11,12^ has stimulated advancements in MUC1-based vaccine development.

MUC1-based vaccine candidates typically contain the full sequence of MUC1 glycosylated at one or multiple positions with Tn or other TACAs.^13,14^ Despite the synthetic efforts,^15−20^ there have been no successful clinical applications to date,^21^ which may be a result of the atypical glycosylated proteins—that express some of these antigens—being exposed on healthy cells at low concentrations, which may lead to tolerance induction and, consequently, poor immune response in mice.^22^ Our research group and others have been working to overcome this issue by using artificial MUC1 derivatives designed in the lab.^23−25^ These synthetic glycopeptides promise to be more immunogenic and consequently stimulate an effective anti-MUC1 response, as well as being resistant to enzymatic degradation.^26^ It is crucial that such unnatural derivatives feature only subtle structural modifications, so they maintain or even improve binding affinity toward anti-MUC1 antibodies relative to their natural counterparts. In such scenarios, the antibodies generated by these synthetic vaccine candidates will exhibit the ability to identify irregular, naturally occurring MUC1 glycoproteins expressed within tumor cells,^27−29^ known as cross-reactivity.

**Figure 1:**
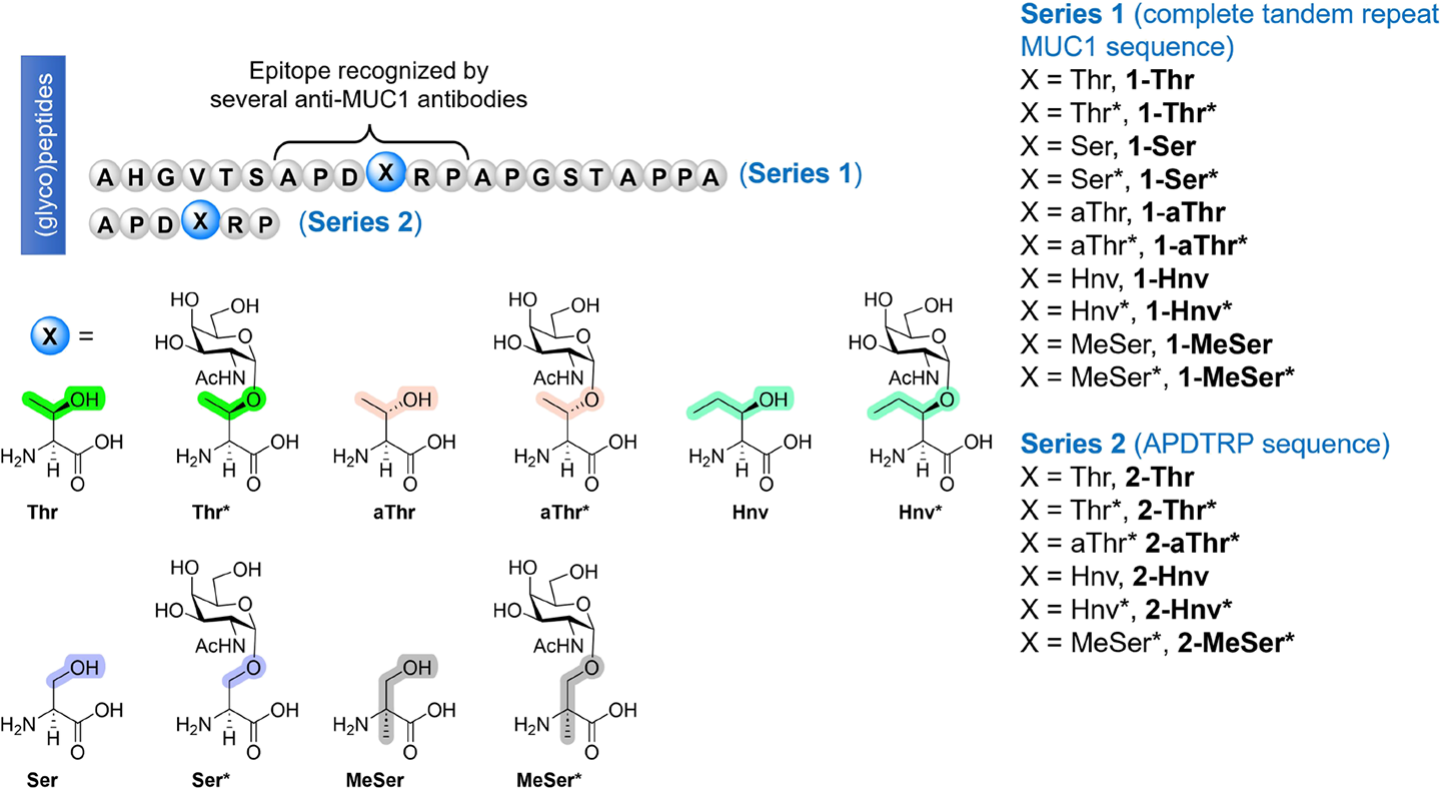
MUC1-like (glyco)peptides synthesized and studied in this work. Series **1** contains the tandem repeat domain of MUC1. Series **2** comprises the most immunogenic region of MUC1 recognized by multiple anti-MUC1 antibodies.^7^

The rational design of unnatural MUC1 antigen derivatives demands a clear understanding of the molecular basis of the antigen–antibody recognition. Here, we propose to use the anti-MUC1 antibody SM3 as a model. We have recently reported the crystal structure of scFv-SM3 in complex with the epitope APDT*RP (in which T* denotes the Tn antigen with Thr).^30^ The structure reveals that the sugar moiety has hydrophobic contact through the *N*-acetyl group with the antibody and a hydrogen bond that involves its primary hydroxyl group. These additional sugar interactions with the antibody may explain the enhanced affinity observed for glycosylated MUC1 derivatives.^7^ The X-ray structure reveals that the methyl group of Thr engages in a weak hydrophobic patch with a tyrosine residue (Tyr32L). The lack of this interaction (i.e., replacement of Thr by a Ser residue) leads to a decrease in affinity to anti-MUC1 SM3 antibody.^30^ Here, we study how MUC1 (glyco)peptides, in which the Thr at the APDTRP fragment is replaced by a noncanonical amino acid with a particular substituent at Cα or Cβ (Figure 1), influence the binding to this anti-MUC1 antibody. This approach allows us to design new cancer antigens with structure-guided approaches that could contribute to the development of valuable vaccines.

The best candidate, in terms of binding affinity toward the SM3 antibody, underwent exhaustive conformational analysis, both in the free state in water and bound to the antibody, to determine the origin of its affinity. We also show that one of these synthetic antigens closely resembles the natural variant in terms of its presentation, behavior, and stability in serum. This observation motivated us to conjugate the antigen to gold nanoparticle carriers with onward experiments in mice to evaluate its efficacy to elicit a humoral immune response. This multidisciplinary strategy combines the synthesis of glycosylated MUC1 derivatives with unnatural amino acids in their sequence, biophysical techniques, X-ray crystallography, nuclear magnetic resonance (NMR) experiments, molecular dynamics (MD) simulations, and in vivo testing.

## Results and Discussion

### Synthesis of Unnatural MUC1-like (Glyco)peptides

As a first step, we synthesized the MUC1 variants shown in Figure 1. The natural threonine present in compounds **1-Thr** and 1-Thr* (the asterisk indicates glycosylation of the residue with α-*O*-GalNAc) was replaced by a serine residue in derivatives **1-Ser** and 1-Ser*. Compounds **1-aThr** and 1-aThr* carry the epimer of threonine at Cβ (allo-threonine). In compounds **1-Hnv** and 1-Hnv*, (2*S*,3*R*)-3-hydroxynorvaline residue, which has an ethyl group at Cβ, is present. Finally, derivatives **1-MeSer** and 1-MeSer* incorporate the unnatural α-methylserine residue to study the effect of a methyl group at Cα on binding to anti-MUC1 antibodies.

**Scheme 1:**
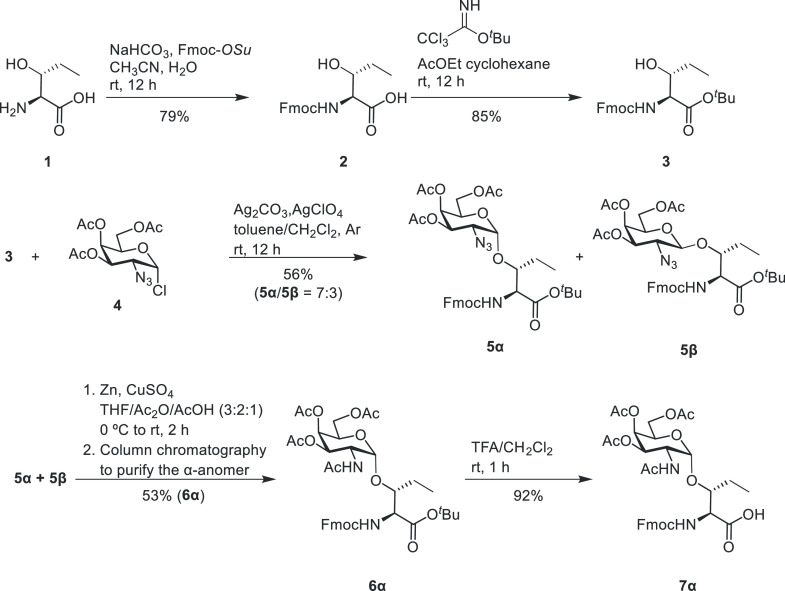
Synthetic Route Followed for the Preparation of Building Block **7α**, Starting from the Commercially Available Amino Acid **1** Fmoc-O*Su* = N-(9H-fluoren-9-ylmethoxycarbonyloxy)succinimide, Fmoc = fluorenylmethoxycarbonyl, ^*t*^Bu = *tert*-butyl group, THF = tetrahydrofuran, and TFA = trifluoroacetic acid.

Although the synthesis of glycosyl-α-amino acids derived from Ser, Thr, and MeSer has been reported,^27,31,32^ those corresponding to unnatural aThr and Hnv residues have been prepared as follows. Building block **7**α derived from Hnv (Scheme 1) was synthesized from commercially available (2*S*,3*R*)-3-hydroxynorvaline (**1**), protected with Fmoc, to give derivative **2** in good yield. Compound **2** was then treated with *tert*-butyl 2,2,2-trichloroacetimidate to yield **3**. The treatment of **3** with sugar derivative **4**,^31^ under classical Koenigs-Knorr conditions, led to a mixture of α and β anomers (compounds **5α** and **5β**, respectively, α/β ratio = 7:3) in moderate yield. Compound **6α** was obtained from a one-pot reaction that transformed the azido group into an acetamide, followed by purification via column chromatography to separate the β-anomer. Finally, the removal of the *tert*-butyl group gave derivative **7α**, which was used in a straightforward microwave-assisted solid-phase peptide synthesis (MW-SPPS). Similarly, we synthesized the glycosyl-α-amino acid derived from aThr (compound **13**, see the Supporting Information). The glycopeptides depicted in Figure 1 were synthesized by using standard MW-SPPS procedures (see the Supporting Information for full details).^29^

### Affinity of Unnatural MUC1-like (Glyco)peptides to the SM3 Antibody

**Figure 2:**
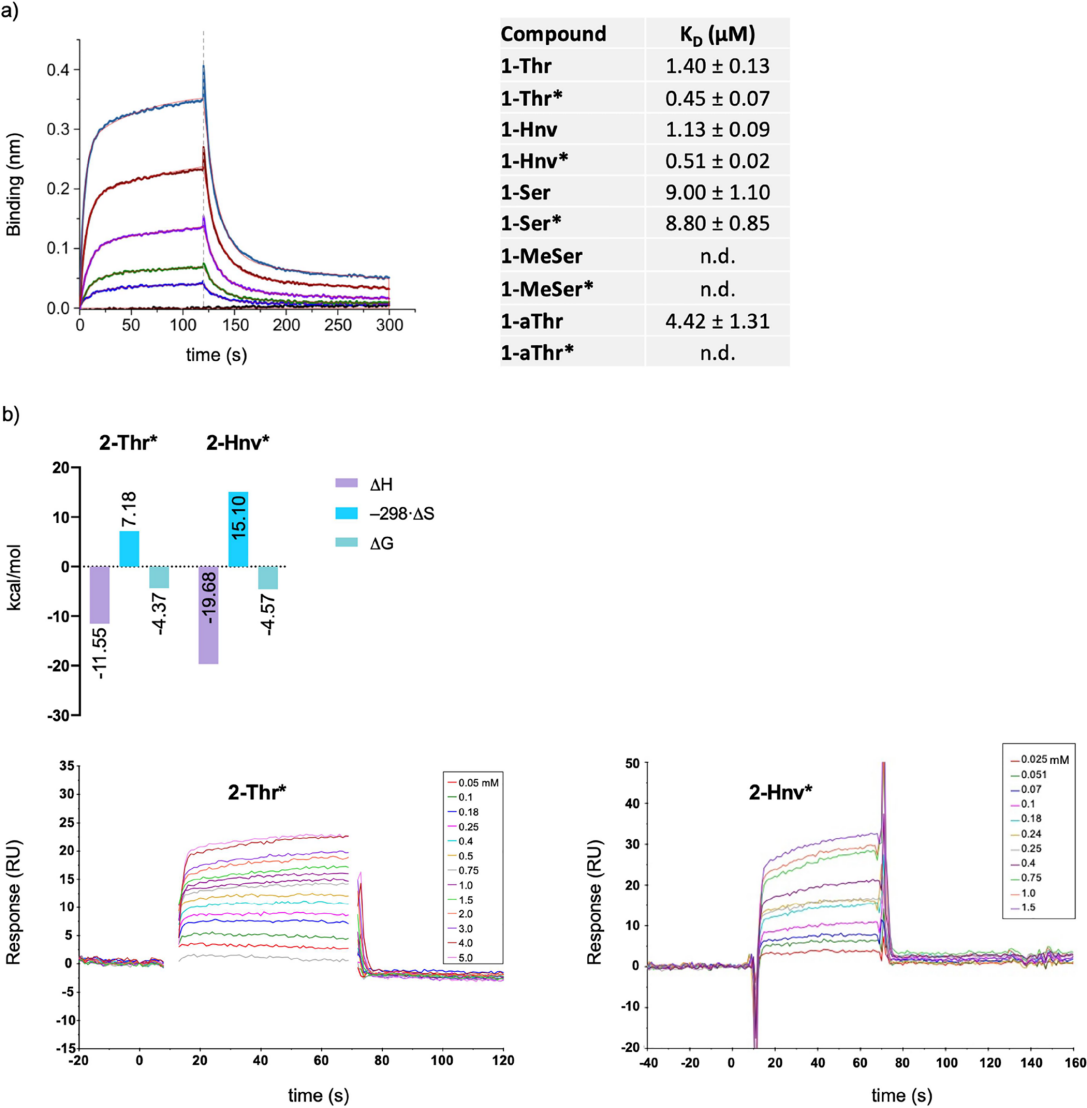
(a) BLI sensorgram obtained for the binding of 1-Hnv* to scFv-SM3 (left). *K*_D_ values for series **1** glycopeptides were measured at 25 °C (right). n.d. stands for not determined affinity, presumably due to very low binding affinity. The *K*_D_ values (±SD) of **1-Thr**, 1-Thr*, **1-Ser,** and 1-Ser* have been previously reported by us (ref (30). SD = standard deviation. (b) Thermodynamic parameters deduced by SPR for the binding of glycopeptides 2-Thr* and 2-Hnv* to the commercially available SM3 antibody at 25 °C, together with the SPR-sensorgrams for 2-Thr* and 2-Hnv*. RU is response units.

With the glycopeptides in hand, we determined their affinity (dissociation constant, *K*_D_) with a recombinantly expressed single-chain variable fragment of the SM3 antibody (scFv-SM3)^30^ specific for the glycosylated APDT*RP epitope,^8^ the structure of which bound to 2-Thr* we recently described.^30^ Biolayer interferometry (BLI) experiments^30^ conclusively showed that the presence of a methyl group at Cβ in the Thr residue is crucial for molecular recognition of MUC1-like derivatives by this antibody (Figures 2a and S1). These findings align with the results previously obtained through BLI analysis of the threonine-containing derivatives.^30^ The absence of the methyl group at Cβ in Ser derivatives results in a significant drop in affinity, more than 6-fold for **1-Ser** and 19-fold for its glycosylated counterpart, 1-Ser*, whereas MeSer variants completely abrogate binding. Similarly, a loss in affinity is observed when the methyl group presents a (*S*)-configuration at Cβ (**1-aThr** and 1-aThr*). The substitution of Thr by Hnv (**Hnv** and Hnv*) was conceived to enhance the CH/π interaction observed in the X-ray structure of the scFV-SM3:APDT*RP complex between the methyl group of Thr and the residue Tyr32L. The binding studies reveal that the synthetic glycopeptides incorporating Hnv exhibit similar affinities than their natural counterparts (Figure 2a). These findings clearly position 1-Hnv* as a compelling candidate for cancer vaccine development, as described below.

A thermodynamic analysis of the antigen–antibody binding was performed for the best candidates (reduced variants 2-Thr* and 2-Hnv*) with the complete SM3 antibody (Figures 2b, S2 and S3, and Tables S1–S3). X-ray structure analyses of short glycopeptides (series 2) were used to support understanding of the thermodynamic properties (see below). *K*_D_ values obtained at different temperatures for each antigen–antibody complex by surface plasmon resonance (SPR) experiments agreed with values obtained with BLI; however, SPR was preferred for analysis because of its simplicity and the minimal use of materials (Figure 2). The data show that the enthalpy contribution is higher for binding 2-Hnv* relative to 2-Thr*. The X-ray structure of the scFv-SM3: 2-Thr* complex revealed that the sugar interacts through a hydrogen bond and CH/π interactions with the antibody.^30^ The ethyl group in Hnv likely favors this latter interaction, which explains the more negative Δ*H* value compared to the natural derivative. The entropy penalty observed for 2-Thr* is attributed to the reduced conformational freedom of the glycosylated residue in the bound state. In fact, although the GalNAc moiety forces the peptide backbone into an extended conformation in solution,^33−37^ binding to the antibody results in a folded conformation.^30^ This entropic disadvantage is compensated by favorable enthalpic contributions between the sugar moiety and the antibody. In the case of 2-Hnv*, an increase in the enthalpy term is observed, which can be explained, at least in part, by the enhancement of CH/π between the side chain of the Hnv residue and the antibody. However, it is important to note that this result is offset by a significant entropy penalty. Therefore, the slightly increased binding affinity in the surrogate glycopeptide can also be explained by the enthalpy–entropy compensation, as explained below.

To broaden the scope of our research to other anti-MUC1 antibodies, we conducted microarray studies with the anti-MUC1 VU-3C6 antibody,^7^ which has a known preference for glycosylation at the APDTRP motif.^38^ Our findings indicate that both unnatural antigen 1-Hnv* and natural derivative 1-Thr* are recognized by VU-3C6 (Figures S4 and S5). The crystal structure of this antibody has not been reported; thus we derived the fine-epitope mapping of 2-Hnv* in complex with VU-3C6 by saturation transfer difference (STD) NMR experiments^39,40^ following protocols we established.^9,41^ The amino acid residues of the antigen that receives more saturation from VU-3C6 antibody were Hnv and Arg (Figure S6). In particular, the −CH_2_CH_3_ protons of Hnv showed the largest STD response, which indicates that this group is in close contact with the VU-3C6 binding site, and the peptide fragment is key for recognition. On the contrary, the STD-NMR suggests that the GalNAc residue should be more exposed to the solvent. This result is consistent with that previously reported for a variant of the 2-Thr* glycopeptide with the VU-3C6 antibody.^9^ The higher affinity of the glycosylated derivatives compared with the naked peptides (**2-Hnv** versus 2-Hnv* in Figure 5S) could likely be because GalNAc glycosylation forces the peptide fragment to adopt the extended bioactive conformation recognized by the antibody.^42^

To explain the high affinity of unnatural glycopeptide 1-Hnv* for the SM3 antibody at the atomic level and considering that this compound could be an optimal antigen for the formulation of a vaccine candidate against cancer, a thorough conformational analysis of this glycopeptide was performed in solution and bound to the SM3 antibody. For this purpose, the reduced derivative 2-Hnv* was used.

### Conformational Analysis of Glycopeptide 2-Hnv* and 2-aThr* in Water

**Figure 3:**
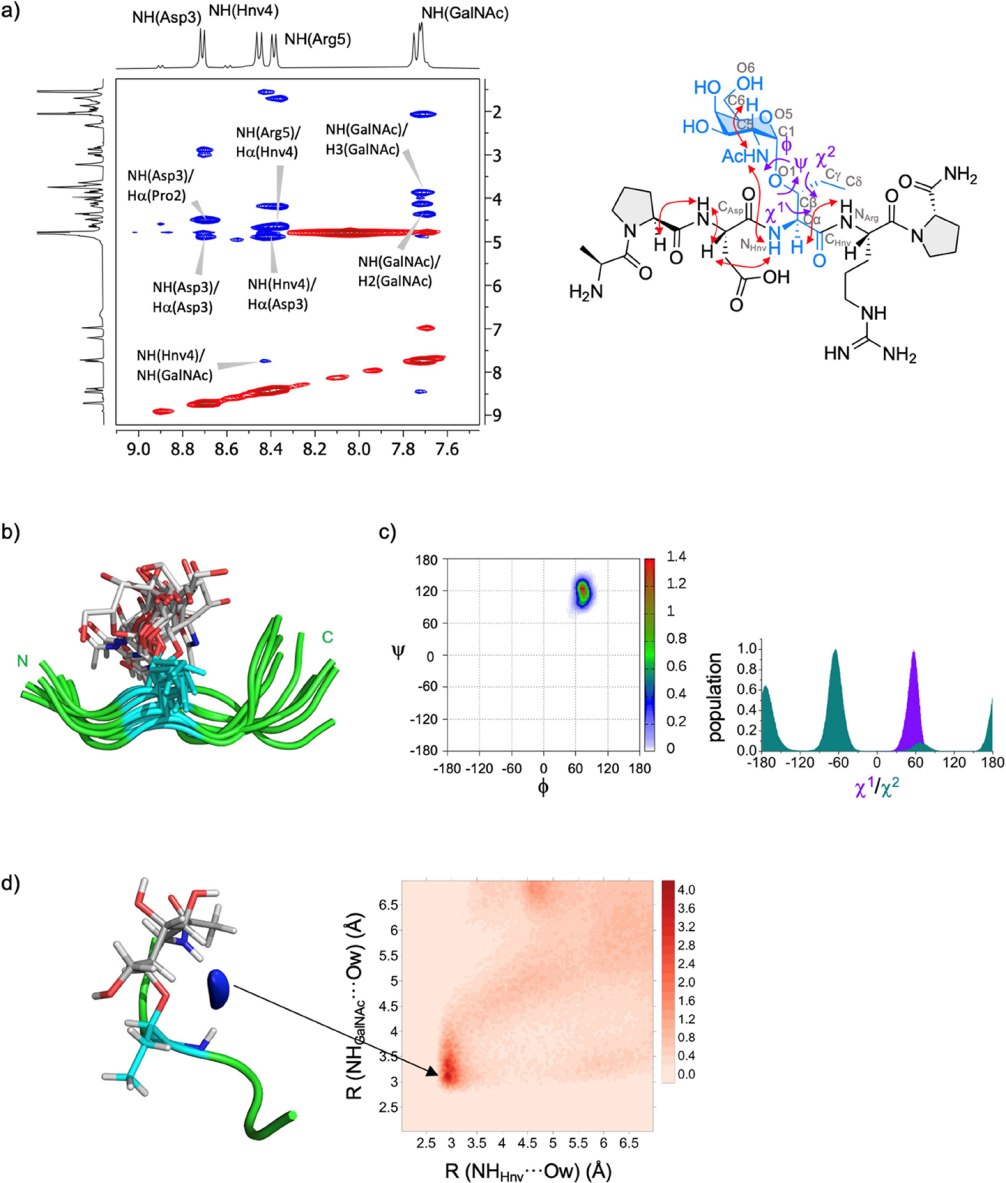
(a) Section of the 500 ms 2D ROESY (400 MHz) spectrum of glycopeptide 2-Hnv* in H_2_O/D_2_O (9:1) at 298 K that shows the amide cross-peak region. Diagonal peaks and exchange cross-peaks connecting NH protons and water are negative (red). The ROE contacts are represented as positive cross-peaks (blue). A second set of signals (in small relative intensity) corresponds to the *cis* configuration of the amide bond of proline residues.^36^ A schematic representation of 2-Hnv* shows the most relevant NOE contacts and the torsional angles. Relevant atoms used for the definition of the following torsional angles are labeled in gray: ϕ_p_ = C_Asp_–N_Hnv_–Cα–C_Hnv_, ψ_p_ = N_Hnv_–Cα–C_Hnv_–N_Arg_, ϕ = O5–C1–O1–Cβ, ψ = C1–O1–Cβ-Cα, χ^1^ = O1–Cβ–Cα–N_Hnv_, χ^2^ = Cα–Cβ–Cγ–Cδ and ω = O5–C5–O6–C6. (b) Structural ensemble derived from structure-guided MD simulations for glycopeptide 2-Hnv*. Peptide, Hnv residue, and the sugar carbon atoms are shown in green, blue, and gray, respectively. (c) Distribution of the glycosidic linkage (ϕ/ψ) and the side chain (χ^1^ and χ^2^) of 2-Hnv* derived from the experiment-guided MD simulations. (d) Representation of the first hydration shell around glycopeptide 2-Hnv* derived from the experiment-guided MD simulations. The 2D radial distribution function^44^ calculated for the nitrogen atoms involved in the bridging water molecule is also shown (NH of GalNAc and NH of the unnatural residue Hnv).

We performed a conformational analysis of unnatural glycopeptide 2-Hnv* in solution by combining 2D-ROESY analysis with MD simulations^34^ (Figures 3, S9, S11, and Table S4). Similarities between glycopeptides 2-Thr*, previously studied by our group,^29^ and 2-Hnv* were evident in the peptide backbone. In particular, the lack of NH-NH ROESY cross-peaks for the peptide, along with the strong-medium peak between Hα(*i*)–NH(*i+1*), hinted at an extended conformation of the peptidic moiety (Figure 3a).^43^ Moreover, a ROESY cross-peak between the NH of the Hnv residue and the NH of GalNAc, distinctive of the eclipsed conformation of the glycosidic linkage in GalNAc-Thr,^33^ suggested similar conformational behavior for the glycosidic linkage as in 2-Thr*.

Key proton–proton distances derived from the 2-Hnv* ROESY spectrum were used as time-averaged restraints^45^ in experiment-guided MD simulations following our well-established protocol.^34^ These restraints were included for the peptide backbone and the glycosidic linkage. The excellent agreement between the experimental and theoretical derived distances and ^3^*J* coupling constants (Table S4) supported our calculations.

The structural ensemble, consistent with the experimental distances, is shown in Figure 3b, where an extended conformation of the peptide backbone around the glycosylation point is shown. This result was partially supported by the findings of the circular dichroism experiments, which showed a similar conformational behavior for both 2-Thr* and 2-Hnv* (Figure S8). These experiments revealed a negative band at approximately 195 nm and very low ellipticity readings above 220 nm.^46^ According to the simulations, the glycosidic linkage of 2-Hnv* displayed a conformation centered at ϕ/ψ ≈ 65°/120°, which agrees with the *exo*-anomeric effect,^47^ and with the typical eclipsed arrangement conformation observed for 2-Thr* (ϕ/ψ ≈ 65°/120°, Figure 3c).^33^ The side chain of the unnatural residue in 2-Hnv* was rigid in solution, with a conformer characterized by χ^1^ = 60°. On the contrary, χ^2^ was somewhat flexible, with two main conformers (χ^2^ = 180° or −60°, Figure 3c). Interestingly, 2-Hnv* in solution also held bridging-water molecules between the peptide backbone and the GalNAc, as previously observed for the natural variant (Figure 3d).^33^ This interaction could be responsible for the extended conformation of the backbone. According to the MD simulations, the hydroxymethyl group of the GalNAc, which is characterized by the ω torsional angle (Figure 2a), exhibits a rotamer population of 46:54:0 (*gt*/*tg*/*gg*), indicating that this dihedral is flexible in solution. This distribution leads to values for ^3^*J*_H5,H6R_ and ^3^*J*_H5,H6S_ of 5.3 and 5.1 Hz, respectively, which aligns somewhat with the experimental coupling constants ^3^*J*_H5,H6R_ and ^3^*J*_H5,H6S_ of 6.1 Hz and a rotamer distribution of ca. 40:50:10 (*gt*/*tg*/*gg*).^48^ Overall, unnatural glycopeptide 2-Hnv* perfectly mimics the conformational landscape of the naturally occurring glycopeptide in aqueous solution.^29^ A similar approach was used to study glycopeptide 2-aThr* (Figures S7, S10, S11, and Table S4), which shows that it also adopts an extended conformation in water. However, in this case, the glycosidic linkage exhibits an entirely different conformation (Figure S11), which is a crucial difference between the two glycopeptides in terms of Tn antigen presentation. This observation provides a structural explanation for the poor binding of 2-aThr* reported in the previous section.

Finally, for 1-MeSer*, we have already reported that the presence of the unnatural α-methylserine imposes flexibility on the peptide backbone and the glycosidic linkage that results in completely different conformational preferences for this antigen in solution relative to the natural one, which could be responsible for the lower efficacy of the corresponding vaccine candidate.^27^

### Conformational Analysis of Glycopeptide 2-Hnv* Bound to scFv-SM3 Antibody

**Figure 4:**
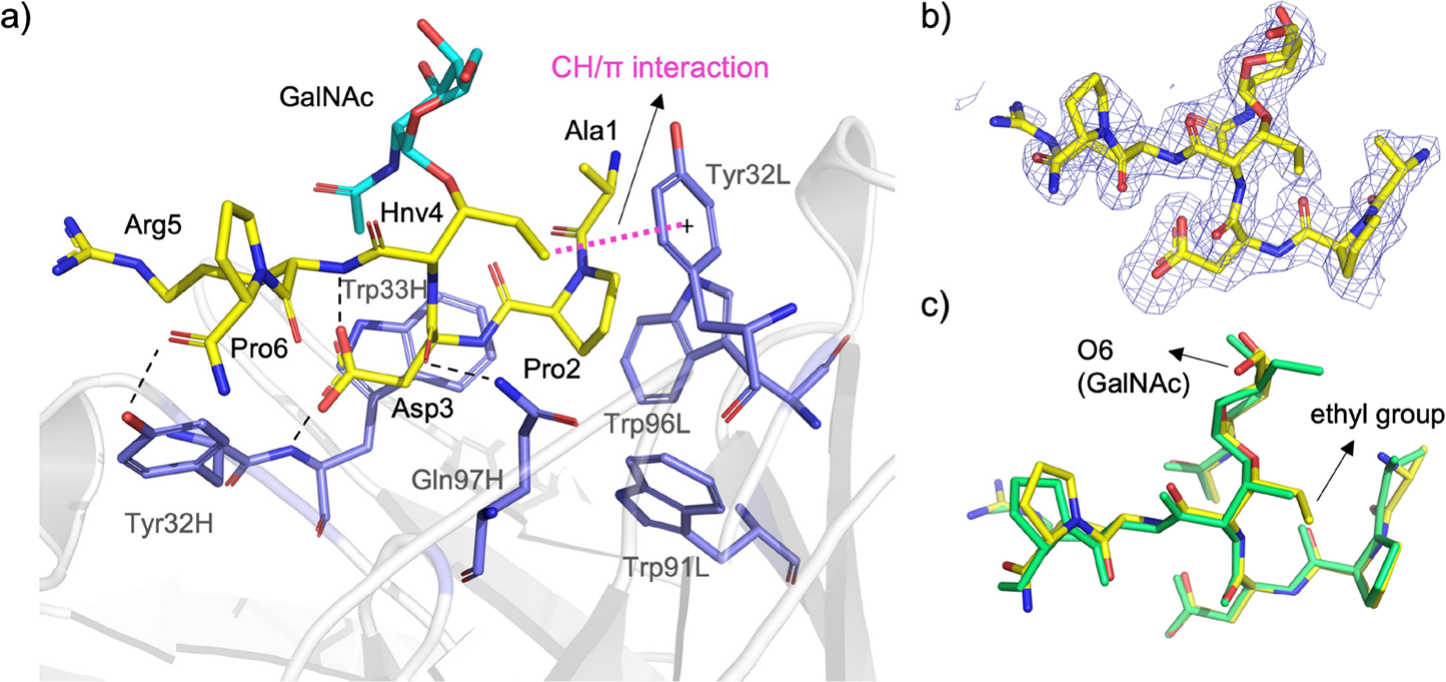
(a) Analysis of the X-ray structures of glycopeptide 2-Hnv* in complex with scFv-SM3 (PDB entry: 8AXH). Pink and black dashed lines indicate CH/π and hydrogen-bonding interactions, respectively. Carbon atoms of the peptide fragment and sugar of 2-Hnv* are in yellow and blue, respectively. The antibody is shown as white ribbons, with the binding site residues shown as sticks with carbon atoms colored purple. (b) Electron density maps are F_O_–F_C_ syntheses (blue) contoured at 2.2 σ for glycopeptide 2-Hnv*. (c) Superposition of glycopeptides 2-Thr* (in green)^30^ and 2-Hnv* (in yellow) in complex with scFv-SM3.

We obtained the crystal structure of the scFv-SM3 antibody^30^ complexed with glycopeptide 2-Hnv* at high resolution (1.85 Å, Figure 4 and Table S5; PDB entry: 8AXH). The conformation of 2-Hnv* in this complex is similar to that adopted by 2-Thr*/scFv-SM3 (Figure 4c).^30^ By analogy with the natural glycopeptide, the Hnv residue adopts a helixlike conformation in the bound state (with ϕ_p_ and ψ_p_ close to −89.0° and 9.2°, respectively; see Figure 3a for torsional angle definition). The glycopeptide is also characterized by a glycosidic linkage and a side chain of ϕ/ψ = 67.4°/80.1° and χ^1^ = 50.3°, respectively (see Figure 3a for torsional angle definition). This conformer allows the ethyl group of Hnv to interact with the aromatic ring of Tyr32L (interaction in pink in Figure 4) more efficiently than the methyl group of Thr in 2-Thr* [distance of ethyl group (Hnv)-Tyr = 4.2 Å vs methyl (Thr)-Tyr = 4.7 Å]. Additional CH/π interactions, such as those between the side chains of Asp and Arg with Trp33H and Tyr32H, respectively, are also observed in this crystal structure. Similarly, Pro2 stacks with Trp91L, Trp96L, and Tyr32L, and the methyl group of Ala1 stacks with Tyr32L. The carbonyl groups of Pro6 and Asp3 are involved in hydrogen bonds with Tyr32L and Gln97H, respectively. The carboxylic acid of the side chain of Asp3 is engaged in hydrogen bonding with the NH group of the main chain of Trp33H and with the NH of Arg5.

Interestingly, in contrast to the complex with natural glycopeptide 2-Thr*, there is no hydrogen bond between the hydroxymethyl group of GalNAc and Tyr32L. Whereas the hydroxymethyl group of GalNAc displays a *tg* conformation in 2-Thr*, it adopts a *gt* conformer in 2-Hnv*, which precludes the formation of hydrogen bonding. The enhanced CH/π stacking between the unnatural residue and the antibody and the entropy penalty associated with the restricted movement of the ethyl group of this residue in the bound state could explain the similar affinity between Thr- and Hnv-containing glycopeptides toward the SM3 antibody. However, it is important to note that the increase in enthalpy of the glycopeptide 2-Hnv* compared to the natural variant (about 8 kcal/mol according to the data shown in Figure 2b) is not exclusively due to a CH/π interaction, as it rarely exceeds 2 kcal/mol.^49^ Also, the rigidification of a single torsional degree of freedom in the ethyl group cannot explain the estimated 8 kcal/mol difference in entropy.^50^ It is likely that other effects, including the role of solvation and desolvation events, are also responsible for the observed binding energy.

**Figure 5:**
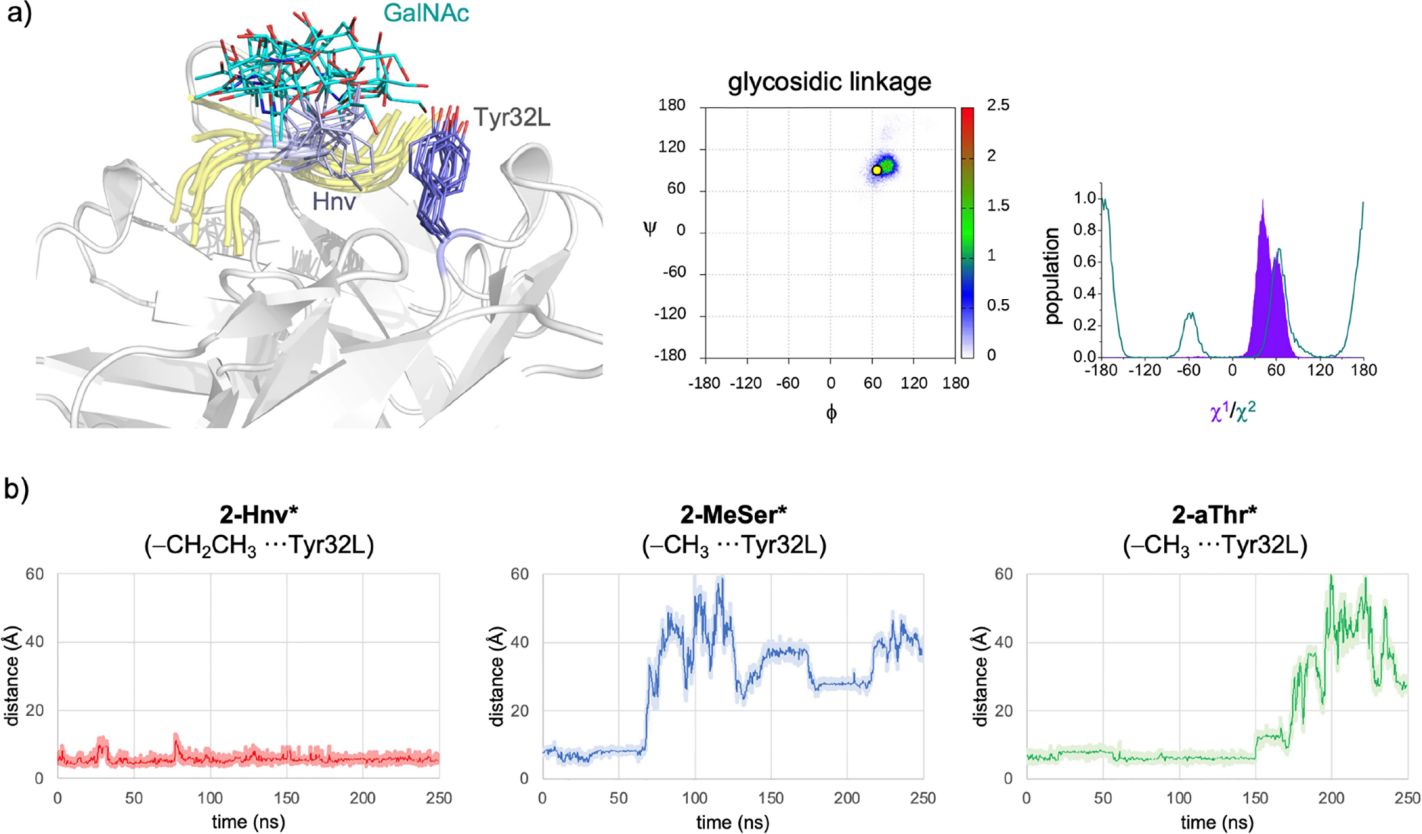
(a) Overlay of 10 frames of the complex between 2-Hnv* and the Fab fragment of SM3 (PDB entry: 1SM3)^51^ sampled from MD simulations. Carbon atoms of the peptide fragment and sugar of 2-Hnv* are in yellow and blue, respectively. The unnatural Hnv residue is colored light purple. The antibody is shown as white ribbons, with Tyr32L shown as sticks with purple carbon atoms in purple. The population of the glycosidic linkage, side chain (χ^1^), and χ^2^ is also shown. The yellow dot denotes the conformation of the glycosidic linkage found in the crystallographic structure reported in this work. ϕ = O5–C1–O1–Cβ, ψ = C1–O1–Cβ–Cα, χ^1^ = O1–Cβ–Cα–N. χ^2^ = Cα–Cβ–Cγ–Cδ . (b) MD traces of the distances between the carbon of ethyl (−CH_2_CH_3_)/methyl group of the glycosylated amino acid and the center of the aromatic ring of Tyr32L for the complexes between the Fab fragment of SM3 and 2-Hnv* (left panel), 2-MeSer* (middle panel), and 2-aThr* (right panel).

We also performed MD simulations of the complexes of 2-Hnv*, 2-aThr*, and 2-MeSer* with the scFv-SM3 antibody (Figures 5 and S12). Interestingly, only the complex with the glycopeptide that contains the Hnv residue was stable throughout the simulation. The glycopeptides 2-MeSer* and 2-aThr* were released from the complex with the antibody after ≈60 and 150 ns, respectively (Figure 5b). These results support the fact that we were unable to determine the *K*_D_ of these derivatives against the scFV-SM3 antibody by BLI experiments. According to the calculations, the glycosidic linkage of 2-Hnv* shows the same conformation as that in the crystallographic structure (Figure 5a). The CH/π interaction between the ethyl group of the unnatural residue and Tyr32L is retained throughout the simulation time. Although the side chain (represented by χ^1^) is constrained, the ethyl group is quite flexible, as shown in Figure 5a, and has the three possible staggered conformers for χ^2^ torsion angles, which could explain the enthalpy–entropy balance commented above. It is important to mention that according to the MD simulations the hydroxymethyl group of the GalNAc remains flexible in the bound state in solution, with a rotamer population of 61:37:2 (*gt*/*tg*/*gg*). Moreover, as observed in the complex between the SM3 antibody and the natural glycopeptide, the population of hydrogen bonds between the side chain of Tyr32H and the OH6 is about 10%.^30^

### Preparation and In Vivo Studies of an Anticancer Vaccine Candidate Based on Glycopeptide 1-Hnv*

**Figure 6:**
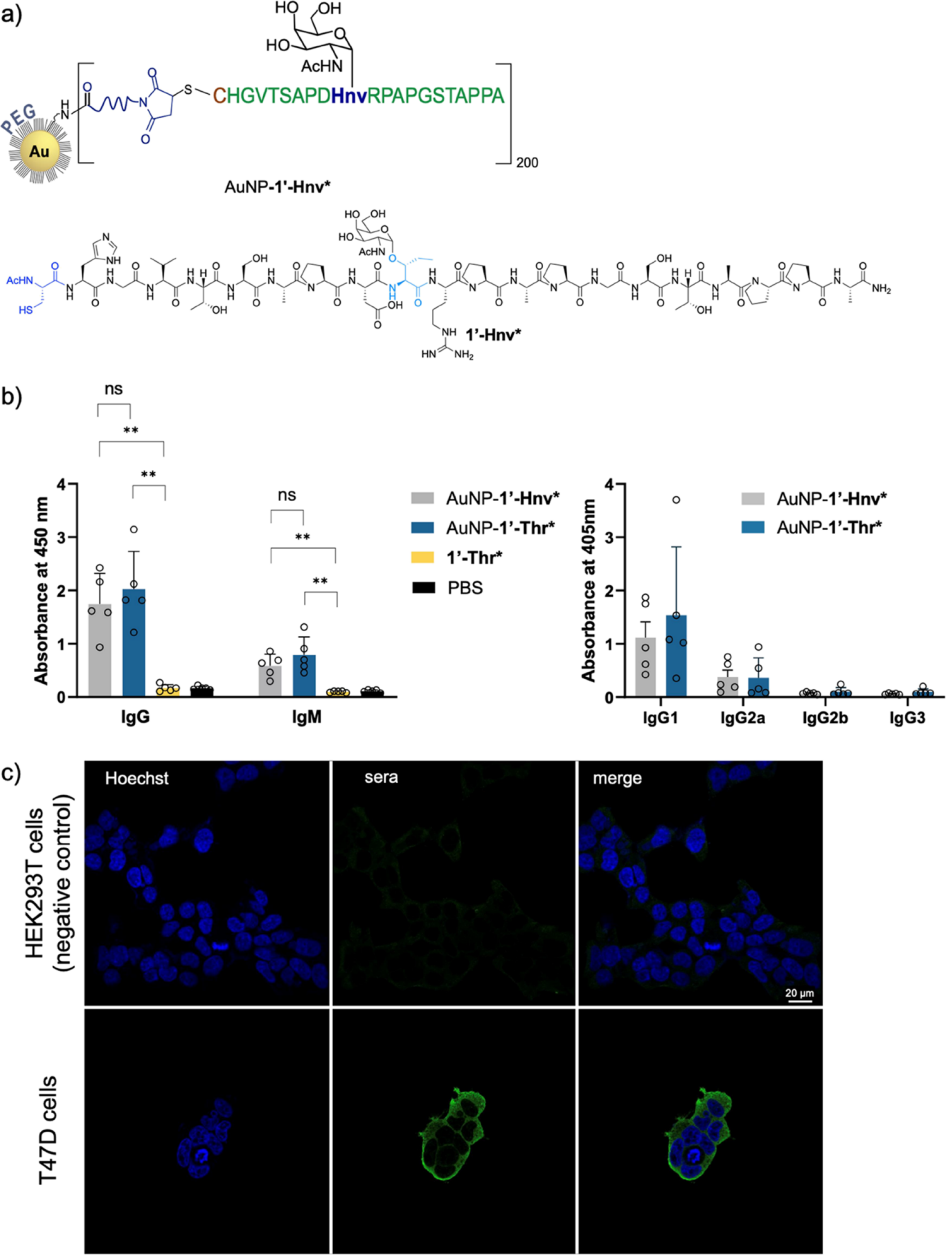
(a) Schematic representation of the vaccine candidate containing glycopeptide 1′-Hnv* attached to the surface of gold nanoparticles (AuNP-1′-Hnv*). (b) Left panel: Total anti-MUC1 antibodies (IgG and IgM) after immunizing BALB/c mice (*n* = 5 per experimental group) with AuNP-1′-Thr* and AuNP-1′-Hnv*. Glycopeptide 1′-Thr* and PBS were used as a control groups (1:200 dilution). Right panel: Total IgG subtyping (IgG1, IgG2a/b, and IgG3) anti-MUC1 antibodies after immunizing mice (*n* = 5 per experimental group) with AuNP-1′-Thr* and AuNP-1′-Hnv*. ELISA plates were coated with glycopeptide 1′-Hnv* conjugated to bovine serum albumin (BSA-1′-Hnv*). Bars represent the mean ± SD of all animals. Asterisks indicate statistically significant differences (**p < 0.05), and “ns” indicates no significant difference. No significant difference in antibody subtypes was observed between the two vaccine candidates. Comparisons were performed using unpaired *t* test with Welch’s correction (Prism 9.5.1). (c) Confocal microscopy images show that mice antibodies elicited in mice with AuNP-1′-Hnv* stain MUC1-expressing T47D cells but not HEK293T cells. Blue = Hoechst (nuclei); green = secondary antimouse IgG Alexa 488. Bar size = 20 μm.

After demonstrating that glycopeptides that contain the Hnv residue (1-Hnv* and 2-Hnv*) exhibit a conformational landscape very similar to that of natural variants 1-Thr* and 2-Thr*, we explored the potential of this antigen mimic in the formulation of a cancer vaccine candidate. The *N*-terminal Ala residue present in 1-Hnv* was substituted by a Cys to give derivative 1′-Hnv*, to allow for conjugation to maleimide-functionalized PEGylated gold nanoparticles (AuNPs, vaccine candidate AuNP-1′-Hnv*, Figures 6a and S13) prepared according to our reported protocol.^28^ The conjugation reaction was confirmed by gel electrophoresis and dynamic light scattering analyses (Figure S13).

The number of antigen copies per AuNP was estimated by amino acid analysis, resulting in a loading of approximately 200 glycopeptides/AuNP.^28^ The same strategy was followed also for the natural antigen affording AuNP-1′-Thr* (Figure S13). The physicochemical characterization of AuNP-1′-Hnv* and AuNP-1′-Thr* is shown in Table S6. Interestingly, the apparent *K*_D_ of the SM3 antibody to these derivatives is consistent with those obtained in BLI experiments (Figures 2a and S14).

These vaccine candidates were tested in mice according to the immunization protocol described in the Methods section and in the ESI. Groups of five BALB/c mice were immunized with a prime dose followed by three equal booster doses of AuNP-1′-Thr* or AuNP-1′-Hnv* (each dose equivalent to 2 μg of the glycopeptide) at 21-day intervals. Glycopeptide 1′-Thr* and phosphate-buffered saline (PBS) were administered as control groups. (Figure S15). We decided to use the nanoparticles without additional adjuvant to investigate the intrinsic adjuvanticity of the nanoparticle-based formulation.

Mice were sacrificed 5 days after the last booster dose, and sera were collected. Notably, the analysis of the sera revealed that both vaccine candidates (AuNP-1′-Hnv* and AuNP-1′-Thr*) elicited anti-MUC1 IgG antibodies (Figures 6b and S16), and there was no significant difference observed between them. The data also shed light on a T cell-mediated class switching process, as shown by the low IgM antibody levels observed with both vaccine candidates. (Figure 6b).^13^ Antibody isotype analysis showed that IgG1 was the predominant antibody in all mice (Figures 6b and S16), indicating that the vaccine candidate elicited predominantly Th2-type immune responses. IgG2a, IgG2b, and carbohydrate-related IgG3 antibodies^52^ were detected at very low concentrations in all animals, which is consistent with the expected maturation of the immune response after immunization.^53^

Cross-reactivity was also observed for antibodies elicited with both formulations, that is, antibodies elicited by the non-natural antigen mimic bind to the natural antigen (compare Figures 6b and S16). Next, we evaluated the ability of the antibodies elicited with the unnatural antigen to recognize tumor-associated MUC1 on the surface of human breast cancer cell lines. For this purpose, the MUC1-expressing T47D cell line^54^ was treated with sera from mice immunized with the AuNP-1′-Hnv*, and the MUC1-lacking HEK293T cell line was used as a negative control. Confocal microscopy images (Figure 6c) clearly confirm that the antibodies elicited with AuNP-1′-Hnv* selectively recognize breast cancer cells. This result demonstrates the potential of cancer vaccines that contain unnatural residues.

The immunization study is consistent with the similar conformational analysis of glycopeptides 1′-Thr* and 1′-Hnv* and their affinity for antibody SM3. Remarkably, we observed a similar trend when we compared our newly developed vaccine candidate containing a MUC1 glycopeptide (designated Thr*) with a variant containing the unnatural MeSer residue (designated MeSer*).^27^ In our current study, we demonstrated that the affinity of antibody SM3 for 1′-MeSer* was significantly lower relative to the parent peptide. This result is consistent with our in vivo studies, in which a significantly lower number of antibodies were produced in mice compared to the natural peptide. These results suggest a possible correlation between antigen presentation, binding affinity (*K*_D_) of the SM3 antibody, and the immune response elicited in mice. Interestingly, this correlation holds true regardless of the antigen delivery system used, as observed both in our current study using AuNPs and in a previous study that used liposomes with the MeSer* derivative.^27^

## Conclusions

We have developed a new cancer vaccine candidate based on an MUC1-derived GalNAc glycopeptide by replacing the threonine residue at the immunodominant epitope with (2*S*,3*R*)-3-hydroxynorvaline (Hnv). This synthetic surrogate exhibits very similar conformational behavior in solution and when bound to an antitumor antibody relative to the natural variant. Enthalpy–entropy compensation explains the similar affinity of the unnatural antigen to the SM3 anti-MUC1 antibody compared to the original derivative. The potential of this modified glycopeptide as a mimic of a tumor-associated MUC1 antigen triggered us to test it in vivo, which proved that antibodies generated in mice with this synthetic antigen recognized human cancer cell lines with high selectivity.

To date, we have developed four vaccine candidates based on unnatural MUC1 derivatives in different formulations (liposomes, AuNPs, or carrier protein) and compared all of them with the natural MUC1 variant. The liposome-based vaccine containing α-methylserine, in which the unnatural antigen is highly dynamic and has the lowest affinity for SM3,^27^ elicits a weak immune response relative to a similar vaccine candidate that contains the natural Thr glycopeptide. In contrast, the thio-threonine-based vaccine, in which the antigen is conjugated to AuNPs and whose glycopeptide antigen presents a high affinity, produces higher antibody titers than the natural variant.^28^ A similar scenario is demonstrated with the glycopeptide containing an sp^2^-iminosugar conjugated to a carrier protein.^29^ The unnatural antigen described herein, based on the noncanonical Hnv, exhibits an affinity analogous to that of the natural antigen toward the SM3 antibody and shares a similar conformational profile, which could explain their comparable humoral immune response. It is worth noting that the natural glycopeptide serves as a common element in these different formulations, whether in liposomes, AuNPs, or a carrier protein.

Although we did not perform a correlation study, the artificial antigen must be able to stimulate an effective anti-MUC1 response. To this end, the simple properties of conformational analysis and a reasonable *K*_D_ with antitumor antibodies, such as SM3, have the potential to become effective tools to predict the immunogenic effect of synthetic antigens. This approach has the potential to facilitate the development of more robust vaccines, circumvent unnecessary synthetic complexity, and minimize time-consuming and animal-intensive immunization protocols by simply evaluating these properties.

## Methods

### Solid-Phase Peptide Synthesis

(Glyco)peptides were synthesized by stepwise microwave-assisted solid-phase peptide synthesis on a Liberty Blue synthesizer using the Fmoc strategy on a Rink Amide MBHA resin (0.1 mmol). Fmoc-Thr[GalNAc(Ac)_3_-α-D]–OH was synthesized as described in the literature.^31^ This compound and the glycosylamino acids synthesized in this work (2.0 equiv) were manually coupled using HBTU [(2-(1*H*-benzotriazol-1-yl)-1,1,3,3-tetramethyluronium hexafluorophosphate] (0.9 equiv) and diisopropyl ethyl amine – DIPEA– (2.0 equiv), while all other Fmoc amino acids (5.0 equiv) were automatically coupled using oxyma pure/DIC (*N*,*N*′-diisopropylcarbodiimide). For glycopeptides **1′-Thr** and **1′-Hnv**, the Cys residue was *N*-acetylated by the treatment of the protected glycopeptides attached to the resin with acetic anhydride/pyridine (2:1) at rt for 2 h. The *O*-acetyl groups of GalNAc moiety were removed treating the resin-bound peptide with a mixture of NH_2_NH_2_/MeOH (7:3) 3 × 5 mL. (Glyco)peptides were then released from the resin, along with the removal of the acid-sensitive side chain protecting groups, using TFA 95%, triisopropylsilane (TIS) 2.5%, and H_2_O 2.5% (3 mL) for 3 h at 25 °C or 30 min at 37 °C. For glycopeptides **1′-Thr** and **1′-Hnv**, a solution of TFA/TIS/H_2_O/EDT (92.5:2.5:2.5:2.5, 3 mL) was used for 3 h at 25 °C without microwave irradiation. (Glyco)peptides were then precipitated with cold diethyl ether (20 mL) and centrifuged for 6 min at 6500 rpm. The supernatant solution was discarded, and this process was repeated twice. Finally, (glyco)peptides were dried and redissolved in water to be purified by reverse phase HPLC on a Phenomenex Luna C18(2) column (10 μm, 250 mm × 21.2 mm) with a flow rate of 10 mL/min. UV detection was performed at 212 nm.

The synthesis and characterization of compounds **1-Thr**, 1-Thr*, **2-Ser**, 2-Ser*, **2-Thr,** and 2-Thr* have been previously described.^30^

### Biolayer Interferometry Assays

Binding assays were performed on an Octet Red Instrument (fortéBIO). Ligand immobilization, binding reactions, regeneration, and washes were conducted in wells of black polypropylene 96-well microplates. (Glyco)peptides (10 mg/mL) were immobilized on amine-reactive biosensors (AR2G biosensors) in 10 mM sodium acetate pH 5.5 buffer, using 1-ethyl-3-(3-(dimethylamino)propyl)carbodiimide and *N*-hydroxysuccinimide as a coupling agent for 10 min at 1000 rpm at 25 °C. The excess reactive esters were then blocked with a solution of ethanolamine hydrochloride (1 M, pH 8.5), followed by regeneration (glycine pH 2.0 buffer) and washing. Binding analyses were carried out at 25 °C, 1000 rpm in 10 mM sodium phosphate buffer (pH 7.4) containing 150 mM NaCl, using different concentrations of scFv-SM3 antibody.^30^ The surface was thoroughly washed with the running buffer without a regeneration solution. Data were analyzed using Data Analysis (fortéBIO) with Savitzky-Golay filtering. Binding was fitted to a 2:1 heterogeneous ligand model. Steady-state analysis was performed to obtain the binding constants (*K*_D_, Figure S1).

### Surface Plasmon Resonance Assays

SPR experiments were performed with a Biacore X-100 apparatus (Biacore GE) using 25 mM PBS buffer with 0.005% tween as running buffer at temperatures of 7–35 °C. Flow cells (CM5 sensor chip; Biacore) were activated for 7 min by injecting 140 μL of a 1:1 ratio of aqueous 50 mM *N*-hydroxysuccinimide (NHS):200 mM ethyl-3(3-dimethylamino)propylcarbodiimide (EDC). Commercially available SM3 antibody (from Abcam) was immobilized on the activated gold chip in flow cell 2 by injection of a 100 μg/mL protein solution diluted with 10 mM sodium acetate buffer with a flow rate of 10 μL/min for 7 min, followed by an injection of 130 μL ethanolamine to block any remaining activated groups on the surface. The level of immobilization reached was about 3000 RUs. Flow cell 1, used as a reference, was blocked with ethanolamine under the same conditions as flow cell 2 without immobilization of protein. Affinity experiments were conducted using a series of different concentrations of each epitope in the range of 0.025–5 mM with a flow rate of 30 μL/min for 60 s. Each injection was followed by a 100 s injection of running buffer (dissociation phase). No regeneration steps were performed between injections. Response data were collected in real time and analyzed with the Biacore X-100. Evaluation software and plotted as response shift versus analyte concentration. SPR curves obtained for glycopeptides 2-Thr* and 2-Hnv* at 25 °C are shown in Figure S2. *K*_D_ values assessed at different temperatures are shown in Table S1.

### STD-NMR Studies

The interactions of glycopeptide 2-Hnv* in the presence of the anti-MUC1 antibody VU-3C6 (ref (7) were also studied by STD-NMR using our previous protocol.^9^ All of the NMR experiments were recorded on a Bruker Avance III 600 MHz spectrometer equipped with a 5 mm inverse detection triple-resonance cryogenic probe head with z-gradients. The glycopeptide 2-Hnv* was completely assigned through standard 2D-TOCSY (30 and 80 ms mixing time) and 2D-NOESY (400 ms mixing time) at 278 K. The glycopeptide was characterized in a buffer containing 20 mM PBS, 20 mM NaCl, 0.09% NaN_3_ buffer, pH 7.1 in H_2_O/D_2_O (90:10) with a concentration of 1 mM. The resonance of 2,2,3,3-tetradeutero-3-trimethylsilylpropionic acid (TSP) was used as a chemical shift reference in the ^1^H NMR experiments (δ TSP = 0 ppm). The STD-NMR experiment was acquired using a 40:1 molar ratio of 8 μM VU-3C6 (GeneTex, Inc.) and 320 μM of 2-Hnv* in a 100% deuterated buffer containing 20 mM PBS, 20 mM NaCl, 0.09% NaN3, pD 7.1 at 310 K. The STD-NMR spectra (stddiffesgp pulse sequence from Bruker pulse program library) were acquired with 4160 scans in a matrix with 64k data points in t2 and in a spectral window of 12335.5 Hz centered at 2822.9 Hz. The selective saturation of the protein resonances (on resonance) was performed by irradiating at −0.5 ppm using a series of 40 Eburp2.1000-shaped 90° pulses (50 ms, 1 ms delay between pulses) for a total saturation time of 2 s. For the reference spectrum (off resonance), the samples were irradiated at 100 ppm. A peptide control experiment was performed for 2-Hnv* in the absence of VU-3C6, where residual STD signals for the methyl groups of Hnv, Ala, and GalNAc were observed, as well as for H6s of GalNAc. This result was considered and subtracted when analyzing the STD experiment to obtain an accurate epitope mapping of the interaction. The STD spectrum (ISTD) was obtained by subtracting the on-resonance spectrum (Ion) from the off-resonance spectrum (Ioff). The % of STD (ISTD/Ioff × 100) was estimated by comparing the intensity of the signals in the STD spectrum (ISTD) with the signal intensities of the reference spectrum (Ioff). To determine the STD-derived epitope map, the relative % of STD was calculated by setting to 100% the STD signal of the proton with the highest STD intensity and calculating the others accordingly. Some protons were not able to be assessed with accuracy due to the use of water suppression or low signal/noise ratio and displayed a blue circle in the STD-derived epitope maps. Moreover, the resonances overlapped on the ^1^H NMR spectrum were considered in STD estimation and are labeled as “*” (Figure S6).

### 2D ROESY Experiments

ROESY experiments were recorded on a Bruker Avance 400 spectrometer at 298 K and pH 6.5 in H_2_O/D_2_O (9:1). The experiments were conducted using phase-sensitive ge-2D ROESY with WATERGATE for H_2_O/D_2_O (9:1) spectra. ROESY intensities were normalized to the diagonal peak at zero mixing time. Distances involving NH protons were semiquantitatively determined by integrating the volume of the corresponding cross-peaks. The number of scans used was 32, and the mixing time was 500 ms (Figure S7).

### Molecular Dynamics Simulations of Glycopeptides 2-aThr* and 2-Hnv* in Water with Time-Averaged Restraints

The simulations were carried out with AMBER 18 package^55^ implemented with ff14SB,^56^ GAFF,^57^ and GLYCAM06j^58^ force fields. The parameters and charges for the unnatural amino acids were generated with the antechamber module of AMBER, using GAFF force field and AM1-BCC method^59^ for charges. Each molecule was then immersed in a water box with a 10 Å buffer of TIP3P water molecules.^60^ The system was neutralized by adding explicit counterions (Cl^–^). Before MD-tar productive simulations, we performed an equilibration protocol consisting of an initial minimization of the water box of 5000 steps, followed by a 2500-step minimization of the whole system. Then, the water box was heated at a constant volume until 300 K, using a time constant for the heat bath coupling of 1 ps. The equilibration finished with 200 ps of MD simulation without restraints, at a constant pressure of 1 bar and turning on the Langevin temperature scaling with a collision frequency of 1 ps. Nonbonded interactions were cut off at 8.0 Å and updated every 25 steps. Periodic boundary conditions and the Particle Mesh Ewald method^61^ were turned on in every step of the equilibration protocol to evaluate the long-range electrostatic forces, using a grid spacing of approximately 1 Å. The ROESY-derived distances (Table S4) were imposed as time-averaged constraints, applying a *r*^–6^ averaging. The equilibrium distance range was set to *r*_exp_ – 0.2 Å ≤ *r*_exp_ ≤ 0.2 Å. Trajectories were run at 298 K, with a decay constant of 20000 ps and a time step of 1 fs. The force constants *r*_k2_ and *r*_k3_ used in each case were 10 kcal·mol^–1^·Å^–2^. The overall simulation length was 200 ns. The coordinates were saved each 1 ps. Convergence within the equilibrium distance range was obtained in the simulations (Figures S9–S11).

### Crystallization

Expression and purification of scFv-SM3 have been described previously by us.^30^ Crystals were grown by sitting drop diffusion at 18 °C. The drops were prepared by mixing 0.5 μL of protein solution containing 15 mg/mL scFv-SM3 and 10 mM glycopeptide 2-Hnv* with 0.5 μL of the mother liquor. Crystals of scFv-SM3 with 2-Hnv* were grown in 20% PEG 3350, 0.2 M disodium hydrogen phosphate. The crystals were cryoprotected in mother liquor containing 15% ethylene glycol and frozen in a nitrogen gas stream cooled to 100 K.

### Animals and Immunization Protocol

Animal experiments were conducted at the Instituto de Medicina Molecular João Lobo Antunes (iMMLisboa-JLA, Portugal). Animal work was performed in strict accordance with the Portuguese Law (Portaria 1005/92) and the European Guideline 86/609/EEC and following the FELASA (Federation of European Laboratory Animal Science Associations) guidelines and recommendations concerning laboratory animal welfare. Furthermore, all animal experiments were approved by the Portuguese DGAV S39 and the IMM Animal Ethics Committee (authorization AEC_2014_07_GB_Vaccines). The vaccine candidate was administrated to 8-week-old Balb/c mice purchased from Charles River (Spain).

Mice were randomly divided into three experimental groups: 1-Thr* peptide control (*n* = 5), AuNP-1′-Thr* (*n* = 5), and AuNP-1′-Hnv* (*n* = 5). Animals were vaccinated receiving the selected vaccine candidate formulated in PBS buffer via intraperitoneal injection (50 μL, corresponding to 3 μg of peptide in all cases). The prime dose was administered to 8-week-old animals, followed by three additional booster doses at 21-day intervals. Five days after the last booster, mice were sacrificed, and whole blood was collected by heart puncture to obtain blood serum. We decided to use the nanoparticles without additional adjuvant to investigate the intrinsic adjuvanticity of the nanoparticle-based formulation.

### Quantification and Statistical Analysis

Statistical analysis was performed by using GraphPad Prism 10 software (GraphPad Software Inc.).

## Supporting Information Available

Synthesis and characterization of the (glyco)peptides studied in this work, BLI and SPR assays, microarray studies, STD-NMR, ROESY, circular dichroism curves, MD simulations, details of the X-ray structure of **2-Hnv*** bound to scFv-SM3, characterization of the nanoparticles, determination of the apparent *K*_D_ of AuNP-**1′-Thr*** and AuNP-**1′-Hnv*** and the antibody SM3, immunization protocol, antibody titers, and antibody isotypes (PDF)
