# Supplementary material for: Structure-Guided Approach for the Development of MUC1-Glycopeptide-Based Cancer Vaccines with Predictable Responses
Source: JACS Au. 2023 Nov 21;4(1):150–63. doi: 10.1021/jacsau.3c00587 (PMC10807005; doi:10.1021/jacsau.3c00587)
Supplement: Supplementary file 1 — au3c00587_si_001.pdf [file au3c00587_si_001.pdf]

Supporting Information

A Structure-Guided Approach for the Development of MUC1-Glycopeptide-Based Cancer Vaccines with Predictable Responses

Iris A. Bermejo,[1,#] Ana Guerreiro,[2,#] Ander Eguskiza,[3,#] Nuria Martínez-Sáez,[1,4,#] Foivos S. Lazaris,[1] Alicia Asín,[1] Víctor J. Somovilla,[1] Ismael Compañón,[1] Tom K. Raju,[5] Srdan Tadic,[5] Pablo Garrido,[5] Josune García-Sanmartín,[5] Vincenzo Mangini,[6] Ana S. Grosso,[7,8] Filipa Marcelo,[7,8] Alberto Avenoza,[1] Jesús H. Busto,[1] Fayna García-Martín,[1] Ramón Hurtado-Guerrero,[9,10,11] Jesús M. Peregrina,[1] Gonçalo J. L. Bernardes,[2,12,*] Alfredo Martínez,[5,*] Roberto Fiammengo,[3,6,*] and Francisco Corzana[1,*]

[1]Department of Chemistry and Instituto de Investigación en Química de la Universidad de La Rioja (IQUR), Universidad de La Rioja, Logroño, 26006, Spain.

[2]Instituto de Medicina Molecular João Lobo Antunes, Faculdade de Medicina, Universidade de Lisboa, Lisboa, 1649-028, Portugal.

[3]Department of Biotechnology, University of Verona, Verona, 37134, Italy.

[4]Departamento de Tecnología y Química Farmacéuticas, Universidad de Navarra, Pamplona, 31008, Spain.

[5]Angiogenesis Group, Oncology Area, Center for Biomedical Research of La Rioja (CIBIR), Logroño, 26006, Spain.

[6] Center for Biomolecular Nanotechnologies@UniLe, Istituto Italiano di Tecnologia (IIT), Arnesano, Lecce, 73010, Italy.

[7]Applied Molecular Biosciences Unit UCIBIO, Department of Chemistry, NOVA School of Science and Technology, Caparica, 2829-516, Portugal.

[8]Associate Laboratory i4HB - Institute for Health and Bioeconomy, NOVA School of Science and Technology, Caparica, 2829-516, Portugal.

[9]Institute of Biocomputation and Physics of Complex Systems, University of Zaragoza, Zaragoza, 50018, Spain.

[10]Copenhagen Center for Glycomics, Department of Cellular and Molecular Medicine, Faculty of Health Sciences, University of Copenhagen, Copenhagen, 2200, Denmark.

[11]Fundación ARAID, Zaragoza, 50018, Spain.

[12]Yusuf Hamied Department of Chemistry, University of Cambridge, Cambridge, CB2 1EW, UK.

[#]These authors contributed equally to this work.

Correspondence should be addressed to F.C., R.F., A.M. or G.J.L.B: francisco.corzana@unirioja.es; roberto.fiammengo@univr.it; amartinezr@riojasalud.es; gb453@cam.ac.uk.

1. Reagents and general procedures

Commercial reagents were used without further purification. Analytical thin layer chromatography (TLC) was performed on Macherey-Nagel precoated aluminium sheets with a 0.20 mm thickness of silica gel 60 with fluorescent indicator UV254. TLC plates were visualized with UV light and by staining with phosphomolybdic acid (PMA) solution (5 g of PMA in 100 mL of absolute ethanol) or sulfuric acid-ethanol solution (1:20). Purification of products by column chromatography was performed on silica gel (230−400 mesh).

^{1}H and ^{13}C NMR spectra were measured with a Bruker ARX-300 or a Bruker Avance-400 spectrometer with TMS as the internal standard. Multiplicities are quoted as singlet (s), broad singlet (br s), doublet (d), doublet of doublets (dd), triplet (t), or multiplet (m). Signals were assigned using COSY and HSQC experiments. NMR chemical shifts (δ) are reported in ppm and coupling constants (*J*) in Hz. High-resolution electrospray mass (ESI) spectra were recorded on a microTOF spectrometer; accurate mass measurements were achieved using sodium formate as an external reference. Peptides were dissolved in 0.1% TFA solution for a final concentration of 10 µM.

HR MALDI: A mixture of peptide and α-cyano-4-hydroxycinnamic acid (HCCA) (1:1 v/v) was prepared and 0.5 µL were spotted onto a ground steel target.

2D NMR experiments. Magnitude-mode ge-2D COSY spectra were acquired with gradients using the *cosygpqf* pulse program with a pulse width of 90º. Phase-sensitive ge-2D HSQC spectra were acquired using z-filter and selection before t1, removing the decoupling during the acquisition using the *invigpndph* pulse program with CNST2 (JHC)=145.

2. Synthesis

Synthetic route followed to obtain compound 7α

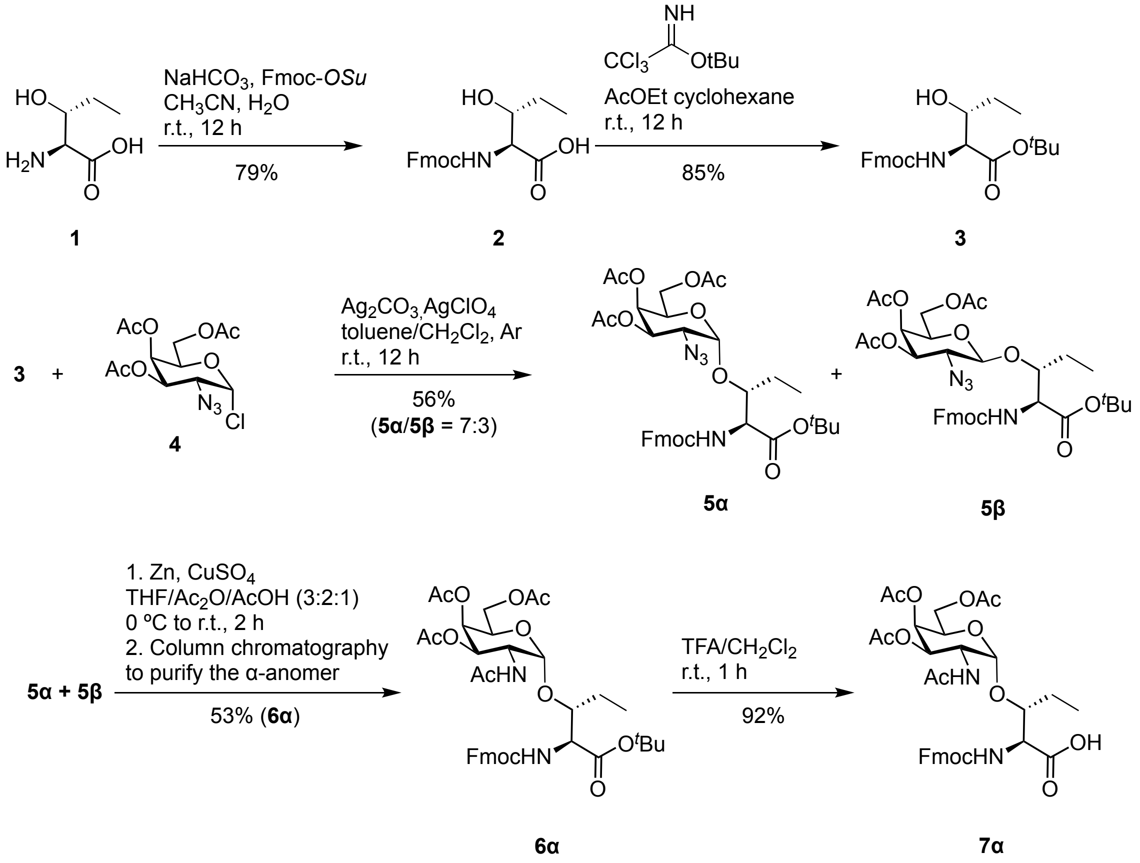

Synthesis of compound **3**

Commercially available (2*S*,3*R*)-3-hydroxynorvaline (Hnv, **1**, 200 mg, 1.50 mmol) was dissolved in H_2O (7 mL), and $NaHCO_3$ (252 mg, 3 mmol) was then added. The resulting mixture was stirred at rt until complete dissolution was achieved. The reaction mixture was diluted with acetonitrile (15 mL), followed by adding Fmoc–O*Su* (761 mg, 2.25 mmol). The white suspension was stirred vigorously at rt overnight. Acetonitrile was then removed under reduced pressure, and the aqueous solution was extracted with Et_2O, followed by acidification and subsequent extraction with a mixture of $CHCl_3$/^{i}PrOH (3:1). The organic layer was dried with Na_2SO_4 and concentrated, and the corresponding amino acid derivative **2** (420 mg, 79%) was obtained without further purification. *tert*-Butyl 2,2,2-trichloroacetimidate (423 µL, 2.36 mmol) dissolved in cyclohexane (2.5 mL) was added drop-by-drop to a solution of derivative **2** (420 mg, 1.18 mmol) in ethyl acetate (6.30 mL) and cyclohexane (2.5 mL) and the mixture was stirred vigorously overnight. The reaction mixture was then washed twice with a saturated solution of $NaHCO_3$ (10 mL). By-products crystallized out of CH_2Cl_2 and were separated followed by recovering of the supernatant that was concentrated and purified by column chromatography

(hexane/ethyl acetate, 6:4) to give protected amino acid **3** as a white foam (413 mg, 85%).

HRMS (ESI+) m/z: calcd. for $C_{24}H_{29}NNaO_5$ $[M+Na]^+$: 434.1938; found: 434.1920.

^{1}H NMR (300 MHz, $CDCl_3$) δ (ppm): 0.99 (t, 3H, *J* = 7.3 Hz, CH_3 Hnv), 1.48 – 1.52 (m, 11H, CH_2 Hnv, $C(CH_3)_3$), 4.00 (t, 1H, *J* = 5.9 Hz, Hβ), 4.21 – 4.26 (m, 1H, CHFmoc), 4.30 (d, 1H, *J* = 9.3 Hz, Hα), 4.41 (m, 2H, CH_2Fmoc), 7.29 – 7.34 (m, 2H, Fmoc), 7.38 – 7.43 (m, 2H, Fmoc), 7.59 – 7.63 (m, 2H, Fmoc), 7.75 – 7.78 (m, 2H, Fmoc).

^{13}C NMR (75 MHz, $CDCl_3$) δ (ppm): 10.1 (CH_3 Hnv), 26.9 (CH_2 Hnv), 28.0 ($C(\underline{C}H_3)$), 47.2 (CH-Fmoc), 58.0 (Cα), 67.1 (CH_2-Fmoc), 73.8 (Cβ), 82.6 ($\underline{C}(CH_3)$), 119. 9, 120.0, 125.1, 125.1, 127.1, 127.1, 127.7, 127.7, 143.3, 143.3, 143.8, 143.9 (Fmoc), 156.7 (NCO_2), 170.6 (CO_2).

Synthesis of compounds **5α** and **5β**

Compound **3** (900 mg, 2.26 mmol) was dissolved in a mixture of dry toluene/CH_2Cl_2 (18 mL, 1:1) under an argon atmosphere with molecular sieves (4 Å). After stirring for 1 h, Ag_2CO_3 (936 mg, 3.39 mmol) and $AgClO_4$ (25 mg, 0.12 mmol), were added at 0 ºC. The reaction mixture was kept under vigorous stirring for 30 min, and glycosidic donor **4** (1.38 g, 3.94 mmol)[1] dissolved in 11 mL of 4:7 toluene/CH_2Cl_2 was added. The reaction mixture was then stirred overnight at rt in the absence of light. The reaction mixture was filtered through Celite, concentrated, and purified by chromatography column in toluene/acetone (9:1), yielding a 7:3 mixture of α and β anomers (887 mg, 56%) as a white foam. No separation of the two isomers was attempted at this stage.

HRMS (ESI+) m/z: calcd. for $C_{36}H_{45}N_4NaO_{12}$ $[M+H]^+$: 725.3028; found: 725.3020.

Synthesis of compound **6α**

The 7:3 mixture of compounds **5α** and **5β** (230 mg, 0.317 mmol) was dissolved in 3:2:1 THF/AcOH/Ac_2O (8 mL). Keeping the mixture at 0 ºC, activated Zn (210 mg, 3.21 mmol) and 0.3 mL of a saturated solution of $CuSO_4$ were added and the reaction was stirred for 2 h. It was then filtered through Celite, washed with a saturated solution of $NaHCO_3$ (2x5 mL), dried and evaporated. After column chromatography using hexane/ ethyl acetate (7:3) as eluent, pure **6α** (α anomer) was obtained as a white foam (125 mg, 53%).

HRMS (ESI+) m/z: calcd. for $C_{38}H_{49}N_2O_{13}$ $[M+H]^+$: 741.3229; found: 741.3230.

^{1}H NMR (400 MHz, $CDCl_3$) δ (ppm): 0.98 (t, 3H, *J* = 7.3 Hz, CH_3 Hnv), 1.47 (s, 9H, $C(CH_3)_3$), 1.61 – 1.70 (m, 2H, CH_2 Hnv), 2.00 (s, 3H, $NHCOCH_3$), 2.01, 2.05, 2.17 (s, 9H, $3COCH_3$), 3.86 – 3.94 (m, 1H, Hβ), 4.10 (m, 2H, CH_2Fmoc), 4.24 – 4.30 (m, 2H, H_{5S}, CHFmoc), 4.40 (d, 1H, *J* = 8.7 Hz, Hα), 4.44 – 4.51 (m, 2H, $2H_{6S}$), 4.61 – 4.68 (m, 1H, H_{2S}), 4.92 (d, 1H, *J* = 3.4 Hz, H_{1S}), 5.10 (dd, 1H, *J* = 11.3, 2.9 Hz, H_{3S}), 5.38 – 5.41 (s,

1H, H_{4S}), 7.31 – 7.36 (m, 2H, Fmoc), 7.39 – 7.43 (m, 2H, Fmoc), 7.61 – 7.67 (m, 2H, Fmoc), 7.76 – 7.80 (m, 2H, Fmoc). ‘s’ stands for sugar moiety.

^{13}C NMR (101 MHz, $CDCl_3$) δ (ppm): 10.1 (CH_3 Hnv), 20.6, 20.7, 20.8 ($\underline{C}H_3CO$), 23.3 ($NHCO\underline{C}H_3$), 24.8 (CH_2 Hnv), 28.1 ($C(\underline{C}H_3)$), 47.2 (CH-Fmoc), 47.3 (C_{2S}), 55.6 (Cα), 62.2 (CH_2-Fmoc), 67.3 (C_{6S}), 67.5 (C_{4S}), 67.5 (C_{5S}), 68.8 (C_{3S}), 82.5 (Cβ), 83.4 ($\underline{C}(CH_3)$)), 99.8 (C_{1S}), 120.0, 120.1, 125.0, 125.1, 127.1, 127.1, 127.8, 127.8, 141.4, 141.4, 143.7, 143.9 (Fmoc), 156.3 (NCO_2), 170.3, 170.4, 170.4, 170.5, 170.9 (CO_2). ‘s’ stands for sugar moiety.

Synthesis of compound **7α**

A solution of derivative **6α** (179 mg, 0.242 mmol) in a mixture of CH_2Cl_2/TFA (10 mL, 1:1) was stirred for 2 h and then concentrated until TFA was removed. Building block **7α** was obtained as a white foam (152 mg, 92%) and used without further purification in solid-phase peptide synthesis.

HRMS (ESI) (m/z): cald. for $C_{34}H_{41}N_2O_{13}^+[M+H]^+$: 685.2603; found: 685.2599.

^{1}H NMR (300 MHz, CD_3OD) δ (ppm): 0.93 (*t*, 3H, *J* = 7.4 Hz, CH_3 Hnv), 1.47 – 1.78 (m, 2H, CH_2 Hnv), 1.93 (s, 3H, $COCH_3$), 1.94 (s, 3H, $COCH_3$), 2.04 (s, 3H, $COCH_3$), 2.14 (s, 3H, $COCH_3$), 4.05 – 4.18 (m, 3H, Hβ, $2H_{6S}$), 4.20 – 4.54 (m, 5H, Hα, H_{5s}, H_{2s}, 1 CH_2Fmoc, CHFmoc), 4.56 – 4.68 (m, 1H, 1 CH_2Fmoc), 4.94 (d, 1H, *J* = 4.0 Hz, H_{1s}), 5.06 (dd, 1H, *J* = 11.6, 3.2, H_{3s}), 5.38 – 5.41 (m, 1H, H_{4s}), 7.28 – 7.43 (m, 4H, Fmoc), 7.65 – 7.72 (m, 2H, Arom), 7.78 – 7.81 (m, 2H, Arom). ‘s’ stands for sugar moiety.

^{13}C NMR (75 MHz, CD_3OD) δ (ppm): 8.9 (CH_3 Hnv), 19.1, 19.2, 19.3 ($\underline{C}H_3CO$), 21.5 ($NHCO\underline{C}H_3$), 25.0 (CH_2 Hnv), 46.8 (CHFmoc), 47.3 (C_{2S}), 54.9 (Cα), 61.9 (C_{6s}), 66.2 (CH_2Fmoc), 66.8 (C_{5s}), 67.4 (C_{4S}), 68.2 (C_{3S}), 81.7 (Cβ), 99.4 (C_{1S}), 119.5, 119.6, 124.6, 124.7, 126.8, 127.39, 141.3, 143.7, 144.0 (Arom), 157.7 (NCO_2), 170.5, 170.7, 172.2, 172.5 (CO_2). ‘s’ stands for sugar moiety. Some of the signals corresponding to the aromatic carbons and the CO groups are overlapping.

Synthetic route followed to obtain compound 13

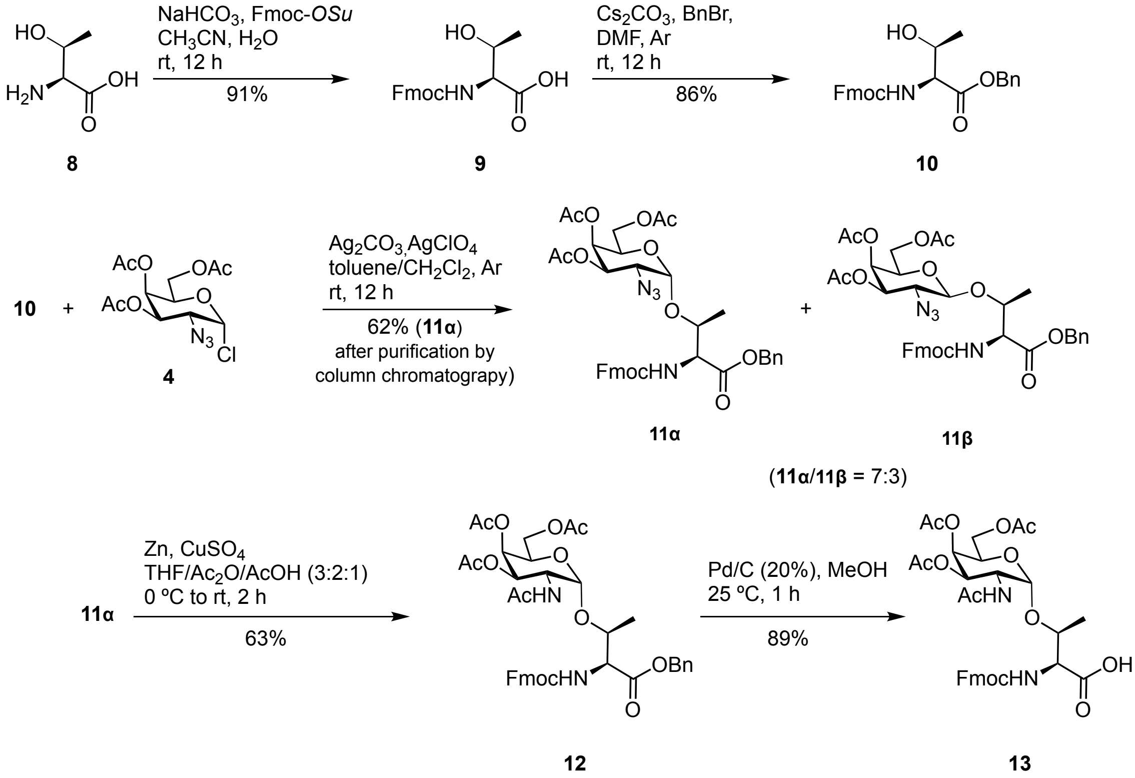

<u>Synthesis of compound **10**</u>

Following the same methodology described for **2** but starting from commercially available *allo*-threonine (aThr, **8**, 1.00 g, 8.39 mmol), compound **9** was obtained as a white solid in a 91% yield (2.60 g). This compound (2.60 g, 7.62 mmol) was dissolved in DMF (15 mL) under an argon atmosphere, Cs_2CO_3 (3.72 g, 11.42 mmol) was added at rt and stirred for 1 h, followed by the addition of benzyl bromide (1.1 mL, 9.14 mmol) and stirred for 12 h at rt. The reaction mixture was poured onto a saturated aqueous solution of LiBr (100 mL), extracted with ethyl acetate (50 mL), and washed with water (50 mL) and brine (25 mL). The organic layer was dried (Na_2SO_4) and filtered and the filtrate was concentrated in vacuum. The residue was purified by silica gel column chromatography (hexane/ethyl acetate, 1:1) to give compound **10** (3.12 g, 86%) as a white solid.

HRMS (ESI+) m/z: calcd. for $C_{26}H_{25}NO_5$ $[M+H]^+$: 432.1805 found: 432.1810.

^{1}H NMR (300 MHz, $CDCl_3$) δ (ppm): 1.17 (d, 3H, *J* = 6.1 Hz, CH_3 aThr), 2.79 (s, 1H, OH), 4.10 – 4.26 (m, 2H, Hβ aThr, CH-Fmoc), 3.36 – 4.46 (m, 2H, CH_2-Fmoc), 4.49 (d, 1H, *J* = 3.8 Hz, Hα aThr), 5.08 – 5.31 (m, 2H, CH_2-Bn), 7.19 – 7.47 (m, 9H, Arom), 7.51 – 7.67 (m, 2H, Arom), 7.70 – 7.81 (m, 2H, Arom).

^{13}C NMR (75 MHz, $CDCl_3$) δ (ppm): 18.9 (CH_3 aThr), 47.2 (CH-Fmoc), 59.5 (Cα aThr), 67.3 (CH_2-Fmoc), 67.6(CH_2-Bn), 69.0(Cβ aThr), 120.0, 120.1, 125.1, 125.1, 127.1, 127.1, 127.8, 127.8, 128.4, 128.4, 128.6, 128.7, 128.7, 135.0, 141.4, 143.7, 143.8 (Bn and Fmoc), 156.7 (NCO_2), 170.2 (CO_2).

Synthesis of compound **11α**

Following the same methodology described for anomers **5α** and **5β** but starting from derivative **10** (869 mg, 2.01 mmol), compound **11α** (928 mg, 62%) was obtained as a white foam after purification by column chromatography in toluene/acetone (9:1).

HRMS (ESI+) m/z: calcd. for $C_{38}H_{40}N_4O_{12}^+$ $[M+H]^+$: 745.2715 found: 745.2746.

^{1}H NMR (300 MHz, $CDCl_3$) δ (ppm): 1.40 (d, 3H, J = 6.5 Hz, CH_3 aThr), 1.95 (s, 3H, $COCH_3$), 2.09 (s, 3H, $COCH_3$), 2.18 (s, 3H, $COCH_3$), 3.62 (dd, 1H, J = 11.2, 3.3 Hz, H_{2S}), 4.00 – 4.10 (m, 2H, $2H_{6S}$), 4.10 – 4.21 (m, 1H, Hβ aThr), 4.25 (dd, 1H, J = 15.4, 8.5 Hz, CH-Fmoc), 4.32 – 4.47 (m, 2H, CH_2-Fmoc, H_{5S}), 4.49 – 4.66 (m, 2H, CH_2 Fmoc, Hα aThr), 4.99 (d, 1H, J = 3.3 Hz, H_{1S}), 5.21 – 5.37 (m, 3H, CH_2-Bn, H_{3S}), 5.40 – 5.48 (m, 1H, H_{4S}), 7.18 – 7.50 (m, 9H, Arom), 7.63 – 7.72 (m, 2H, Arom), 7.73 – 7.85 (m, 2H, Arom). 's' stands for sugar moiety.

^{13}C NMR (75 MHz, $CDCl_3$) δ (ppm): 16.9 (CH_3 aThr), 20.5, 20.6, 20.7 (3 $CO\underline{C}H_3$), 47.2 (CH Fmoc), 57.4 (C_{2S}), 58.6(Cα aThr), 61.9(C_{6S}), 67.1 (CH_2 Fmoc), 67.4($C_{5S,}$), 67.7, 67.7, 67.8 (CH_2-Bn, C_{4S}, C_{3S}), 78.2(Cβ aThr), 98.5 (C_{1S}), 120.0, 120.1, 125.1, 125.2, 127.1, 127.1, 127.7, 127.8, 128.3, 128.7, 128.7, 128.7, 129.1, 134.9, 141.4, 143.8, 143.9 (Bn and Fmoc), 155.9 (NCO_2), 168.9, 169.6, 170.0, 170.4 (CO_2). 's' stands for sugar moiety.

Synthesis of compound **12**

Compound **11α** (620 mg, 0.832 mmol) was dissolved in a mixture of THF/AcOH/Ac_2O (12 mL, 3:2:1). Keeping the mixture at 0 ºC, activated Zn (707 mg, 10.82 mmol) and 1.25 mL of a saturated solution of $CuSO_4$ were added, and the reaction was stirred for 2 h. The reaction mixture was filtered through Celite, washed with a saturated solution of $NaHCO_3$ (2 x 15 mL), dried and evaporated. After column chromatography (hexane/ethyl acetate, 7:3), derivative **12** was obtained as a white foam (580 mg, 91%).

HRMS (ESI+) m/z: calcd. for $C_{40}H_{44}N_2O_{13}$ $[M+H]^+$: 761.2916 found: 761.2914.

^{1}H NMR (300 MHz, $CDCl_3$) δ (ppm): 1.27 (d, 3H, J = 6.0 Hz , CH_3 aThr), 1.92 (s, 3H, $COCH_3$), 1.97 (s, 3H, $COCH_3$), 2.05 (s, 3H, $COCH_3$), 2.20 (s, 3H, $COCH_3$), 3.97 – 4.12 (m, 3H, $2H_{6S}$, Hβ aThr), 4.18 – 4.30 (m, 2H, CH-Fmoc, H_{5S}), 4.41 – 4.61 (m, 4H, CH2-Fmoc, Hα aThr, H_{2S}), 4.92 (s, 1H, H_{1S}), 4.95 – 5.05 (m, 1H, H_{3S}), 5.14 – 5.36 (m, 3H, CH_2-Bn, H_{4S}), 5.45 (d, 1H, J = 8.7 Hz, NHAc), 7.27 – 7.49 (m, 9H, Arom), 7.55 – 7.68 (m, 2H, Arom), 7.75 – 7.84 (m, 2H, Arom). 's' stands for sugar moiety.

^{13}C NMR (75 MHz, $CDCl_3$) δ (ppm): 17.1 (CH_3 aThr), 20.6, 20.7, 20.8, 23.2 (4 $CO\underline{C}H_3$), 47.2 (CH-Fmoc), 47.9 (C_{2S}), 58.7 (Cα aThr), 62.1 (C_{6S}), 67.4 (CH_2-Fmoc), 67.6, 68.1 (CH_2-Bn, C_{3S}), 97.4 (C_{1S}), 120.0, 125.0, 127.1, 127.7, 128.8, 128.9, 135.0, 141.4, 143.7

(Bn and Fmoc), 155.9 (NCO_2),169.9, 170.3, 170.5, 170.9 (CO_2). ‘s’ stands for sugar moiety.

<u>Synthesis of compound **13**</u>

A solution of derivative **12** (580 mg, 0.762 mmol) in 20 mL of MeOH, together with 3-4 drops of HCl 37.5%, was treated with 10% Pd/C (116 mg) and shaken under H_2 atmosphere for 1 h and at rt. The catalyst was then removed through Celite filtration. The solution was concentrated before column chromatography in CH_2Cl_2/MeOH (9:1, 0.1% AcOH), yielding building block **13** as a white foam (460 mg, 89%), which was used without further purification in solid-phase peptide synthesis.

HRMS (ESI+) m/z: calcd. for $C_{33}H_{38}N_2O_{13}^+$ [M+H] $^+$: 671.2447 found: 671.2443.

^{1}H RMN (300 MHz, $CDCl_3$) δ (ppm): 1.37 (d, 3H, *J* = 5.6 Hz, , CH_3 aThr), 1.90 – 2.07 (m, 9H, 3 $COCH_3$), 2.21 (s, 3H, $COCH_3$), 3.99 – 4.11 (m, 2H, Hβ, H_{6S}), 4.12 – 4.2 (m, 1H, H_{6S}), 4.24 (t, 1H, *J* = 6.5 Hz, CHFmoc), 4.35 – 4.63 (m, 5H, Hα, H_{5S}, CH_2Fmoc, H_{2S}), 5.01 (s, 1H, H_{1S}), 5.05 – 5.18 (m, 1H, H_{3S}), 6.22 (d, 1H, *J* = 8.3 Hz, NHFmoc), 6.45 (d, 1H, *J* = 8.7 Hz, NHAc), 7.25 – 7.49 (m, 4H Arom), 7.54 – 7.68 (m, 2H, Arom), 7.54 – 7.73 (m, 2H, Arom). ‘s’ stands for sugar moiety.

^{13}C RMN (75 MHz, $CDCl_3$) δ (ppm): 16.9 (CH_3 aThr), 20.6, 20.8, 22.9 ($CO\underline{C}H_3$), 47.1 (CHFmoc), 47.9 (C_{2S}), 58.5 (Cα), 62.1 (C_{6S}), 67.1 (CH_2Fmoc, C_{4S}), 68.3 (C_{3S}), 72.8 (Cβ), 120.0, 125.0, 127.0, 127.0, 127.8, 141.3, 143.6 (Arom), 156.0, (NCO_2), 170.9, 171.24 (CO). ‘s’ stands for sugar moiety. Some of the signals corresponding to the aromatic carbons and the CO groups are overlapping.

Solid-phase peptide synthesis (SPPS). (Glyco)peptides were synthesized by stepwise microwave-assisted solid-phase peptide synthesis on a Liberty Blue synthesizer using the Fmoc strategy on Rink Amide MBHA resin (0.1 mmol). Fmoc-Thr[GalNAc$(Ac)_3$-α-D]-OH was synthesized as described in the literature.[1] This compound and the glycosylamino acids synthesized in this work (2.0 equiv) were manually coupled using HBTU [(2-(1*H*-benzotriazol-1-yl)-1,1,3,3-tetramethyluronium hexafluorophosphate] (0.9 equiv) and diisopropyl ethyl amine –DIPEA– (2.0 equiv) , while all other Fmoc amino acids (5.0 equiv) were automatically coupled using oxyma pure/DIC (*N*,*N*'-diisopropylcarbodiimide). For glycopeptides **1’-Thr** and **1’-Hnv**, the Cys residue was *N*-acetylated by the treatment of the protected glycopeptides attached to the resin with acetic anhydride/pyridine (2:1) at rt for 2 h. The *O*-acetyl groups of GalNAc moiety were removed treating the resin-bound peptide with a mixture of NH_2NH_2/MeOH (7:3) 3x5 mL. (Glyco)peptides were then released from the resin, along with removal of the acid-sensitive sidechain protecting groups, using TFA 95%, triisopropylsilane (TIS) 2.5%, and

H_2O 2.5% (3 mL) for 3 h at 25 ºC or 30 min at 37 ºC. For glycopeptides **1'-Thr** and **1'-Hnv**, a solution of TFA/TIS/H_2O/EDT (92.5:2.5:2.5:2.5, 3 mL) was used for 3 h at 25 ºC without microwave irradiation. (Glyco)peptides were then precipitated with cold diethyl ether (20 mL) and centrifuged for 6 min at 6500 rpm. The supernatant solution was discarded, and this process was repeated twice. Finally, (glyco)peptides were dried and redissolved in water to be purified by reverse phase HPLC on a Phenomenex Luna C18(2) column (10 μm, 250 mm x 21.2 mm) with a flow rate of 10 mL/min. UV detection was done at 212 nm.

The synthesis and characterization of compounds **1-Thr**, **1-Thr***, **2-Ser**, **2-Ser***, **2-Thr,** and **2-Thr*** have been previously described.[2]

<u>Peptide **1-aThr**</u>

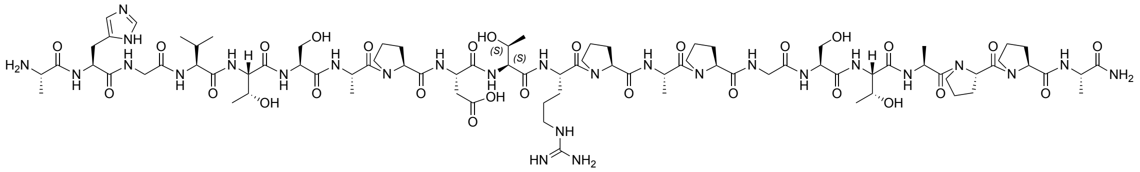

HRMS (ESI+) m/z: calcd. for $C_{83}H_{133}N_{27}O_{28}$: $[M+2H]^{2+}$: 978.9906 found: 978.9912. Calcd. for $[M+3H]^{3+}$: 652.9938 found: 652.9946. Semipreparative HPLC on a Phenomenex Luna C18(2) column (10 μm, 250 mm x 21.2 mm), Rt = 25.1 min (Grad: water 0.1% TFA/acetonitrile (95:5) → (77:23), 35 min, λ = 212 nm).

<u>Glycopeptide **1-aThr***</u>

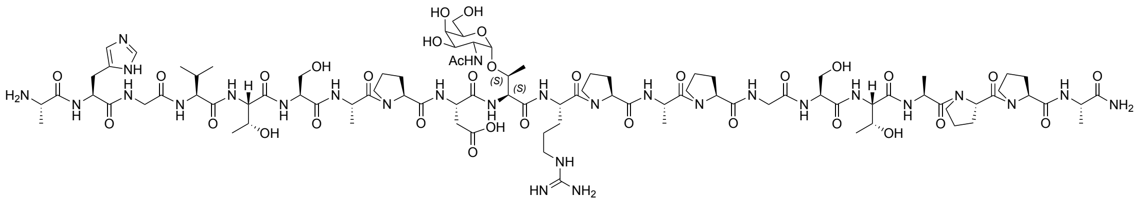

HRMS (ESI+) m/z: calcd. for $C_{91}H_{146}N_{28}O_{33}$: $[M+3H]^{3+}$: 720.6869 found: 720.6875. Semipreparative HPLC on a Phenomenex Luna C18(2) column (10 μm, 250 mm x 21.2 mm), Rt = 16.7 min (Grad: water 0.1% TFA/acetonitrile (90:10) → (82:18), 30 min, λ = 212 nm).

<u>Peptide **1-Hnv**</u>

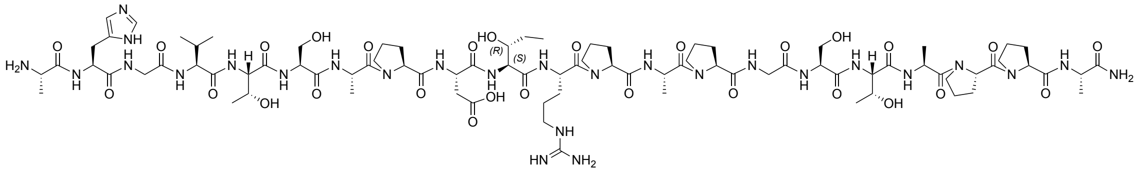

HRMS (ESI+) m/z: calcd. for $C_{84}H_{138}N_{27}O_{28}$: $[M+3H]^{3+}$: 658.0011 found: 658.0016. Semipreparative HPLC on a Phenomenex Luna C18(2) column (10 μm, 250 mm x 21.2 mm), Rt = 27.2 min (Grad: water 0.1% TFA/acetonitrile (95:5) → (77:23), 35 min, λ = 212 nm).

<u>Glycopeptide **1-Hnv***</u>

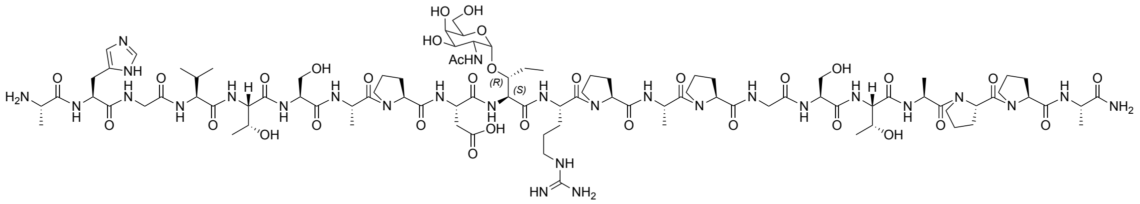

HRMS (ESI+) m/z: calcd. for $C_{92}H_{151}N_{28}O_{33}$: $[M+3H]^{3+}$: 725.3658 found: 725.3666. Semipreparative HPLC on a Phenomenex Luna C18(2) column (10 μ, 250 mm x 21.2 mm), Rt = 19.2 min (Grad: water 0.1% TFA/acetonitrile (90:10) → (86:14), 20 min, λ = 212 nm).

<u>Peptide **1-MeSer**</u>

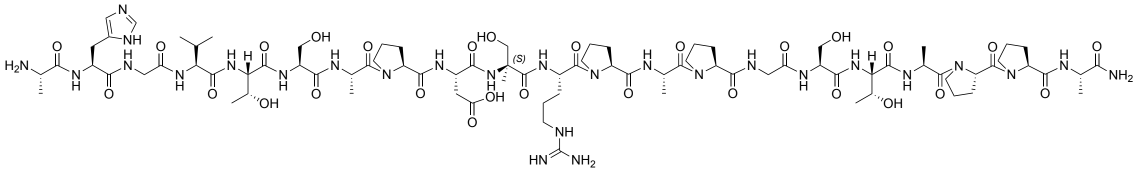

HRMS (ESI+) m/z: calcd. for $C_{91}H_{148}N_{28}O_{33}$ $[M+2H]^{2+}$: 978.9979 found: 978.9983. Calcd. for $[M+3H]^{3+}$: 653.0011 found: 653.0031. Semipreparative HPLC on a Phenomenex Luna C18(2) column (10 μm, 250 mm x 21.2 mm), Rt = 31.5 min (Grad: water 0.1% TFA/acetonitrile (95:5) → (77:23), 50min, λ = 212 nm).

<u>Glycopeptide **1-MeSer***</u>

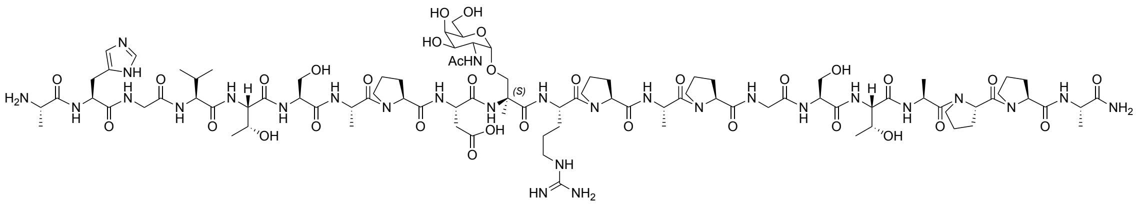

HRMS (ESI+) m/z: calcd. for $C_{91}H_{148}N_{28}O_{33}$ $[M+2H]^{2+}$: 1080.5376 found:1080.5374. Calcd. for $[M+3H]^{3+}$: 720.6942 found: 720.6953. Semipreparative HPLC on a Phenomenex Luna C18(2) column (10 μm, 250 mm x 21.2 mm), Rt = 16.2 min (Grad: water 0.1% TFA/acetonitrile (90:10) → (82:18), 20 min, λ = 212 nm).

<u>Glycopeptide **2-aThr***</u>

This compound was synthesized as an *N*-acetylated derivate.

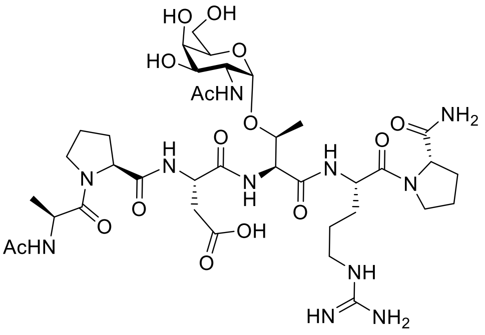

HRMS (ESI+) m/z: calcd. for $C_{35}H_{59}N_{11}O_{14}$ $[M+1H]^+$: 857.4243 found: 858.4289 (non-acetylated derivative). Semipreparative HPLC on a Phenomenex Luna C18(2) column (10 µm, 250 mm x 21.2 mm), Rt = 11.0 min (Grad: water 0.1% TFA/acetonitrile (90:10) → (88:12), 14 min, λ = 212 nm).

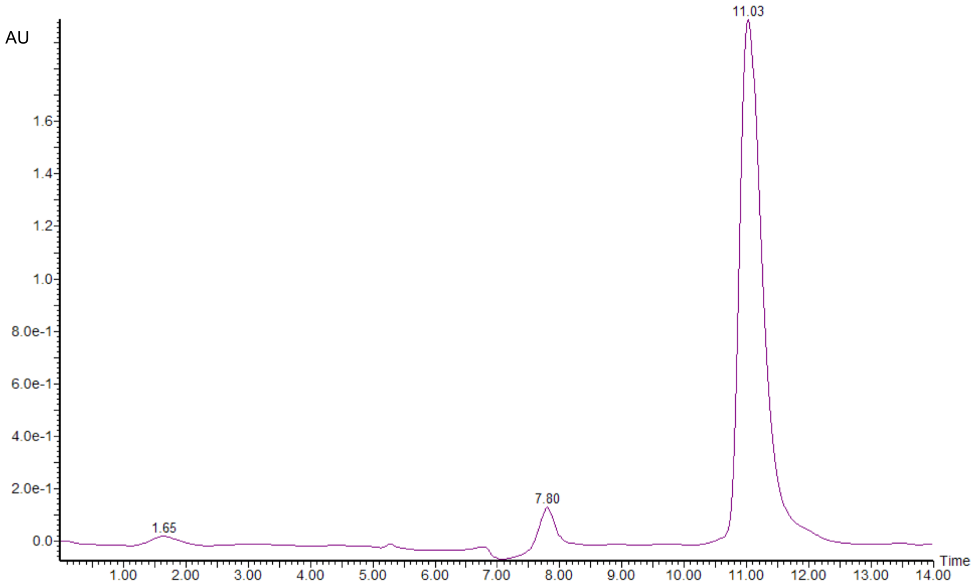

HPLC chromatogram of glycopeptide <u>2-aThr</u>*

^{1}H NMR (400 MHz, D_2O) δ (ppm): 1.04 (d, 3H, *J* = 6.1 Hz, Me_{aThr}), 1.23 (d, 3H, *J* = 7.1 Hz, Me_{Ala}), 1.51 – 1.57 (m, 2H, $2H\gamma_{Arg}$), 1.72 – 1.81 (m, 2H, $2H\beta_{Arg}$), 1.81– 2.02 (m, 12H, $NHCOC\underline{H}_{3\ GalNAc}$, $NHCOC\underline{H}_{3\ Ala}$, $2H\gamma_{Pro7}$, $2H\gamma_{Pro11}$, $H\beta_{Pro7}$, $H\beta_{Pro11}$), 2.14 – 2.23 (m, 2H, $H\beta_{Pro7}$, $H\beta_{Pro11}$), 2.71 – 2.84 (m, 2H, $2H\beta_{Asp}$), 3.08 – 3.12 (m, 2H, $2H\delta_{Arg}$), 3.49 – 3.57 (m, 5H, $H\delta_{Pro7}$, $H\delta_{Pro11}$, H_{5S}, $2H_{6S}$), 3.62 (m, 1H, *J* = 7.1, 4.0 Hz, H_{3S}), 3.67 – 3.74 (m, 3H, $H\delta_{Pro7}$, $H\delta_{Pro11}$, H_{4S}), 3.90 – 3.97 (m, 1H, $H\beta_{aThr}$), 4.02 (dd, 1H, *J* = 11.1, 3.8 Hz, H_{2S}), 4.26 – 4.33 (m, 2H, $H\alpha_{Pro11}$, $H\alpha_{Pro7}$), 4.35 (d, 1H, *J* = 8.4 Hz, $H\alpha_{aThr}$), 4.41 – 4.47 (m, 1H, $H\alpha_{Ala}$),

4.55 (t, 1H, J = 6.7 Hz, Hα_{Asp}), 4.63 – 4.64 (m, 1H, Hα_{Arg}), 4.91 (d, 1H, J = 3.9 Hz, H_{1S}). 's' stands for sugar moiety.

^{1}H NMR (400 MHz, D_2O/H_2O, 1:9, amide region) δ (ppm): 7.02 (s, 1H, NH_{Pro}), 7.16 – 7.19 (m, 1H, NH_{Arg}), 7.74 (s, 1H, NH_{Pro}), 8.04 (d, 1H, J = 9.1 Hz, N$\underline{H}$COCH_3), 8.24 – 8.28 (m, 2H, NH_{Ala}, NH_{aThr}), 8.52 – 8.57 (m, 2H, NH_{Asp}, NH_{Arg}).

^{13}C NMR (101 MHz, D_2O) δ (ppm): 14.8 (Me_{aThr}), 15.3 (Me_{Ala}), 21.4 (NHCOC$\underline{H}_{3Ala}$), 21.9 (NHCOC$\underline{H}_{3\ aThr}$), 24.0 (Cγ_{Arg}), 24.6 (Cγ_{Pro11}), 24.7 (Cγ_{Pro7}), 28.0 (Cβ_{Arg}), 29.3 (Cβ_{Pro11}), 29.6 (Cβ_{Pro7}), 35.2 (Cβ_{Asp}), 40.5 (Cδ_{Arg}), 47.7, 47.8, 47.9 (Cδ_{Pro7}, Cδ_{Pro11}, Cα_{Ala}), 49.8 (C_{2S}), 50.1 (Cα_{Asp}), 50.8 (Cα_{Arg}), 57.9 (Cα_{aThr}), 60.2 (Cα_{Pro11}), 60.4 (Cα_{Pro7}), 60.5 (C_{6S}), 67.5 (C_{3S}), 68.3 (C_{4S}),71.1 (C_{5S}), 71.2 (Cβ_{aThr}), 94.0 (C_{1S}), 156.8 (Cε_{Arg}), 170.8, 171.1, 172.0, 173.4, 173.7, 173.8, 174.0, 174.6, 176.7 (CO). 's' stands for sugar moiety.

Glycopeptide **2-Hnv***

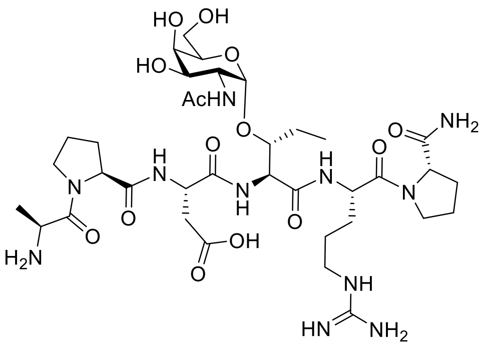

HRMS (ESI+) m/z: calcd. for $C_{36}H_{62}N_{11}O_{14}$ $[M+1H]^+$: 872.4472 found: 872.4446. Semipreparative HPLC on a Phenomenex Luna C18(2) column (10 µm, 250 mm x 21.2 mm), Rt = 12.7 min (Grad: water 0.1% TFA/acetonitrile (92:8) → (86:14), 14 min, λ = 212 nm).

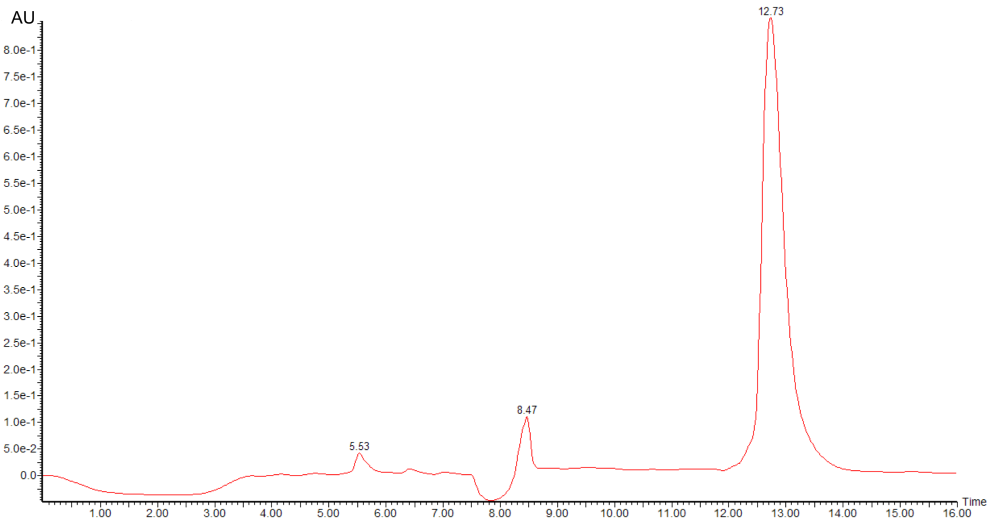

HPLC chromatogram of glycopeptide 2-Hnv*

^{1}H NMR (400 MHz, D_2O) δ (ppm): 0.87 (t, 3H, J = 7.4 Hz, Me_{Hnv}), 1.47 – 1.64 (m, 5H, Me_{Ala}), 1.69 – 2.07 (m, 12H, $NHCOC\underline{H}_{3GalNAc}$, $H\beta_{Pro7}$, $H\beta_{Pro11}$, $2H\gamma_{Pro7}$, $2H\gamma_{Pro11}$, $2H\beta_{Arg}$, $2H\gamma_{Arg}$), 2.28 – 2.39 (m, 2H, $H\beta_{Pro7}$, $H\beta_{Pro11}$), 2.84 – 3.06 (m, 2H, $2H\beta_{Asp}$), 3.21 – 3.26 (m, 2H, $2H\delta_{Arg}$), 3.62 – 3.68 (m, 2H, $H\delta_{Pro7}$, $H\delta_{Pro11}$), 3.71 – 3.78 (m, 4H, $H\delta_{Pro7}$, $H\delta_{Pro11}$, $2H_{6S}$), 3.86 (dd, 1H, J = 11.0, 3.0 Hz, H_{3S}), 3.98 (d, 1H, J = 2.6 Hz, H_{4S}), 4.05 (‘t’, 1H, J = 6.1 Hz, H_{5S}), 4.13 (dd, 1H, J = 11.0, 3.8 Hz, H_{2S}), 4.16 – 4.22 (m, 1H, $H\beta_{Hnv}$), 4.34 – 4.41 (m, 2H, $H\alpha_{Pro11}$, $H\alpha_{Ala}$), 4.48 – 4.52 (m, 1H, $H\alpha_{Pro7}$), 4.61 – 4.68 (m, 2H, $H\alpha_{Arg}$, $H\alpha_{Hnv}$), 4.85 – 4.92 (m, 2H, H_{1S}, $H\alpha_{Asp}$). ‘s’ stands for sugar moiety.

^{1}H NMR (400 MHz, D_2O/H_2O, 1:9, amide region) δ (ppm): 6.99 (s, 1H, $NH_{2\ Pro11}$), 7.21 – 7.23 (m, 1H, NH_{Arg}), 7.73 – 7.76 (m, 2H, N$\underline{H}$COCH$_3$, $NH_{2\ Pro11}$), 8.40 (d, 1H, J = 7.2 Hz, NH_{Arg}), 8.46 (d, 1H, J = 8.9 Hz, NH_{Hnv}), 8.72 (d, 1H, J = 7.2 Hz, NH_{Asp}).

^{13}C NMR (75 MHz, D_2O) δ (ppm): 9.0 (Me_{Hnv}), 15.04 (Me_{Ala}), 22.30 (NHCOC$\underline{H}_3$), 25.3 (CH_{2Hnv}), 24.2 ($C\gamma_{Arg}$), 24.6 ($C\gamma_{Pro7}$), 24.7 ($C\gamma_{Pro11}$), 27.4 ($C\beta_{Arg}$), 29.4 ($C\beta_{Pro6}$), 29.5 ($C\beta_{Pro2}$), 35.1 ($C\beta_{Asp}$), 40.5 ($C\delta_{Arg}$), 47.6 ($C\delta_{Pro7}$), 47.7 ($C\delta_{Pro11}$), 48.0 ($C\alpha_{Ala}$), 49.5 ($C\alpha_{Asp}$), 49.7 (C_{2S}), 51.2 ($C\alpha_{Arg}$), 53.9 ($C\alpha_{Hnv}$), 60.1 ($C\alpha_{Pro11}$), 60.2 ($C\alpha_{Pro7}$), 61.3 (C_{6S}), 68.2 (C_{3S}), 68.5 (C_{4S}), 71.5 (C_{5S}),79.7 ($C\beta_{Hnv}$), 98.3 (C_{1S}),156.7 ($C\varepsilon_{Arg}$), 169.2, 170.0, 171.5, 172.7, 173.3, 173.8, 174.1, 176.8 (CO). ‘s’ stands for sugar moiety.

Glycopeptide **1’-Thr**

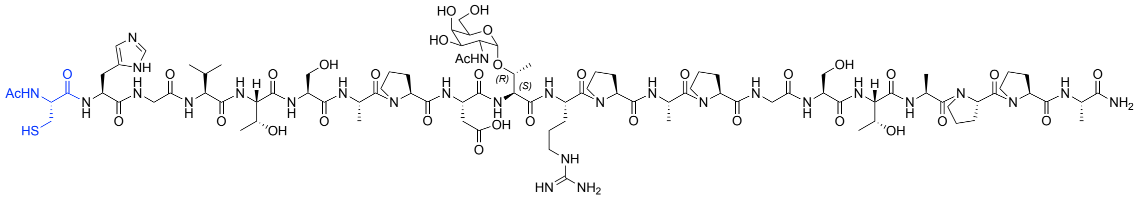

MALDI calcd. for $C_{93}H_{148}N_{28}O_{34}S$: 2233,0433 found: 2233.986. Semipreparative HPLC on a Phenomenex Luna C18(2) column (10 µm, 250 mm x 21.2 mm), Rt = 23.1 min (Grad: water 0.1% TFA/acetonitrile (95:5) → (81:19), 28 min, λ = 212 nm).

MALDI of compound 1’-Thr

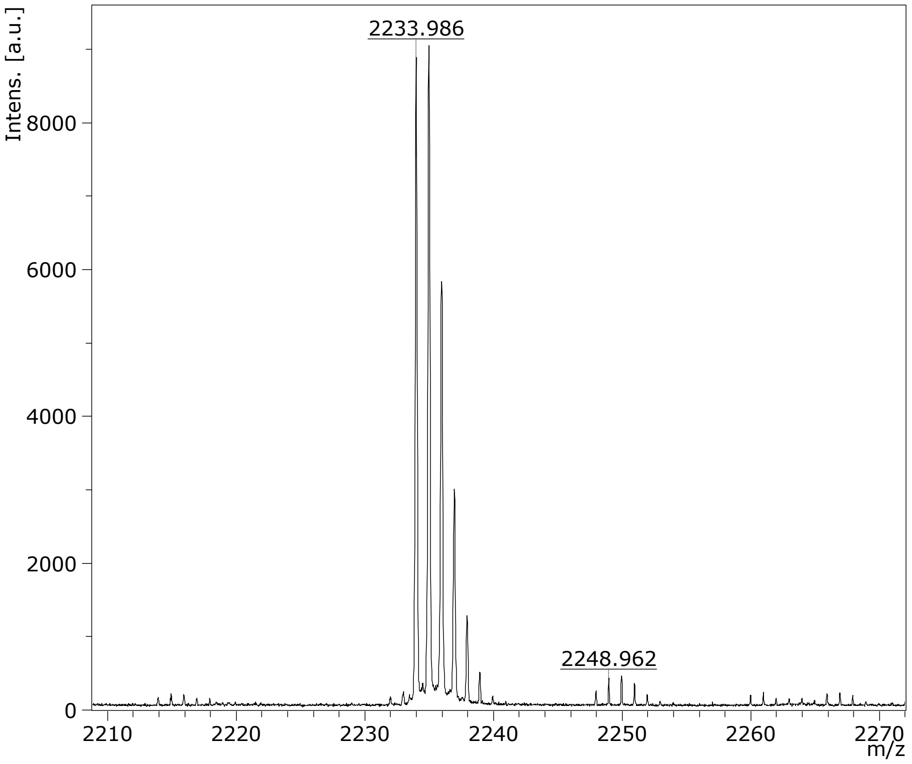

Glycopeptide **1'-Hnv***

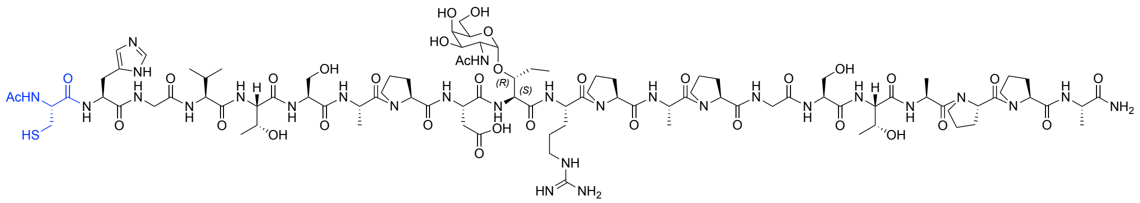

MALDI calcd. for $C_{94}H_{150}N_{28}O_{34}S$: 764.6821 found: 2247,0590 found: 2247.959. Semipreparative HPLC on a Phenomenex Luna C18(2) column (10 µm, 250 mm x 21.2 mm), Rt = 24.4 min (Grad: water 0.1% TFA/acetonitrile (95:5) → (81:19), 28 min, λ = 212 nm).

MALDI of compound 1'-Hnv*

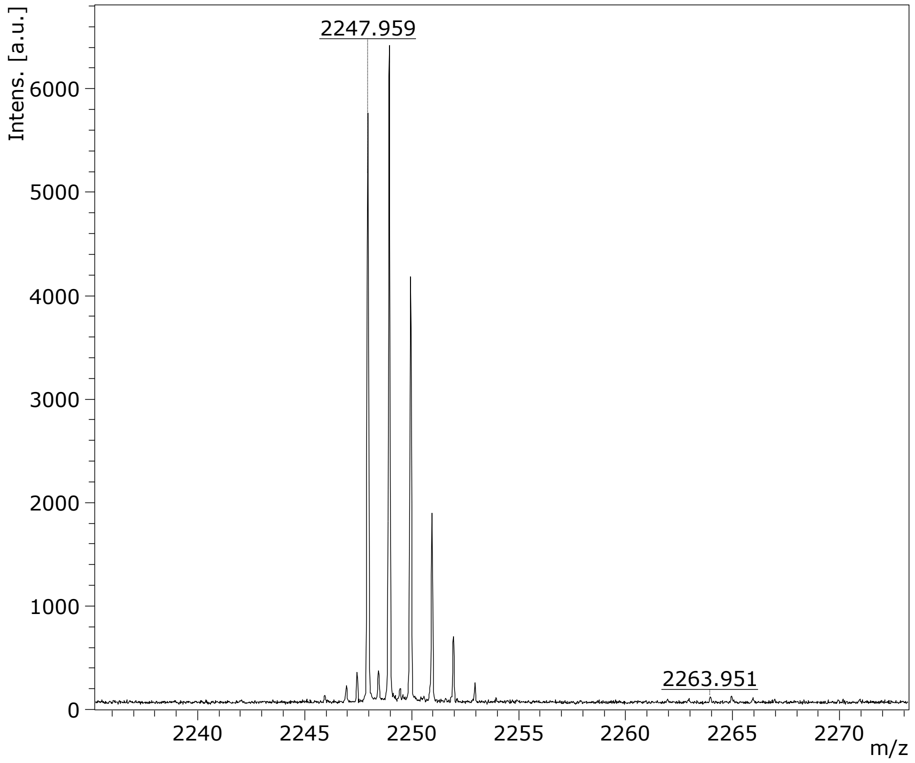

3. Biolayer Interferometry (BLI) assays

Binding assays were performed on an Octet Red Instrument (forteBIO). Ligand immobilization, binding reactions, regeneration, and washes were conducted in wells of black polypropylene 96-well microplates.

(Glyco)peptides (10 mg/mL) were immobilized on amine-reactive biosensors (AR2G biosensors) in 10 mM sodium acetate pH 5.5 buffer, using 1-ethyl-3-(3-dimethylaminopropyl)carbodiimide and *N*-hydroxysuccinimide as a coupling agent for 10 min at 1000 rpm at 25 ºC. The excess of reactive esters was then blocked with a solution of ethanolamine hydrochloride (1 M, pH 8.5), followed by regeneration (glycine pH 2.0 buffer) and washing.

Binding analyses were carried out at 25 ºC, 1000 rpm in 10 mM sodium phosphate buffer (pH 7.4) containing 150 mM NaCl, using different concentrations of scFv-SM3 antibody.[2] The surface was thoroughly washed with the running buffer without regeneration solution. Data were analyzed using Data Analysis (forteBIO) with Savitzky-Golay filtering. Binding was fitted to a 2:1 heterogeneous ligand model. Steady-state analysis was performed to obtain the binding constants (K_D, Fig. S1).

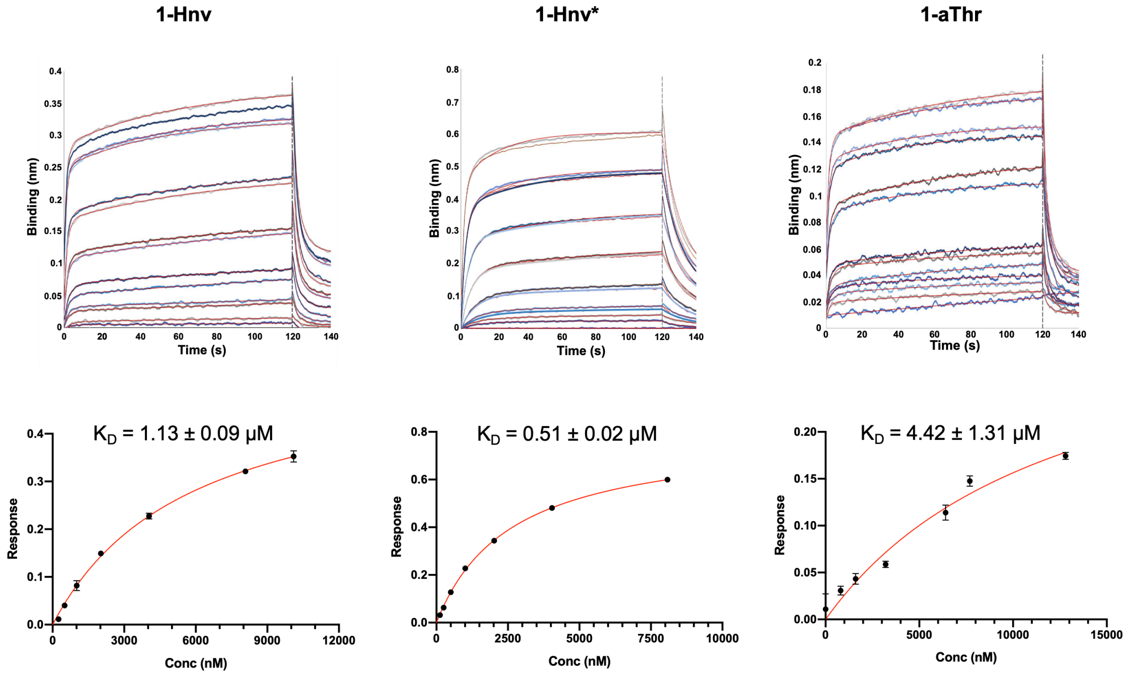

Fig. S1. BLI curves (upper panel) and fitting curves (lower panel) obtained for different (glyco)peptides studied in this work with scFv-SM3, together with the K_D constants derived from these experiments.

4. Surface plasmon resonance (SPR) assays

SPR experiments were performed with a Biacore X-100 apparatus (Biacore GE) using 25 mM PBS buffer with 0.005% tween as running buffer at temperatures of 7-35 °C. Flow cells (CM5 sensor chip; Biacore) were activated for 7 min by injecting 140 µL of of a 1:1 ratio of aqueous 50 mM *N*-hydroxysuccinimide (NHS):200 mM ethyl-3(3-dimethylamino)propylcarbodiimide (EDC). Commercially available SM3 antibody (from Abcam) was immobilized on the activated gold chip in flow cell 2 by injection of a 100 µg/mL protein solution diluted with 10 mM sodium acetate buffer with a flow rate of 10 µL/min for 7 min, followed by an injection of 130 µL ethanolamine to block any remaining activated groups on the surface. The level of immobilization reached was about 3000 RUs. Flow cell 1, used as a reference, was blocked with ethanolamine under the same conditions as flow cell 2 without immobilization of protein. Affinity experiments were conducted using a series of different concentrations of each epitope in the range of 0.025–5 mM with a flow rate of 30 µL/min for 60 s. Each injection was followed by a 100 s injection of running buffer (dissociation phase). No regeneration steps were performed between injections. Response data were collected in real-time and analyzed with the Biacore® X-100. Evaluation software and plotted as response shift versus analyte concentration.

SPR curves obtained for glycopeptides **2-Thr*** and **2-Hnv*** at 25 ºC are shown in Fig. S2. K_D values assessed at different temperatures are shown in Table S1.

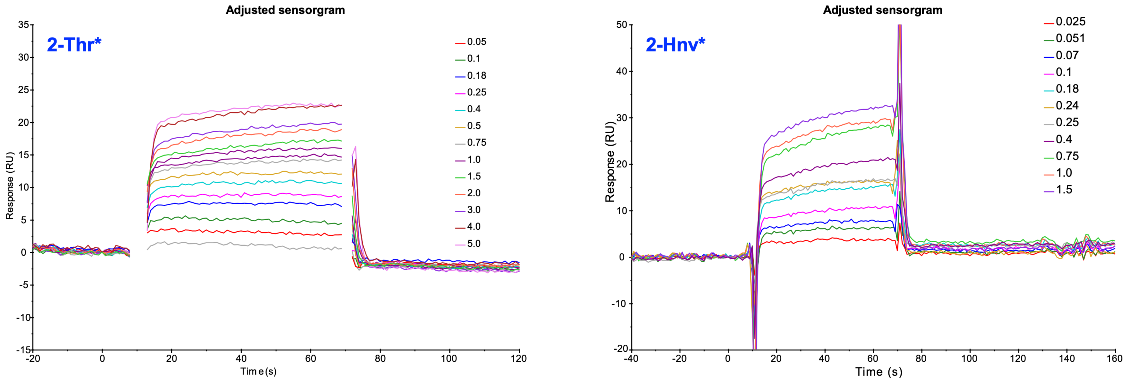

Fig. S2. SPR sensorgrams obtained for the binding of derivatives **2-Thr*** and **2-Hnv*** towards the commercially available SM3 antibody (Abcam) at 25 ºC. Concentrations are given in mM. RU stands for response units.

Table S1. K_D values of glycopeptides **2-Thr*** and **2-Hnv*** towards the commercially available SM3 antibody (Abcam) were determined by SPR at different temperatures.

**2-Thr***			
T (ºC)	*K_D (M)*	*R_{max} (RU)*	*$Chi^2 (RU^2)$*
7	1.967E-4	13.39	0.266
15	2.721E-4	16.54	0.331
25	6.189E-4	23.05	0.271
37	0.001377	25.58	0.112
**2-Hnv***			
T (ºC)	*K_D (M)*	*R_{max} (RU)*	*$Chi^2 (RU^2)$*
10	9.427E-5	14.32	0.0381
15	1.264E-5	27.81	0.634
25	3.036E-4	37.55	0.700
37	0.001989	52.69	1.687

K_D values, together with $R \cdot ln(K_D)$ for glycopeptides **2-Thr*** and **2-Hnv*** with SM3 antibody at different temperatures, are shown in Table S2. These values were used to estimate the thermodynamic parameters (ΔH and ΔS) by using the Van't Hoff equation:

$$\mathrm{R} \ln K_D = \Delta H \cdot \frac{1}{T} - \Delta S$$

Table S2. K_D and $R \cdot ln(K_D)$ values for glycopeptides **2-Thr*** and **2-Hnv*** with SM3 antibody at different temperatures are shown.

	**2-Thr***		**2-Hnv***	
T (ºC)	*K_D (M)*	*$R \cdot ln (K_D)$*	*K_D (M)*	*$R \cdot ln (K_D)$*
37	0.001377	-54.77447823	0.001989	-51.776306
25	0.0006189	-61.42371772	0.0003036	-67.422731
15	0.0002721	-68.25633515	0.0001264	-74.716716
10	-	-	0.00009427	-77.065355
7	0.0001967	-70.95429751	-	-

According to Van't Hoff equation, by plotting $R \cdot lnK_D$ against 1/T, ΔH and ΔS can be estimated as the slope and Y-axis intercept of the resulting straight line (Fig. S3 and Table S3).

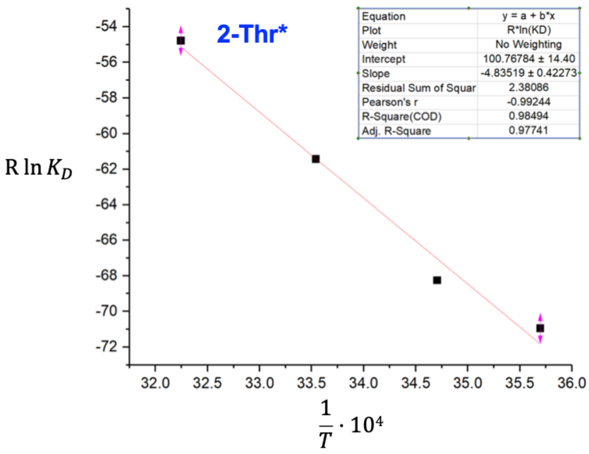

ΔH = -(11.56 ± 1.01) kcal/mol

ΔS = -(0.024 ± 0.003) kcal/mol·K

ΔH = -(4.83519 ± 0.42273)·10^4 J/mol

$-\Delta S$ = 100.8 ± 14.4 J/mol·K

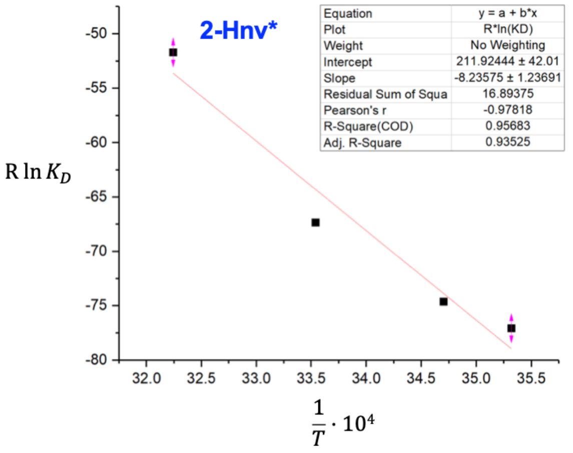

ΔH = -(19.68 ± 2.96) kcal/mol

ΔS = -(0.051 ± 0.010) kcal/mol·K

ΔH = -(8.23575 ± 1.23691)·10^4 J/mol

$-\Delta S$ = 211.9 ± 42.0 J/mol·K

Fig. S3. Thermodynamic parameters of the glycopeptides **2-Thr*** and **2-Hnv*** with SM3 antibody derived from the SPR experiments performed at different temperatures.

Table S3. Thermodynamic parameters **2-Thr*** and **2-Hnv*** at 25 ºC. All values are in Kcal/mol.

	**2-Thr***	**2-Hnv***
ΔH	– (11.55±1.01)	– (19.68±2.96)
$-298.15 \cdot \Delta S$	7.18±1.03	15.10±2.99
$\Delta G\ (T = 298.15\ K)$	– 4.37	– 4.57

5. Microarray studies of 1-Thr* and 1-Hnv* with SM3 and VU-3C6 anti-MUC1 antibodies.

Microarray slides called "Antibody chips" were obtained from Sumitomo Bakelite Co., Ltd. (Tokyo, Japan). The slide size is 75-mm long, 25 mm wide, and 1 mm thick. Hybridization covers (60 × 25 × 0.7 mm) were also obtained from Sumitomo Bakelite Co., Ltd. Anti-MUC1 mouse mAb VU-3C6 (0.86 mg/mL) was purchased from Exalpha Biological Inc. (Massachusetts, United States), and FluoroLinkTM CyTM3-labeled goat anti-mouse IgG was from Amersham Biosciences (Buckinghamshire, UK).

Microarray printing. We selected plastic "Antibody chip" (Sumitomo Bakelite, Japan) due to the non-fouling surface and selective covalent immobilization to the *N*-terminal amino group of the MUC1 (glyco)peptides.

The printing on slides of (glyco)peptides **1-Thr**, **1-Thr***, **1-Hnv,** and **1-Hnv*** was done following the manufacturer's instructions and using the microarray slides' buffers kit. (Glyco)peptides were spotted by MicroSys 5100 (Cartesian Technologies, CA, USA) with a 0.6 mm pitch using a Filgen solid spin (200 µm pin diameter). Each compound was printed in quadruplicate with a 0.3 mm distance between spots of the same compound and a 0.6 mm gap among different compounds (Fig. S4, left panel). Each (glyco)peptide was printed at five different concentrations from 250 µM to 15.6 µM (Fig. S4, right panel). Cy3 labeled BSA protein (25 µg/mL) was used as a grid. Spotting conditions were 23 °C and 60% of humidity. After printing, slides were incubated overnight in dry conditions. Next, non-reacted groups were inactivated by blocking buffer at 37 °C for 1 h under slow agitation. Finally, we rinsed the slides by washing buffer (3 × 5 min), dried them by centrifugation and then used them for further binding assay of monoclonal antibodies (mAb).

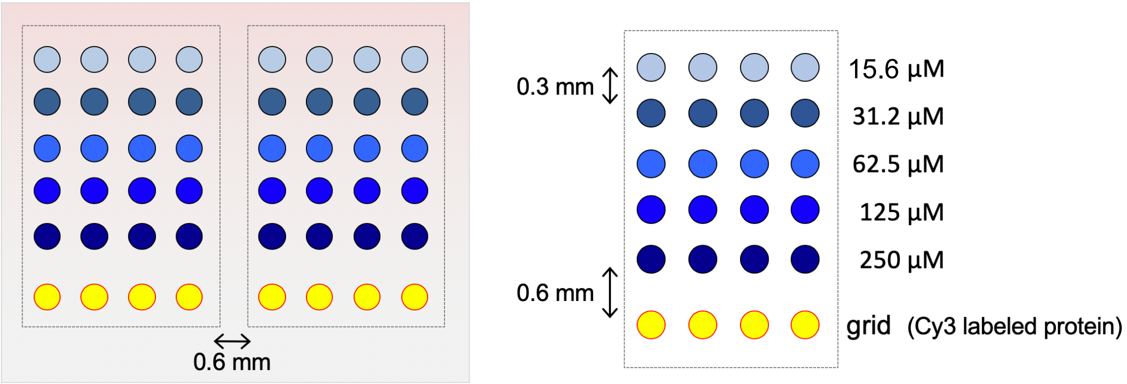

Fig. S4. Microarray glycopeptides slides, schematic microarray printing on chamber slide (left panel), and printing pattern of each compound group (right panel).

Microarray mAb binding assay. The following buffers and solutions were used in this section: Buffer for the solution of mAb: 50 mM Tris-HCl, 100 mM NaCl, 1 mM $CaCl_2$, $MnCl_2$, $MgCl_2$, 0.05% Tween-20, 0.1% BSA, pH 7.4. Washing buffer: 50 mM Tris-HCl, 100 mM NaCl, 1 mM $CaCl_2$, $MnCl_2$, $MgCl_2$, 0.05% Triton X-100, pH 7.4. For the mAb incubation, 20 µL of mAb solution in buffer (mAb concentration: 50.0 µg/mL) was carefully added to each chamber of slides and kept at rt for 2 h under humid conditions. Next, slides were washed with washing buffer (3 × 2 min) and dried up by centrifugation. For the binding analysis, secondary Ab (Cy3-labeled Ab) was diluted to 1 µg/mL in buffer and infused between hybridization covers and slides. After standing at rt for 1 h at dark, slides were washed by: (1) washing buffer (3 × 2 min) and centrifugal drying; (2) followed by washing buffer (2 × 2 min), washing with H_2O (2 × 2 min) and dried up by centrifugation. To store the slides, they were degassed under vacuum and kept at 4 °C. Slides were subjected to fluorescent image scanning on a Tryphoon Trio Plus instrument (GE Healthcare). Array Vision software was employed for the quantification of the fluorescence of each spot. The median value of relative fluorescence intensity (RFU) was used; spot intensities were determined by subtracting the average pixel intensity from the median pixel intensity of the local background within the spots. The fluorescence of each spot is shown as the average of four replicate spots used to construct histograms showing the antibody-binding profile. The Grubbs method was used as a statistical analysis to discriminate the outliers. Error bars show the standard deviation for each peptide–mAb interaction (Fig. S5).

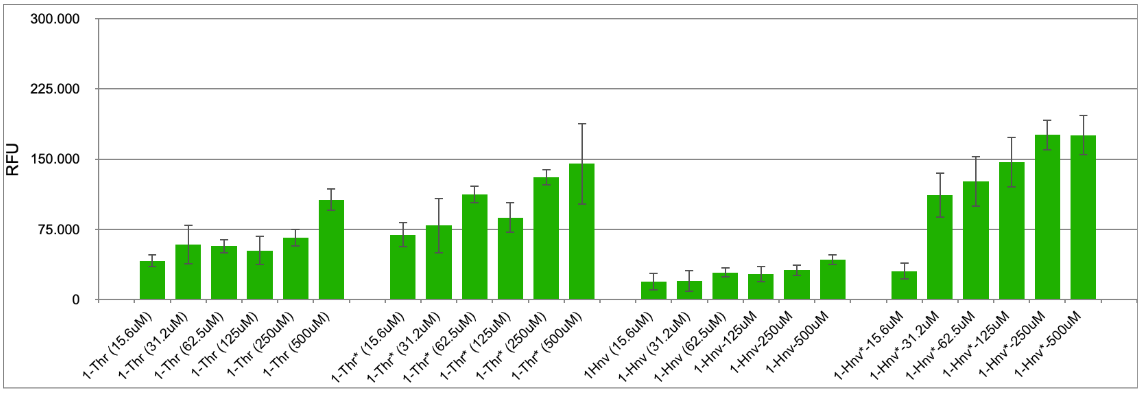

Fig. S5. Binding studies of (glyco)peptides **1-Thr**, **1-Thr***, **1-Hnv,** and **1-Hnv*** with the commercially available antibody anti-MUC1 mAb VU-3C6, using a microarray platform. Compounds were printed onto an "Antibody chip" microarray in quadruplicate. Relative fluorescence units (RFU) due to the binding of the Cy3-labeled secondary antibody were measured and represented as mean values in a bar chart.

6. STD-NMR studies of glycopeptide 2-Hnv* with anti-MUC1 antibody VU-3C6

The interactions of glycopeptide **2-Hnv*** in the presence of the anti-MUC1 antibody VU-3C6 (ref. 3) were also studied by STD-NMR using our previous protocol.[4] All the NMR experiments were recorded on a Bruker Avance III 600 MHz spectrometer equipped with a 5-mm inverse detection triple-resonance cryogenic probe head with z-gradients. The glycopeptide **2-Hnv*** was completely assigned through standard 2D-TOCSY (30 and 80 ms mixing time) and 2D-NOESY (400 ms mixing time) at 278 K. The glycopeptide was characterized in a buffer containing 20 mM PBS, 20 mM NaCl, 0.09% NaN_3 buffer, pH 7.1 in H_2O/D_2O (90:10) with a concentration of 1 mM. The resonance of 2,2,3,3-tetradeutero-3-trimethylsilylpropionic acid (TSP) was used as a chemical shift reference in the ^{1}H-NMR experiments (δ TSP = 0 ppm).

The STD-NMR experiment was acquired using a 40:1 molar ratio of 8 µM VU-3C6 (GeneTex, Inc.) and 320 µM of **2-Hnv*** in a 100% deuterated buffer containing 20 mM PBS, 20 mM NaCl, 0.09% NaN3, pD 7.1 at 310 K. The STD-NMR spectra (stddiffesgp pulse sequence from Bruker pulse program library) were acquired with 4160 scans in a matrix with 64k data points in t2 and in a spectral window of 12335.5 Hz centered at 2822.9 Hz. The selective saturation of the protein resonances (on resonance) was performed by irradiating at -0.5 ppm, using a series of 40 Eburp2.1000-shaped 90º pulses (50 ms, 1 ms delay between pulses) for a total saturation time of 2 s. For the reference spectrum (off resonance), the samples were irradiated at 100 ppm. A peptide control experiment was performed for **2-Hnv*** in absence of VU-3C6, where residual STD signals for the methyl groups of Hnv, Ala and GalNAc were observed, as well as for H6s of GalNAc. This result was considered and subtracted when analyzing the STD experiment, to obtain an accurate epitope mapping of the interaction. The STD spectrum (ISTD) was obtained by subtracting the on-resonance spectrum (Ion) to the off-resonance spectrum (Ioff). The % of STD (ISTD/Ioff x 100) were estimated by comparing the intensity of the signals in the STD spectrum (ISTD) with the signal intensities of the reference spectrum (Ioff). To determine the STD-derived epitope map the relative % of STD were calculated by setting to 100% the STD signal of the proton with the highest STD intensity and calculating the others accordingly.

Some protons were not able to be assessed with accuracy due to the use of water suppression or low signal/noise ratio and display a blue circle in the STD-derived epitope maps. Moreover, the resonances overlapped on the ^{1}H-NMR spectrum were considered in STD estimation and are labelled as '*' (Fig. S6).

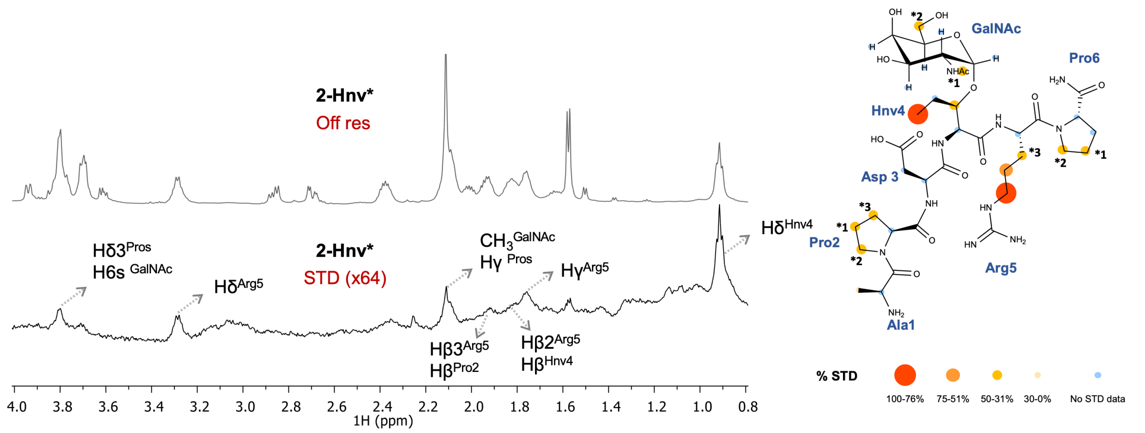

Fig. S6. STD-NMR analysis of the glycopeptide **2-Hnv*** in presence of VU-3C6 in a ligand:protein 40:1 molar ratio obtained at 600 MHz and 310 K. Left panel: The off-resonance spectrum (Off res) is displayed in dark grey, while the STD spectrum is displayed in black. Right panel: STD-derived epitope mapping of glycopeptide **2-Hnv*** in presence of VU-3C6. Protons that could not be accurately analyzed are displayed in blue. The proton resonances that overlap in the spectrum are identified in the figure with the symbol '*'.

7. Conformational analysis of glycopeptides 2-Thr* and 2-Hnv* in water

7.1. 2D ROESY experiments.

ROESY experiments were recorded on a Bruker Avance 400 spectrometer at 298 K and pH 6.5 in H_2O/D_2O (9:1). The experiments were conducted using phase-sensitive ge-2D ROESY with WATERGATE for H_2O/D_2O (9:1) spectra. ROESY intensities were normalized to the diagonal peak at zero mixing time. Distances involving NH protons were semi-quantitatively determined by integrating the volume of the corresponding cross-peaks. The number of scans used was 32, and the mixing time was 500 ms (Fig. S7)

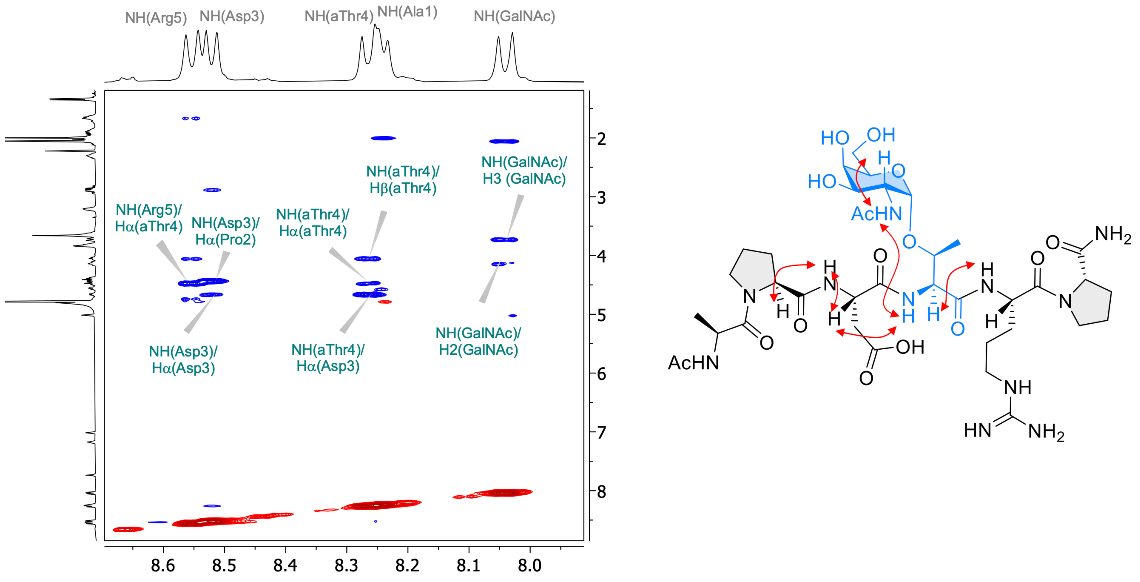

Fig. S7. Sections of the 500 ms 2D ROESY spectrum (400 MHz) in H_2O/D_2O (9:1) at 298 K and pH = 6.5 of glycopeptide **2-aThr*** that show the amide region. Diagonal peaks are in red. ROE contacts are represented as blue cross-peaks. The second set of signals (in small relative intensity) observed in the spectra correspond to the *cis* configuration of the amide bond of proline residues.[5] A schematic representation of **2-Hnv***, showing the most relevant ROE contacts is also presented in this figure.

*Note: Compound **2-aThr*** was synthesized as an N-acetyl derivative (rather than a glycopeptide with a free N-terminal Ala residue). Subsequently, all studies reported in this paper were performed with this compound. It is important to point out that our previous studies on this type of glycopeptides indicate that the conformations observed in solution remain substantially unaltered, irrespective of the presence or absence of the N-terminal acetyl group. This conclusion is confirmed by comparing our results with those of the natural glycopeptide with acetyl group[6] and free N-terminal amino group.[7]*

7.2. Circular dichroism (CD).

CD spectra of (glyco)peptides **2-Thr**, **2-Thr*,** and **2-Hnv*** were measured at 100 μM in sodium phosphate buffer (pH 7.5, 25 mM). All curves were measured at 20 ºC in 1 mm path length quartz cells (Fig. S8).

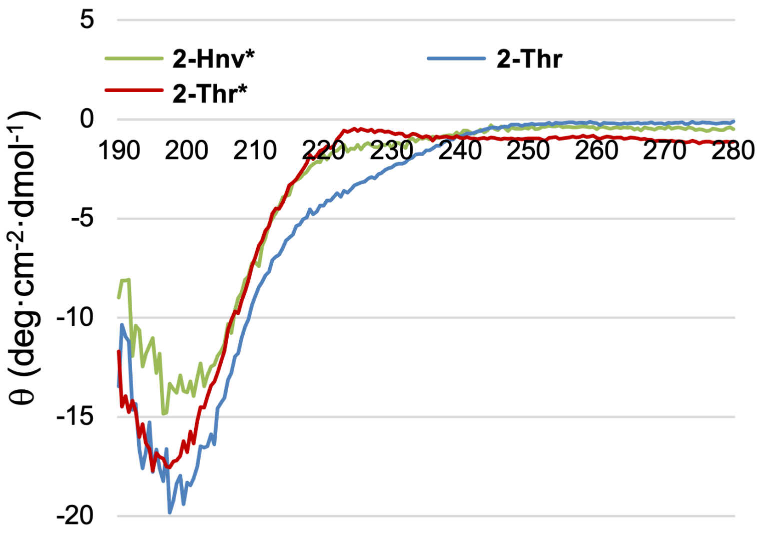

Fig. S8. Circular dichroism (CD) spectra of compounds **2-Thr**, **2-Thr***, and **2-Hnv*** (100 μM in sodium phosphate buffer, pH 7.5, 25 mM, 20 °C).

7.3 Molecular dynamics (MD) simulations of glycopeptides 2-aThr* and 2-Hnv* in water with time-averaged restraints (MD-tar or experiment-guided MD simulations).

The simulations were carried out with AMBER 18 package[8] implemented with ff14SB,[9] GAFF,[10] and GLYCAM06j[11] force fields. The parameters and charges for the unnatural amino acids were generated with the antechamber module of AMBER, using GAFF force field and AM1-BCC method[12] for charges. Each molecule was then immersed in a water box with a 10 Å buffer of TIP3P water molecules.[13] The system was neutralized by adding explicit counter ions (Cl^-). Before MD-tar productive simulations, we performed an equilibration protocol consisting of an initial minimization of the water box of 5000 steps, followed by a 2500-step minimization of the whole system. Then, the water box was heated at constant volume until 300 K, using a time constant for the heat bath coupling of 1 ps. The equilibration finished with 200 ps of MD simulation without restraints, at a constant pressure of 1 bar, and turning on the Langevin temperature scaling with a collision frequency of 1 ps. Non-bonded interactions were cut-off at 8.0 Å and updated every 25 steps. Periodic boundary conditions and the Particle Mesh Ewald method[14]

were turned on in every step of the equilibration protocol to evaluate the long-range electrostatic forces, using a grid spacing of approximately 1 Å. The ROESY-derived distances (Table S4) were imposed as time-averaged constraints, applying an r^{-6} averaging. The equilibrium distance range was set to r_{exp} −0.2 Å ≤ r_{exp} ≤ 0.2 Å. Trajectories were run at 298 K, with a decay constant of 20000 ps and a time step of 1 fs. The force constants r_{k2} and r_{k3} used in each case were 10 kcal·mol^{-1}·Å^{-2}. The overall simulation length was 200 ns. The coordinates were saved each 1 ps. Convergence within the equilibrium distance range was obtained in the simulations (Fig. S10-S12).

Table S4. Comparison of the experimental and MD-tar derived distances and ^3J coupling constants for glycopeptides **2-aThr*** and **2-Hnv***. The experimental-derived distances were semi-quantitatively determined by integrating the volume of the corresponding cross-peaks in the ROESY spectra (see Fig. S6 and Fig. 3a in the main text). All the distances are given in Å.

**Glycopeptide 2-aThr***		
**Distances (Å)**	**Experimental values**	**MD-tar**
NH(Arg5)-Hα(aThr4)	2.2	2.2
NH(Asp3)-Hα(Pro2)	2.1	2.2
NH(Asp3)-Hα(Asp3)	2.8	2.9
NH(aThr)-Hα(Asp3)	2.2	2.3
NH(aThr)-Hα(aThr4)	2.8	2.9

**Glycopeptide 2-Hnv***		
**Distances (Å)**	**Experimental values**	**MD-tar**
NH(Asp3)- Hα(Pro2)	2.2	2.3
NH(Hnv4)-NH(Asp3)	2.2	2.3
NH(Arg5)-Hα(Hnv4)	2.2	2.3
NH(Hnv4)-NH(GalNAc)	3.0	2.9
**coupling constants (Hz)**		
$^3J_{H5,H6R}$[15]	6.1	5.3
$^3J_{H5,H6S}$[15]	6.1	5.0

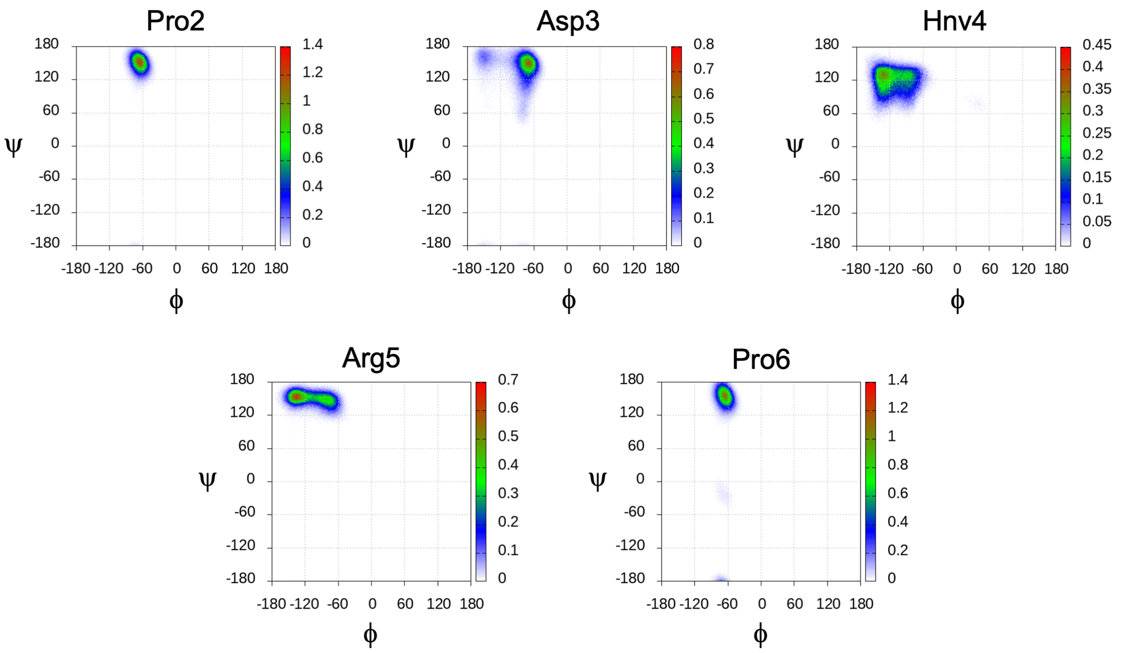

Fig. S9. ϕ/ψ distributions for the peptide backbone of glycopeptide **2-Hnv*** obtained through 200 ns experiment-guided MD simulations in explicit water. $\phi = C_{i-1}–N_i–C\alpha_i–C_i$, $\psi = N_i–C\alpha_i–C_i–N_{i+1}$.

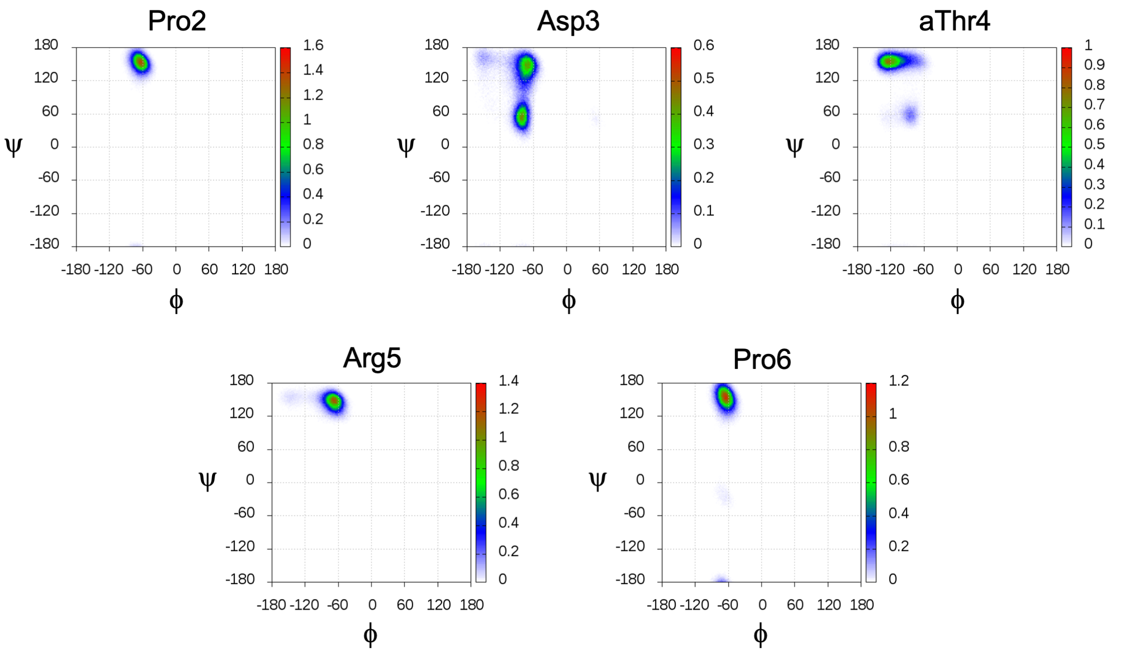

Fig. S10. ϕ/ψ distributions for the peptide backbone of glycopeptide **2-aThr*** obtained through 200 ns experiment-guided MD simulations in explicit water. $\phi = C_{i-1}–N_i–C\alpha_i–C_i$, $\psi = N_i–C\alpha_i–C_i–N_{i+1}$.

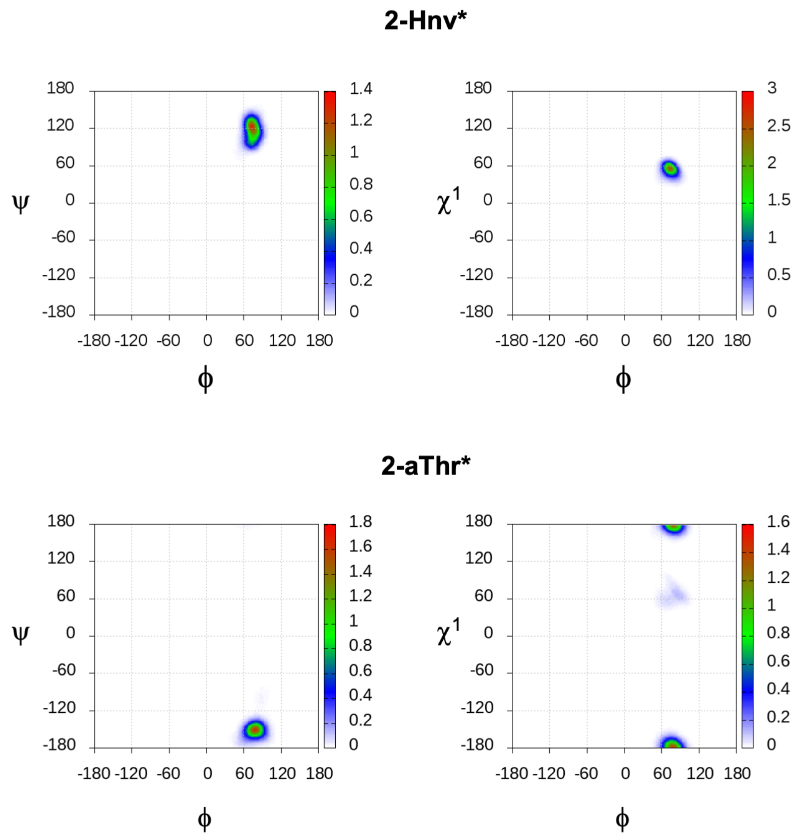

Fig. S11. φ/ψ distributions for the glycosidic linkage of glycopeptides **2-Hnv*** and **2-aThr*** obtained through 200 ns experiment-guided MD simulations in explicit water, together with the conformation of the sidechain. φ = O5–C1–O1–Cβ, ψ = C1–O1–Cβ–Cα, χ^1 = O1–Cβ–Cα–N.

8. Crystal structure of glycopeptide 2-Hnv* complexed with scFv-SM3.

8.1. Crystallization. Expression and purification of scFv-SM3 have been described previously by us.[2] Crystals were grown by sitting drop diffusion at 18 ºC. The drops were prepared by mixing 0.5 µL of protein solution containing 15 mg/mL of scFv-SM3 and 10 mM of glycopeptide **2-Hnv*** with 0.5 µL of the mother liquor. Crystals of scFv-SM3 with **2-Hnv*** were grown in 20% PEG 3350, 0.2 M disodium hydrogen phosphate. The crystals were cryoprotected in mother liquor containing 15% ethylene glycol and frozen in a nitrogen gas stream cooled to 100 K.

8.2. Structure determination and refinement. The data were processed and scaled using the XDS package[16] and CCP4 software.[17] Relevant statistics are given in Table S5 (see also Fig. 4 in the main text). The crystal structures were solved by molecular replacement with Phaser[18] and using the PDB entry 1SM3 as the template. Initial phases

were further improved by cycles of manual model building in Coot63 and refinement with REFMAC5.[19] The final models were validated with PROCHECK.[20] Coordinates and structure factors have been deposited in the Worldwide Protein Data Bank (wwPDB). PDB entry: 8AXH.

Table S5. Data collection and refinement statistics. Values in parentheses refer to the highest resolution shell. Ramachandran plot statistics were determined with PROCHECK.[20]

	2-Hnv*/scFv-SM3
Space group	$P2_12_12_1$
Wavelength (Å)	0.97
Resolution (Å)	20.00-1.85 (1.95-1.85)
Cell dimensions (Å)	*a* = 35.50<br>*b* = 68.49<br>*c* = 90.92
Unique reflections	17900
Completeness	91.0 (77.9)
R_{pim}	0.040 (0.340)
Mn(I) half-set correlation CC(1/2)	0.999 (0.768)
$I/\sigma(I)$	15.8 (2.2)
Redundancy	8.5 (8.3)
R_{work} / R_{free}	0.167/0.206
RMSD from ideal geometry, bonds (Å)	0.0087
RMSD from ideal geometry, angles (°)	1.5464
<*B*> protein (Å^2)	24.46
<*B*> glycopeptide (Å^2)	33.50
<*B*> solvent (Å^2)	33.97
<*B*> ethylenglycol (Å^2)	44.77
Ramachandran plot:<br>Most favoured (%)<br>Additionally allowed (%)<br>Disallowed (%)	 95.43<br>2.74<br>1.83
PDB ID	8AXH

9. MD simulations of glycopeptides 2-Hnv*, 2-aThr*, and 2-MeSer* in complex with Fab-SM3.

The simulations were carried out with AMBER 18 package[8] implemented with ff14SB,[9] GAFF[10], and GLYCAM06j[11] force fields. The parameters and charges for the unnatural amino acids were generated with the antechamber module of AMBER, using GAFF force field and AM1-BCC method[12] for charges. The crystal structure of the Fab structure of SM3 in complex with a peptide (PDB entry: 1SM3)[21] was used in these studies. Each molecule was then immersed in a water box with a 10 Å buffer of TIP3P water molecules.[13] The system was neutralized by adding explicit counter ions. A two-stage geometry optimization approach was performed. The first stage minimizes only the positions of solvent molecules, and the second stage is an unrestrained minimization of all the atoms in the simulation cell. The systems were then gently heated by incrementing the temperature from 0 to 300 K under the constant pressure of 1 atm and periodic boundary conditions. Harmonic restraints of 30 $kcal \cdot mol^{-1}$ were applied to the solute, and the Andersen temperature-coupling scheme was used to control and equalize the temperature. The time step was kept at 1 fs during the heating stages, allowing potential inhomogeneities to self-adjust. Long-range electrostatic effects were modeled using the particle-mesh-Ewald method.[14] An 8 Å cut-off was applied to Lennard-Jones interactions. Each system was equilibrated for 2 ns with a 2-fs time step at a constant volume and temperature of 300 K. Production trajectories were then run for additional 0.5 μs under the same simulation conditions (Fig. S12).

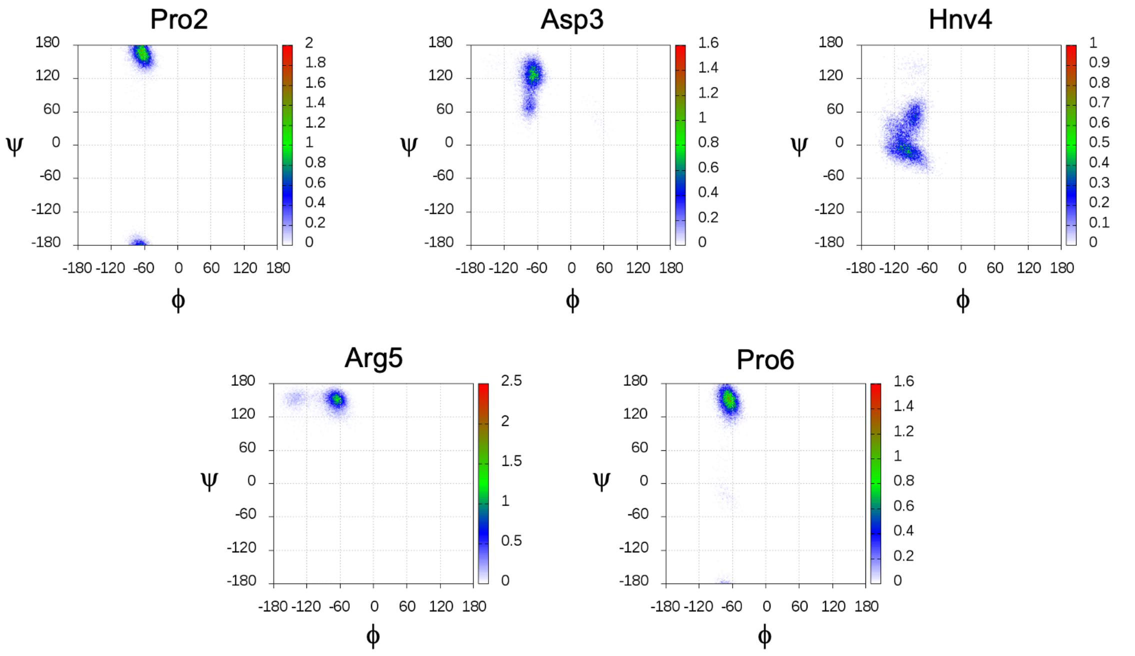

Fig. S12. ϕ/ψ distributions for the peptide backbone of glycopeptide **2-Hnv*** in complex with Fab-SM3 obtained through 0.5 μs experiment-guided MD simulations in explicit water. $\phi = C_{i-1}–N_i–C\alpha_i–C_i$, $\psi = N_i–C\alpha_i–C_i–N_{i+1}$.

10. Characterization of nanoparticles

10.1. Preparation and characterization of gold nanoparticles (AuNPs). The conjugation of **1’-Hnv*** and **1’-Thr*** with AuNPs and the quantification of the peptide loading on the nanoparticles was achieved following our previous reported protocol,[22] as briefly outlined in section 10.2. The copies of peptides per AuNP was estimated to be 200 ± 60.

10.2. Quantification of peptide loading on AuNPs

The average number of peptides per nanoparticle was determined via *amino acid analysis* using the *O*-phtalaldehyde (OPA) pre-column derivatization method according to an experimental protocol previously reported.[23] Shortly:

<u>Solutions needed:</u>

1. Dissolve 50 mg OPA in 1 mL HPLC-grade methanol.
2. Prepare 200 mM potassium tetraborate buffer pH 9.4 (from boric acid and KOH).
3. Prepare 500 μL of 5× OPA reagent: mix 50 μL OPA solution, 6 μL mercaptopropionic acid (MPA) and 444 μL borate buffer.
4. Prepare 1× OPA working solution: dilute 1 part of 5 × OPA reagent with 4 parts of borate buffer.

Notes: The molar ratio OPA/MPA is 1:3.6; 5 × OPA reagent can be stored protected from light at +4 °C for 1 week; 1× OPA working solution must be prepared just before analysis and kept on ice and protected from light.

5. Prepare the calibration solutions: Amino acid standard mix (AAS18 Sigma-Aldrich) and (2*S*,3*R*)-3-hydroxynorvaline (200 μM in 0.1 M HCl) as the internal standard; concentration of the calibration solutions in the interval 0.5 – 15.0 μM (+ 5.0 μM (2*S*,3*R*)-3-hydroxynorvaline) in 0.1 M HCl.

Microwave-assisted sample hydrolysis:

Typically, 20 μL of AuNPs (≥ 160 nM) and 20 μL of 25 μM (2*S*,3*R*)-3-hydroxynorvaline solution in 0.1 M HCl (internal standard) were placed into the cleaned Pyrex glass vials together with 700 μL of 6 M HCl and hydrolyzed for 20 min. at 175°C using a pressurized microwave acid digestion system from CEM (Discover SP-D).

Sample preparation procedure and analysis:

After hydrolysis, the samples were dried and the dry residues resuspended in 100 μL of 200 mM borate buffer pH 9.4 (vigorously vortexed for 15 min, sonicated for 15 min and then centrifuged at 22000 ×*g*, 20 °C, for 30 min). These reconstituted hydrolysates are directly processed with the OPA reagent.

1. Equal amounts of 1× OPA working solution and sample hydrolysate are quickly mixed and 20 μL of this mixture injected for HPLC analysis (90 sec after addition of the OPA reagent).
2. HPLC conditions: Zorbax Extend C18 column, 3.5 μm, 100 × 4.6 mm, 40°C, detection at 338 nm. Mobile phase A: (10 mM Na_2HPO_4, 10 mM $Na_2B_4O_7$) adjusted to pH 8.2 with HCl, mobile phase B was ACN/MeOH/H_2O 45:45:10 (v/v/v). Flow rate: 1.5 mL/min. Gradient: 1 min at 2% B then up to 58% B in 17.5 min, followed by 100% B (total analysis time 24 min). Re-equilibration at 2% B for at least 5 min before starting a new analysis. Quantification in terms of concentration of single amino acid (and hence of peptide) in the sample was carried out averaging the single values obtained for Val, Ala, Thr, Ser, Asp, considering the number of each of these amino acids in the peptide sequence.

Calculations:

Peptide concentration in a sample (determined as explained above) is divided by the concentration of nanoparticles in the sample affording the average number of peptides/nanoparticle.

10.3. Dynamic light scattering (DLS) and ζ-Potential measurements. The hydrodynamic radius of AuNPs was determined by dynamic light scattering. AuNPs were suspended in 10 mM $NaHCO_3$. ζ-potentials were measured in the same buffer. The values are reported in Table S6.

Table S6. Physicochemical characterization of AuNPs used in this work (Au-core diameter 13.0 ± 1.0 nm, TEM).[a]

**AuNPs**	**DLS**[b]		**ζ-Potential**[b]
	Ø (nm)[c]	PDI	(mV)
AuNP-**1'-Thr***	35.3 ± 2.8	0.216	-21 ± 4
AuNP-**1'-Hnv***	35.4 ± 3.5	0.222	-19 ± 5

[a]Reported values are averages of 8 nanoparticle preparations ± s.d. [b]Measurements in 10 mM $NaHCO_3$. [c]Derived from the intensity distribution.

10.4. Gel electrophoresis. Gel electrophoresis was carried out on 0.6% agarose gel using sodium boric acid (SB) buffer pH 8.5, for approx. 2 h at 70 V. Nanoparticle samples were diluted with loading buffer (1:1 SB buffer/glycerol) to 25 nM AuNPs before loading on the gel (~10 µL/lane). (Figure S13a)

10.5. UV/Vis measurements. They were carried out using a TECAN Infinite M200 Pro plate reader. The concentration of gold was determined via inductively coupled plasma-optical emission spectrometry (Agilent 720 ICP-OES, Figure S13b).

10.6. Preparation of peptide BSA conjugates for ELISA plate coating. Bovine serum albumin (BSA, A0281 Sigma Aldrich) was first derivatized using the $SM(PEG)_2$ amine-to-sulfhydryl heterobifunctional linker followed by conjugation of peptides **1'-Thr*** or **1'-Hnv***. Shortly, SM(PEG)2 (5×10^{-3} mmol in 100 µL dry acetonitrile, final conc. 5 mM in the reaction mixture) was added to an aqueous solution of BSA in phosphate buffer at pH 8.2 (final reaction volume 1.0 mL, BSA 30 µM, buffer 30 mM). The reaction mixture was incubated at 1 ºC for 3 h. Excess linker was then removed by ultrafiltration on Microcon-30kDa Centrifugal Filter Unit with Ultracel-30 membrane (washing with 3 × 500 µL 20 mM phosphate buffer pH 7.0) All cleaning steps were performed on ice, in a 4 ºC-cooled centrifuge, and with ice-cold wash solutions. Purified linker-functionalized BSA was taken up in 1.0 mL 20 mM phosphate buffer pH 7.0 at a concentration of 1.4 mg/mL (~21 µM). In parallel, 400 µL of 500 µM peptide **1'-Thr*** (or **1'-Hnv***) in water were incubated with 250 µM tris(2-carboxyethyl)phosphine hydrochloride (TCEP) for 1 h at rt to fully reduce any possible symmetric disulfide. For the peptide coupling reaction 325 µL of freshly TCEP-treated peptide **1'-Thr*** (or **1'-Hnv***) were added to the freshly prepared cold solution of linker-functionalized BSA (480 µL, 21 µM) and shaken at 500

rpm overnight at 4 ºC. The typical reaction volume was 1500 µL and the final concentrations in the reaction mixture were: linker-functionalized BSA ~6.7 µM, 30 mM phosphate buffer pH 7.0, 108 µM peptide. Excess uncoupled peptide was removed by ultrafiltration on Amicon Ultra-4 Centrifugal Filter Units, regenerated cellulose – 30 kDa (3 × 4 mL PBS). Purified **BSA-1'-Thr*** (or **BSA-1'-Hnv***) was taken up in 500 µL PBS at a concentration of 1.2 mg/mL (~18 µM). The sample was stored protected from light at +4° C and used to coat the ELISA plates for the determination of antigen-specific antibody titers. Successful conjugation of peptides to BSA was confirmed by SDS-PAGE (precast 4-20% gradient gel, staining with Coomassie Brilliant Blue (Figure S13c).

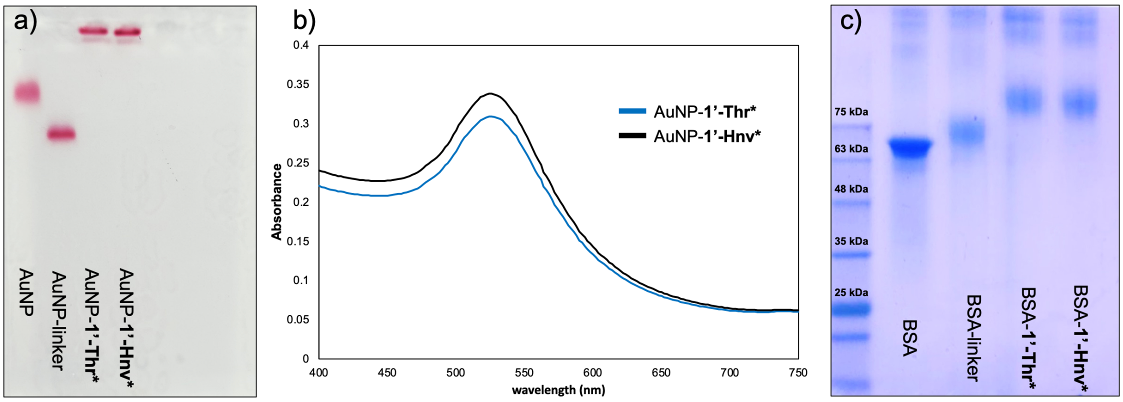

Fig. S13. (a) Agarose gel electrophoresis of passivated AuNP, AuNP-linker (loaded with the SM(PEG)2-linker), AuNP-**1'-Thr*** and AuNP-**1'-Hnv***. (b) UV-Vis spectra of two representative AuNPs batches. λ_{max}= 526 nm was used. (c) SDS-PAGE gel electrophoresis showing the successful conjugation of glycopeptides **1-Thr*** and **1'-Hnv*** to BSA through the SM(EG)2 amine-to-sulfhydryl heterobifunctional linker. Precast 4-20% gradient gel stained with Coomassie Brilliant Blue.

11. Determination of the apparent K_D of AuNP-1'-Thr* and AuNP-1'-Hnv* and the antibody SM3.

To investigate the binding affinity between the antigens immobilized on PEGylated AuNPs and mouse monoclonal anti-MUC1 antibody [SM3] (abcam ab22711), the apparent dissociation constants (K_D) of each peptide was determined using a dot-blot assay with fluorescence detection (Fig. S14).

Briefly, each conjugate (AuNP-**1'-Thr* or** AuNP-**1'-Hnv*)** was spotted (3µL, 4nM) in quintuplicate onto methanol-activated PVDF membranes, which were allowed to dry at rt. Nine identical PVDF membranes were prepared (Fig. S14a). Each membrane was rinsed with Tris-buffered saline, blocked with 5% non-fat milk, and incubated separately

overnight at 4°C with anti-MUC1 antibody (2mL) at different concentrations in 5% non-fat milk (three-fold serial dilution starting at 1.0 µg/mL, i.e. 3.33×10-1 µg/mL, 1.11×10-1 µg/mL, 3.70×10-2 µg/mL, 1.23×10-2 µg/mL, 4.11×10-3 µg/mL, 1.37×10-3 µg/mL, 4.57×10-4 µg/mL, 1.52×10-4 µg/mL). A goat anti-mouse IgG-Atto 633 fluorescent secondary antibody was used then for detection (Fig. S14b).

The K_D values were obtained using a nonlinear curve fitting of the fluorescence intensity values measured at each of the 9-antibody concentration averaged for the 5 replicates. The binding curves were fit to a one site binding equation:

$$y = \frac{B_{max}\, x}{K_D + x} \quad \text{(Equation 1)}$$

This experiment was performed twice, with the following result (these values were obtained assuming the molar mass of the SM3 antibody equal to 150 kDa. Fig. S14c):

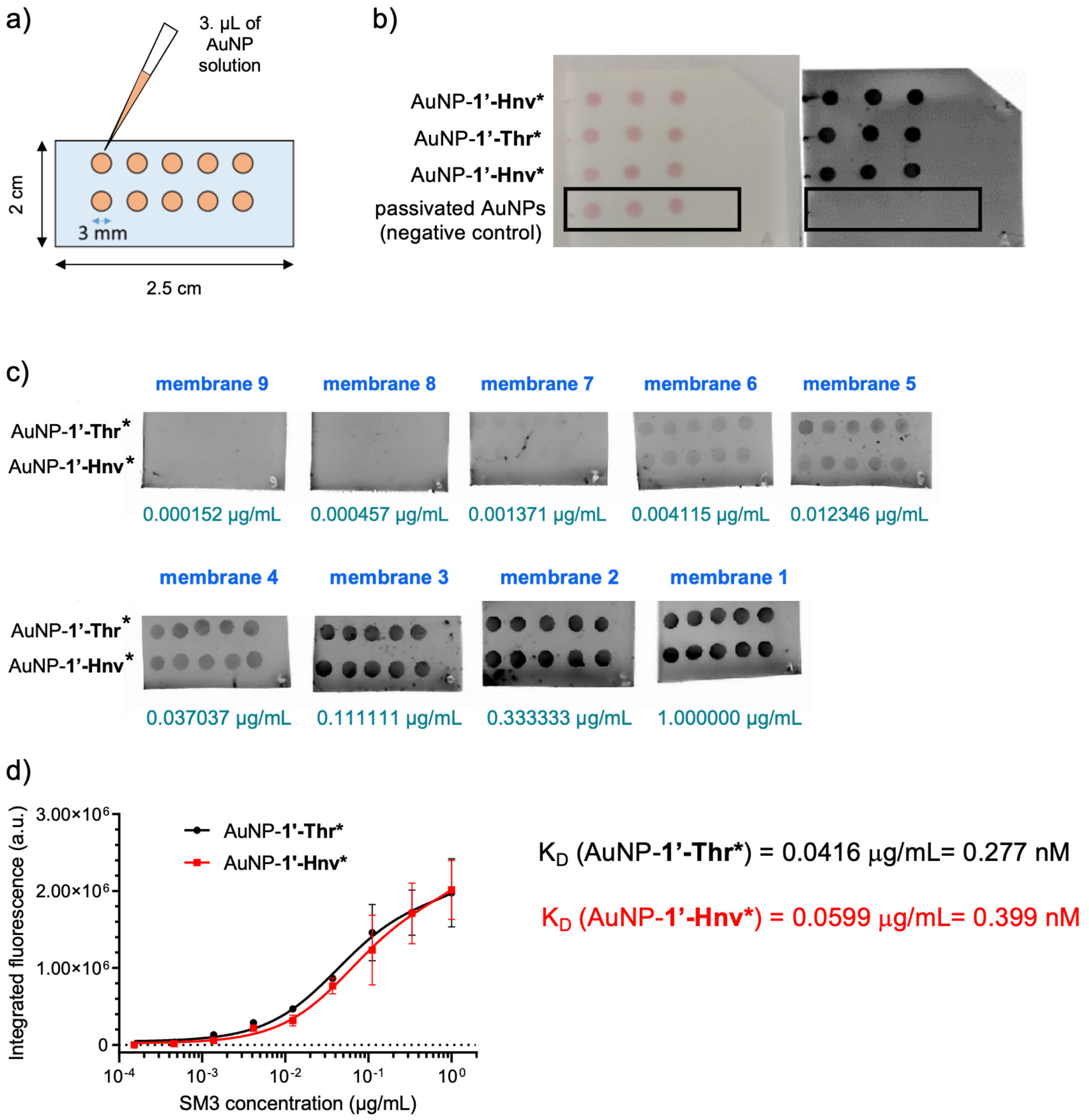

Fig. S14. (a) Schematic representation of the experiments performed on methanol-activated PVDF membranes. (b) Images of a PVDF membrane spotted with AuNP-**1'-Hnv***, AuNP-**1'-Thr***, and passivated AuNPs (used as a negative control) incubated with 0.01 µg/mL of SM3 antibody. Left: Image in the visible showing the red colored spots corresponding to the deposited AuNPs. Right: fluorescence images showing the fluorescence coming from the secondary antibody. (c) Images of the scanned PVDF membranes of the assay of AuNP-**1'-Thr*** and AuNP-**1'-Hnv*** using an increasing concentration of the SM3 antibody. (d) Binding curves and estimation of the K_D values for the two conjugates. These K_D values were obtained assuming the molar mass of the SM3 antibody equal to 150 kDa.

12. *In vivo* studies

12.1. Animals and immunization protocol. Animal experiments were conducted at the Instituto de Medicina Molecular João Lobo Antunes (iMMLisboa-JLA, Portugal). Animal work was performed in strict accordance with the Portuguese Law (Portaria 1005/92) and the European Guideline 86/609/EEC and follow the FELASA (Federation of European Laboratory Animal Science Associations) guidelines and recommendations concerning laboratory animal welfare. Furthermore, all animal experiments were approved by the Portuguese DGAV S39 and the IMM Animal Ethics Committee (authorization AEC_2014_07_GB_Vaccines). The vaccine candidate was administrated to 8-week-old Balb/c mice purchased from Charles River (Spain).

Mice were randomly divided into 3 experimental groups: **1-Thr*** peptide control (n=5), AuNP-**1'-Thr*** (n=5), and AuNP-**1'-Hnv*** (n=5). Animals were vaccinated receiving the selected vaccine candidate formulated in PBS buffer via intraperitoneal injection (50 μL corresponding to 3 μg of peptide in all cases). The prime dose was administered to 8-weeks old animals followed by three additional booster doses at 21 days intervals. Five days after the last booster, mice were sacrificed, and whole blood was collected by heart puncture to obtain blood serum. We decided to use the nanoparticles without additional adjuvant to investigate the intrinsic adjuvanticity of the nanoparticle-based formulation.

12.2. Antibody titer determination by Enzyme-Linked Immunosorbent Assay (ELISA). Total IgG antibody titers generated by the vaccine candidates were determined by ELISA, as reported,[22] with minor changes (Fig. 6b and S15). Briefly, 96 well Maxisorb ELISA plates (Nunc) were coated with BSA-**1'-Thr*** conjugate in PBS at 4 °C overnight (30 μL/well, 10 μg/mL in coating buffer: 0.1 M Na_2HPO_4, pH 9.4). The coated plates were washed 3 times with PBS and blocked with 1% BSA in PBS, 50 μL/well, overnight at 4 °C. Another washing step was performed before the incubation with the mouse sera. Serial dilutions of the sera (1:2, 11 serial dilutions in PBS, starting from 1:100, 50 μL/well) were added to the plates and incubated for 1 h at rt. After 3 washing steps, the plates were incubated with the secondary antibody Goat anti-mouse IgG HRP (Invitrogen, 1:3000 dilution in blocking buffer, 50 μL/well) for 1 h at rt. Three additional washing steps were performed. The assays were developed with 90 μL/well TMB 1x solution (eBioscience) for 10 min at RT. The TMB kinetic reaction was stopped with ELISA Stop Solution which resulted in a color change from blue to yellow that was possible to read at 450 nm by using an Infinite M200 plate reader. Background absorbance values (absorbance obtained for wells incubated only with the secondary antibody - blank) were subtracted before.

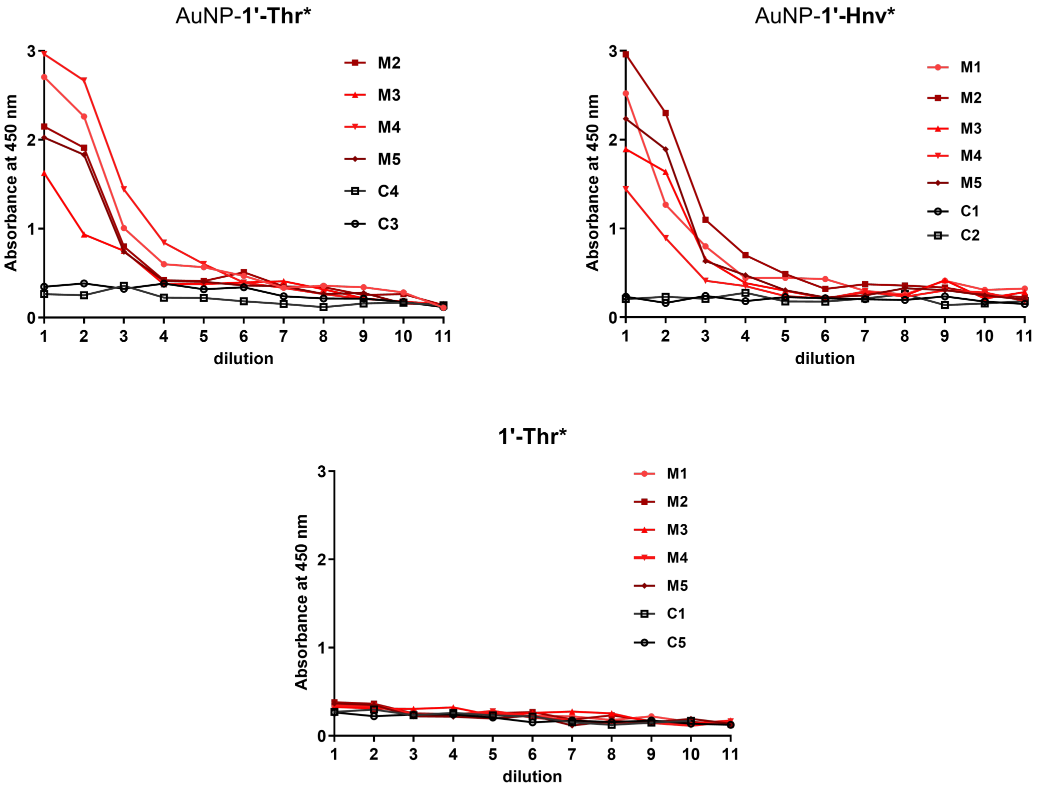

Fig. S15. Total antibody titers (IgG) after immunization with **AuNP-1'-Thr***, **AuNP-1'-Thr*** or **1'-Thr** determined by ELISA. The glycopeptide **1'-Thr*** was used as a negative control. The microtiter plate surface was coated with BSA-**1'-Hnrv***. 1-11 numbers correspond to serial 1:2 (or twofold) dilutions starting at 1:100.

12.3. Antibody isotype determination by ELISA. The ELISA protocol performed to evaluate the antibody isotypes in the sera was the same as described above. Briefly, the plates were coated with BSA-**1'-Thr*** or BSA-**1'-Hnv*** conjugates (Fig. 6b and S16) at 4 ºC overnight, blocked with ELISA blocking buffer (1 h at rt), followed by incubation with 1:200 dilutions of the end-point sera (1 h at rt). The plates were then incubated with anti-mouse isotype antibody IgG1-HRP (1:1000, eBioscience), IgG2a-HRP (1:50.000, Abcam), IgG2b-HRP (1:50.000, Abcam), IgG3-HRP (1:50.000, Abcam) and IgM-HRP (1:50.000, Abcam) for 1 h at rt. The TMB kinetic reaction was further performed by adding 90 µL of TMB 1x solution to the plates. After 5 min. at rt, the reaction was stopped with ELISA stop solution (1 M H_2SO_4) and read at 450 nm with an Infinite M200 plate reader (Fig. S16).

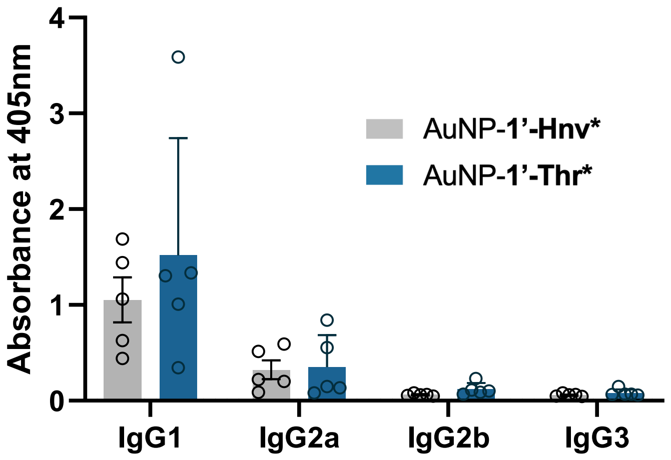

Fig. S16. Total and subtyping (IgG1, IgG2a/b and IgG3) anti-MUC1 antibodies after immunizing mice (n = 5 per experimental group) with AuNP-**1'-Thr** and AuNP-**1'-Hnv** vaccine candidates (dilution 1/200). ELISA plates were coated with BSA-**1'-Thr*** conjugate. Bars represent the mean +/- SD of all animals. No significant difference in antibody subtypes was observed between the two vaccine candidates.

12.4. Antibody reactivity towards MUC1 positive cancer cells analyzed by confocal microscopy. Antisera staining of cell lines T47D and HEK293T were investigated by confocal microscopy. To this end, 30.000 cells/well were seeded in 8- well µ-Slide Ibidi Plates and grown for 24 h. Cells were then fixed with 4% paraformaldehyde in PBS for 10 min at 37 ºC, washed with PBS and permeabilized with 1% triton X-100 in PBS for 15 min at rt. The cells were then incubated with the mice sera (1:100 dilution) at 4 ºC overnight. On the following day, cells were washed with PBS and incubated with 200 µL/well of goat anti-mouse polyclonal IgG H&L Alexa Fluor 488 (1:2000) secondary antibody from Abcam, for 2 h at rt. Finally, cells were washed and incubated with Hoechst (1 µg/mL) for 10 min at rt to stain nuclei and analyzed using a Zeiss LSM 710 confocal Laser Point-Scanning Microscope with a 40X oil objective and numerical aperture =1.3. The secondary antibody was visualized using an argon laser source (488 nm, emission 500-550 nm) and nuclei were visualized using a diode 405-30 laser (450nm, emission 420-470 nm).

NMR spectra

^{1}H NMR (400 MHz) in $CDCl_3$ registered at 298K for compound 3

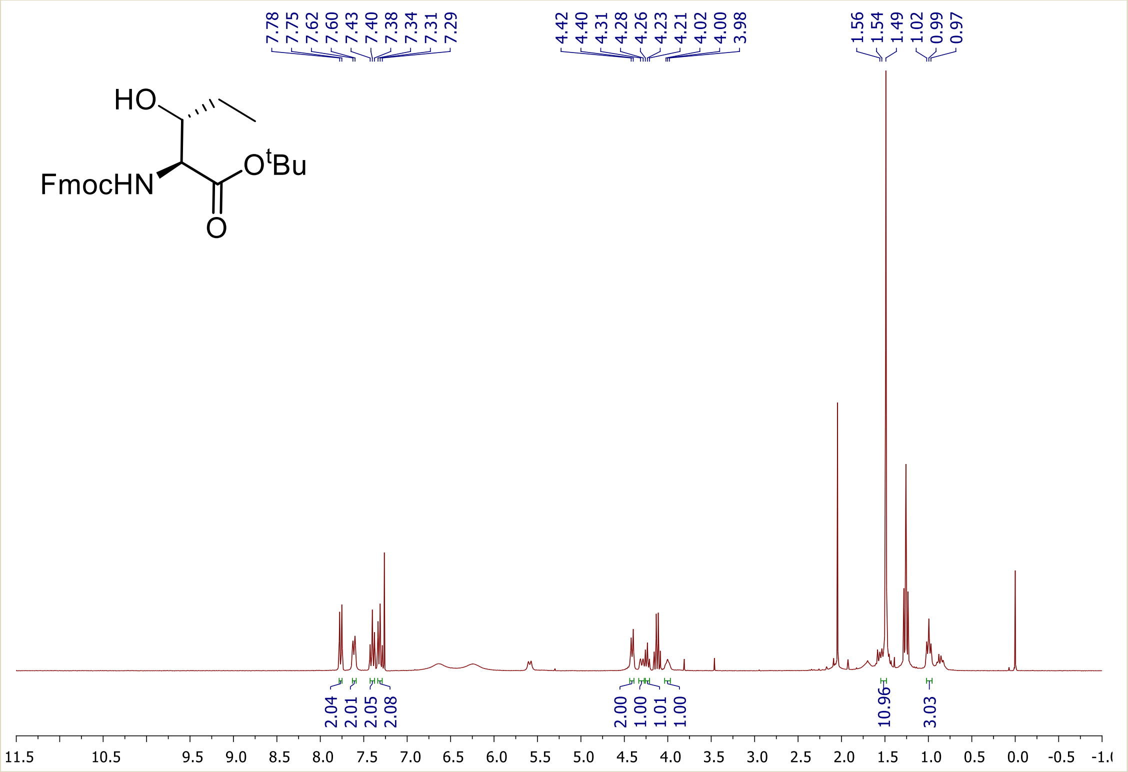

^{13}C NMR (75 MHz) in $CDCl_3$ registered at 298K for compound 3

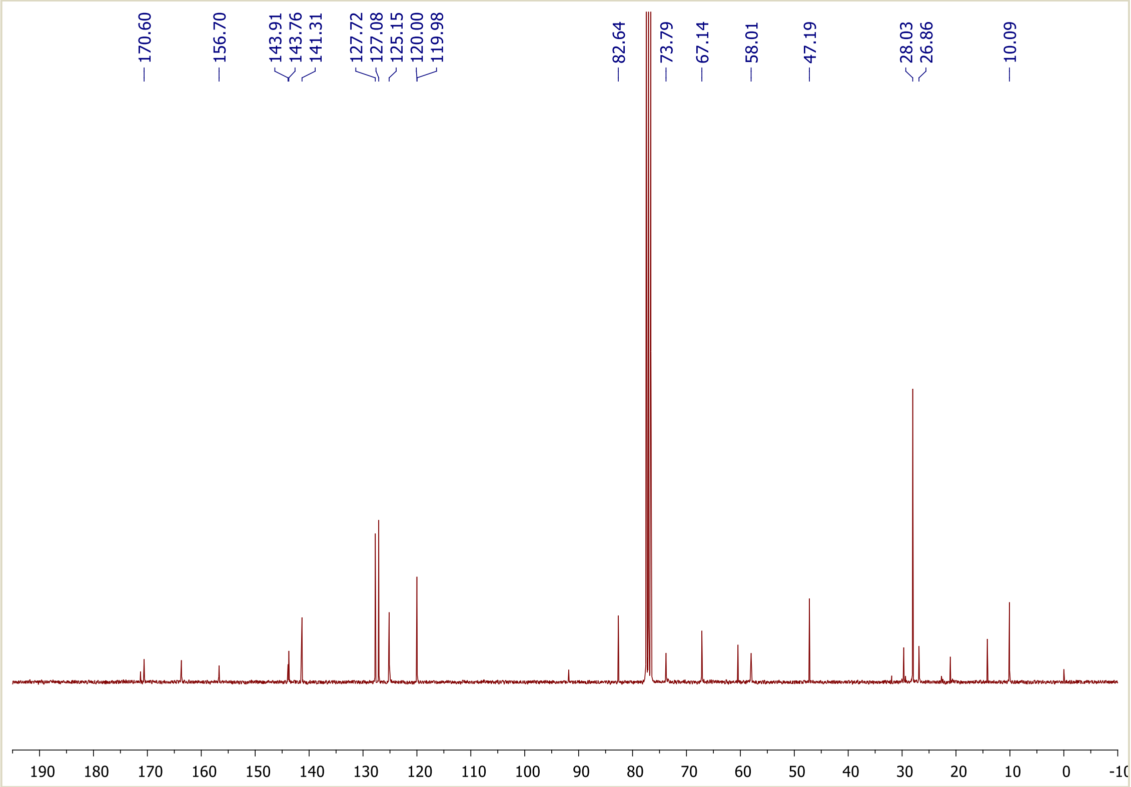

^{1}H NMR (400 MHz) in $CDCl_3$ registered at 298K for compound 6

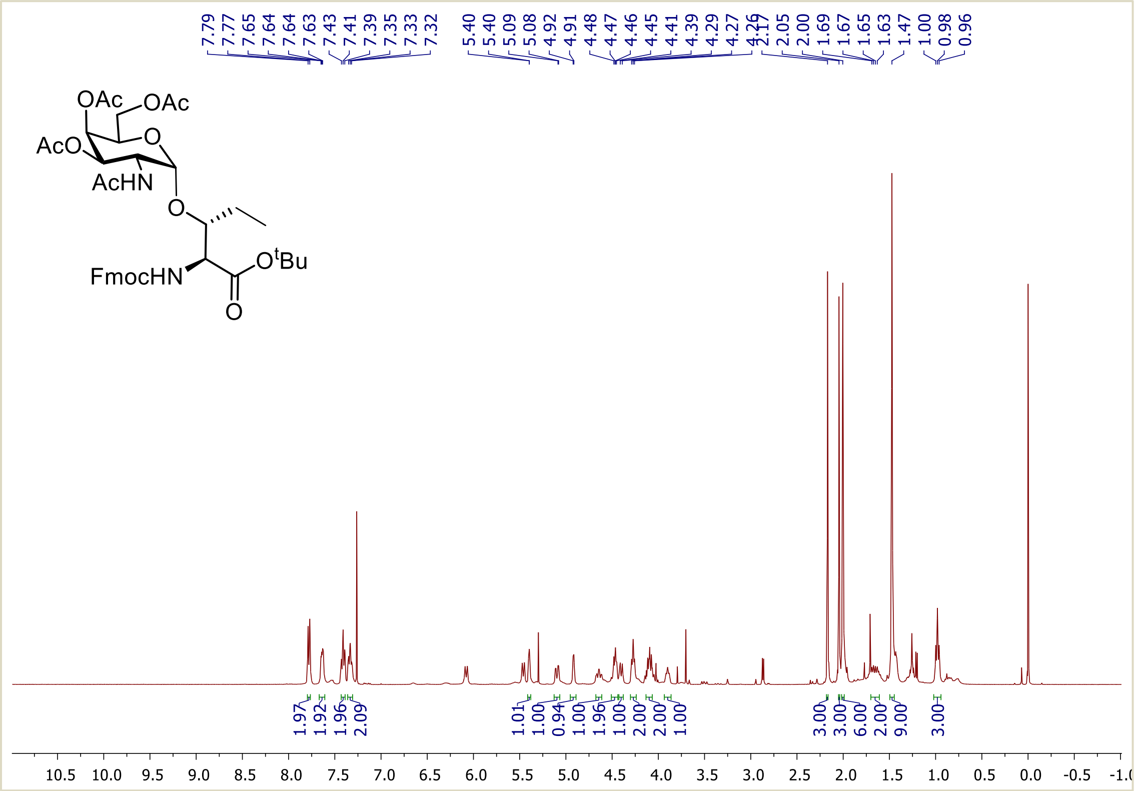

^{13}C NMR (75 MHz) in $CDCl_3$ registered at 298K for compound 6

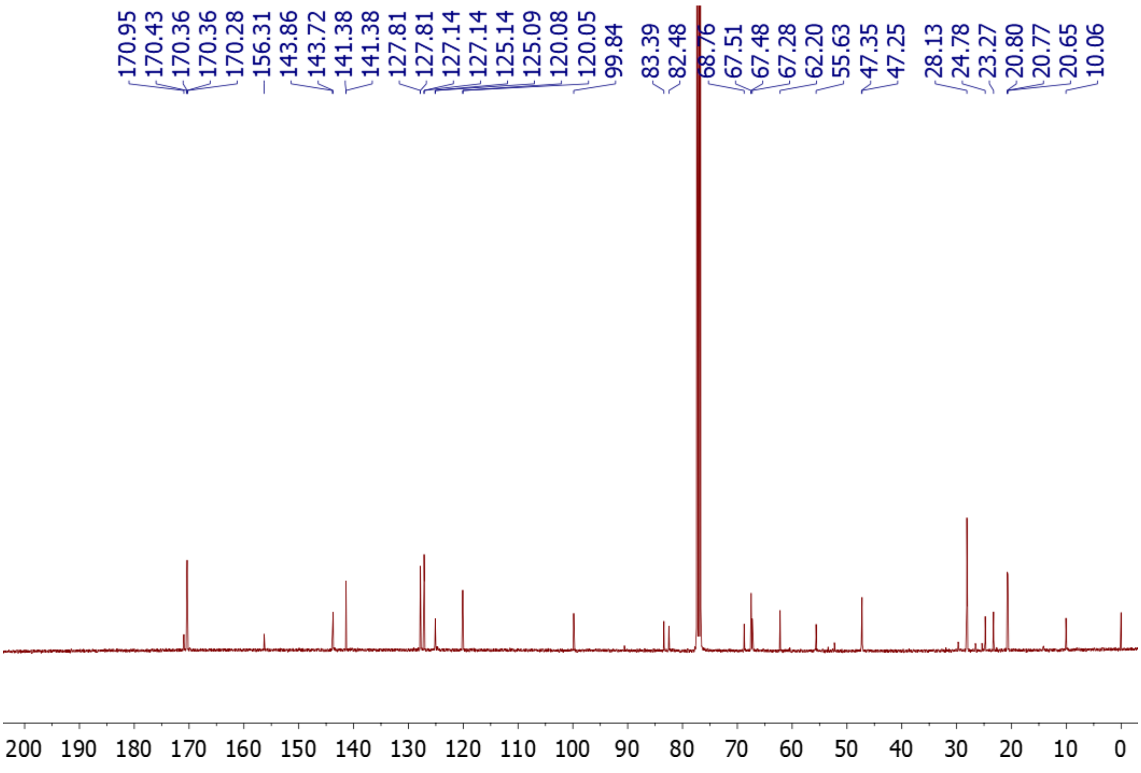

^{1}H NMR (400 MHz) in CDCl$_3$ registered at 298K for compound 10

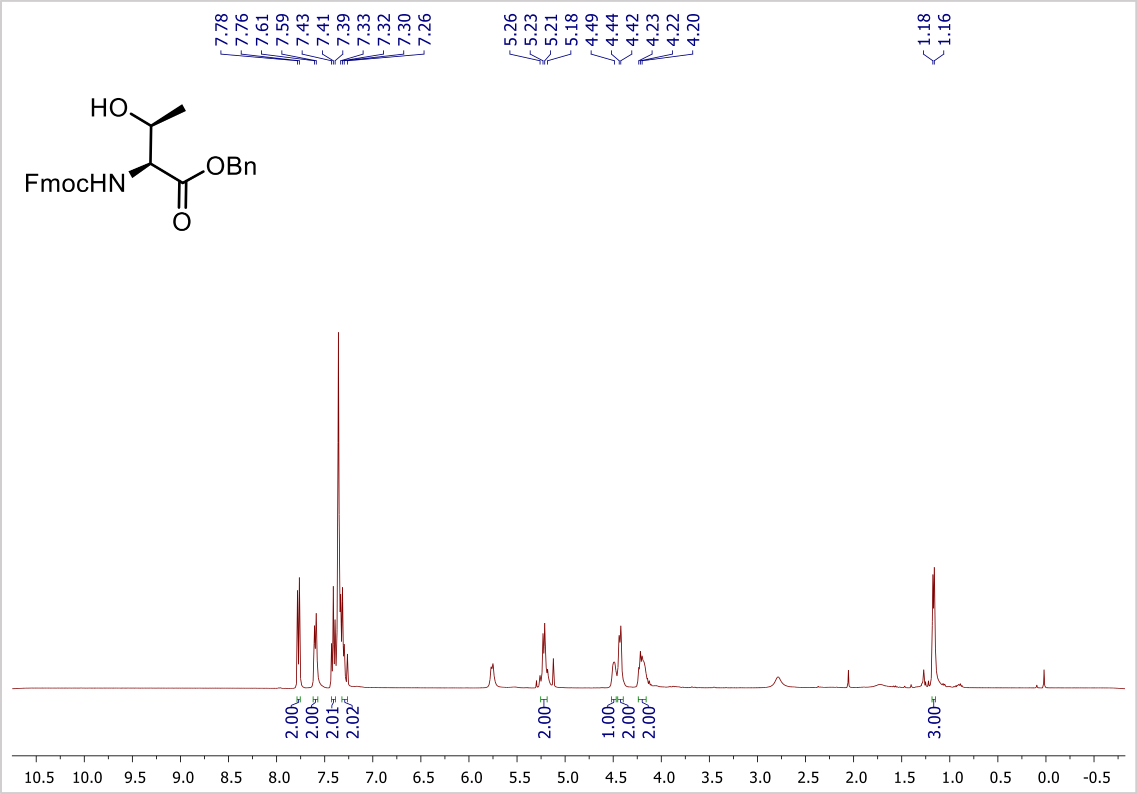

^{13}C NMR (75 MHz) in CDCl$_3$ registered at 298K for compound 10

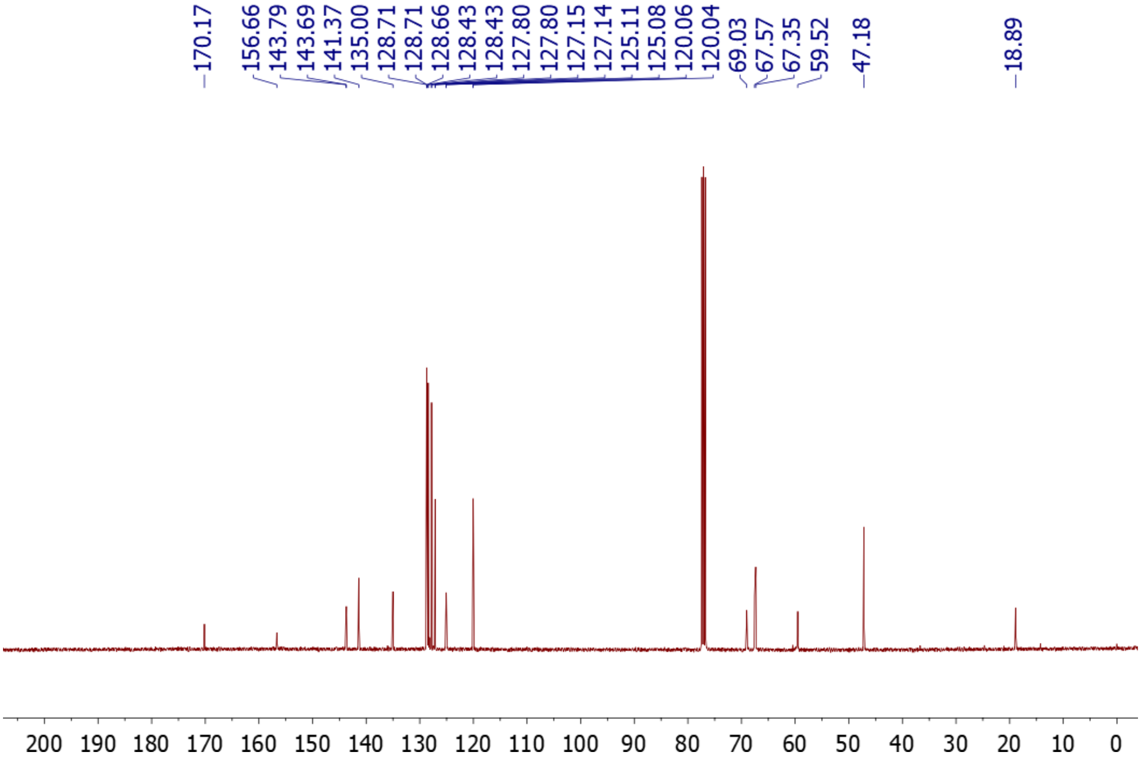

^{1}H NMR (400 MHz) in $CDCl_3$ registered at 298K for compound 12

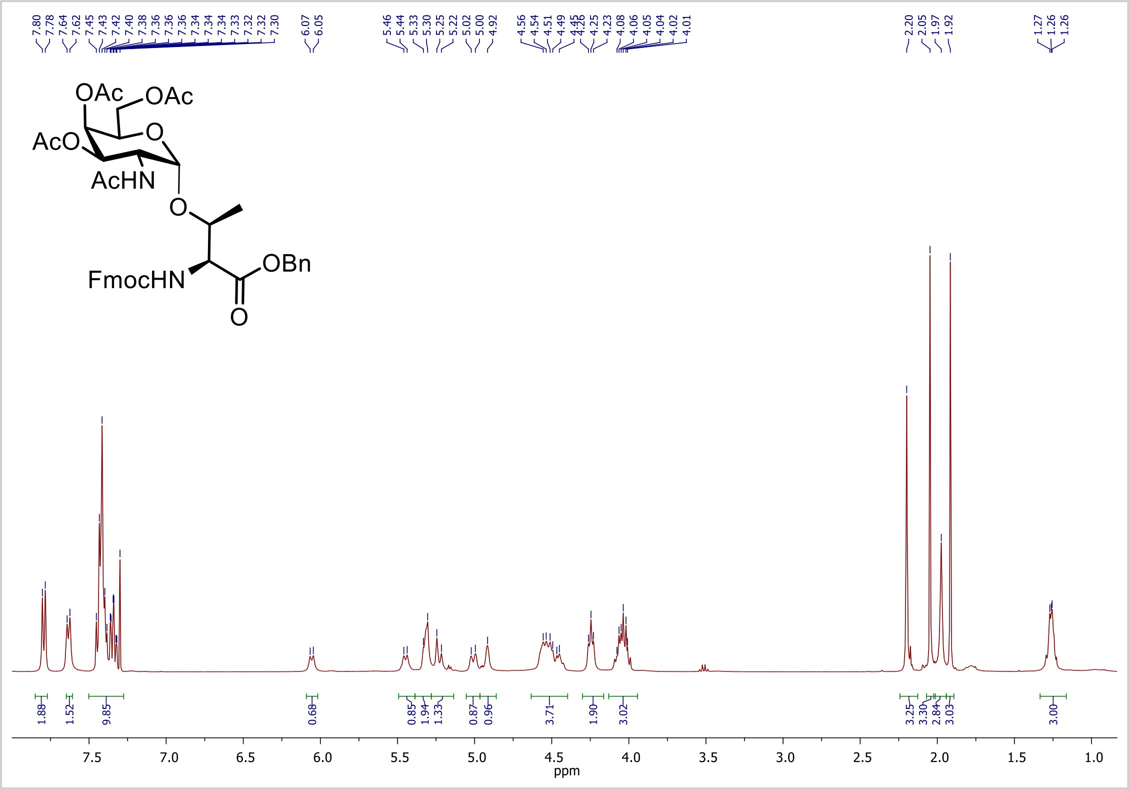

^{13}C NMR (75 MHz) in $CDCl_3$ registered at 298K for compound 12

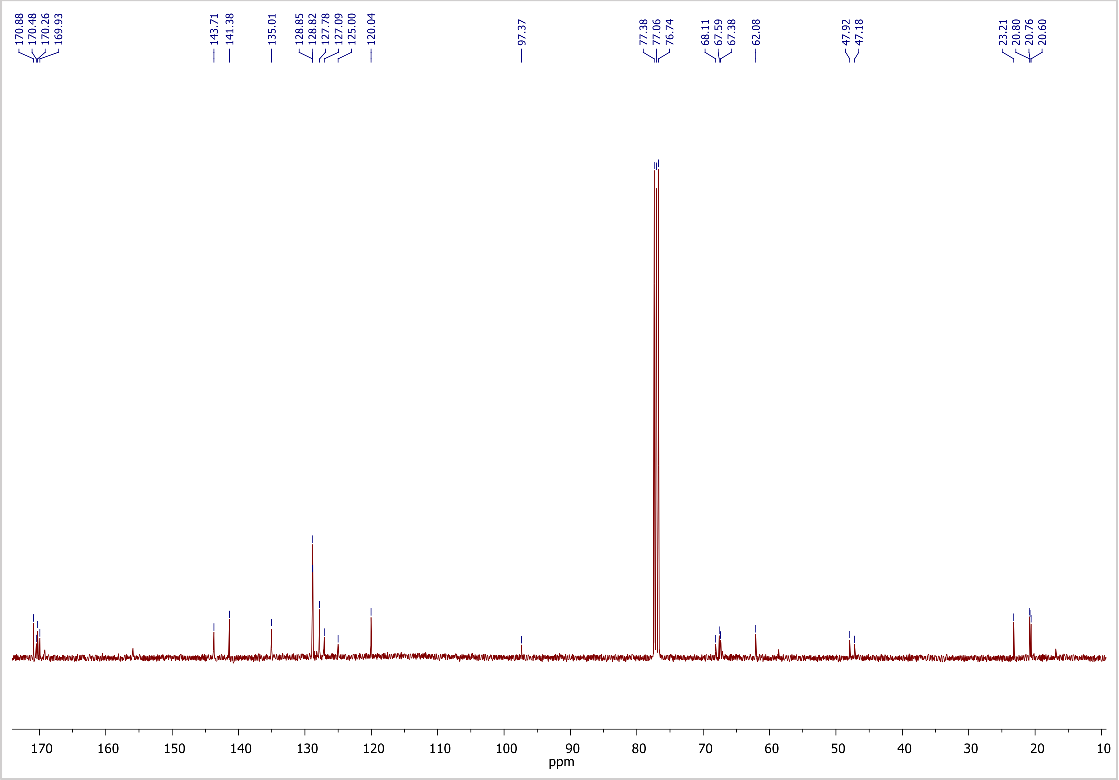

^{1}H NMR (400 MHz) in D_2O registered at 298K for glycopeptide 2-aThr*

Note: A second set of signals (small relative intensity) is observed. They correspond to the cis configuration of the amide bond of proline residues.[5]

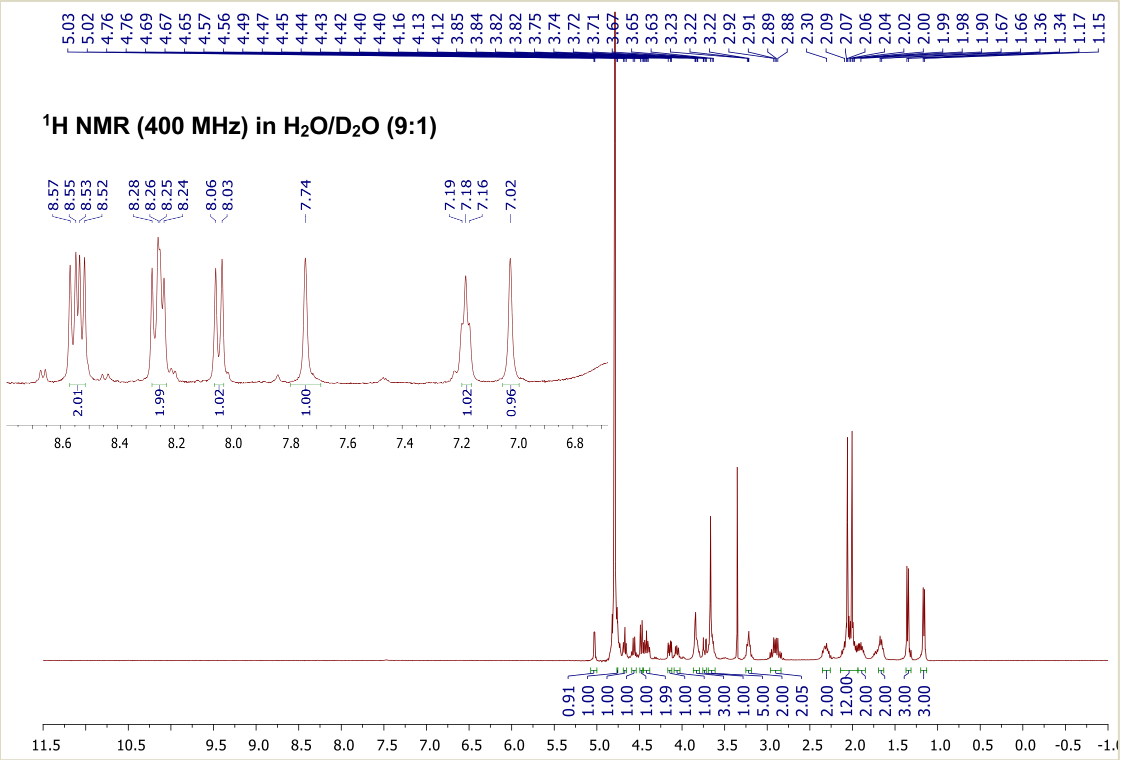

^{13}C NMR (75 MHz) in D_2O registered at 298K for glycopeptide 2-aThr*

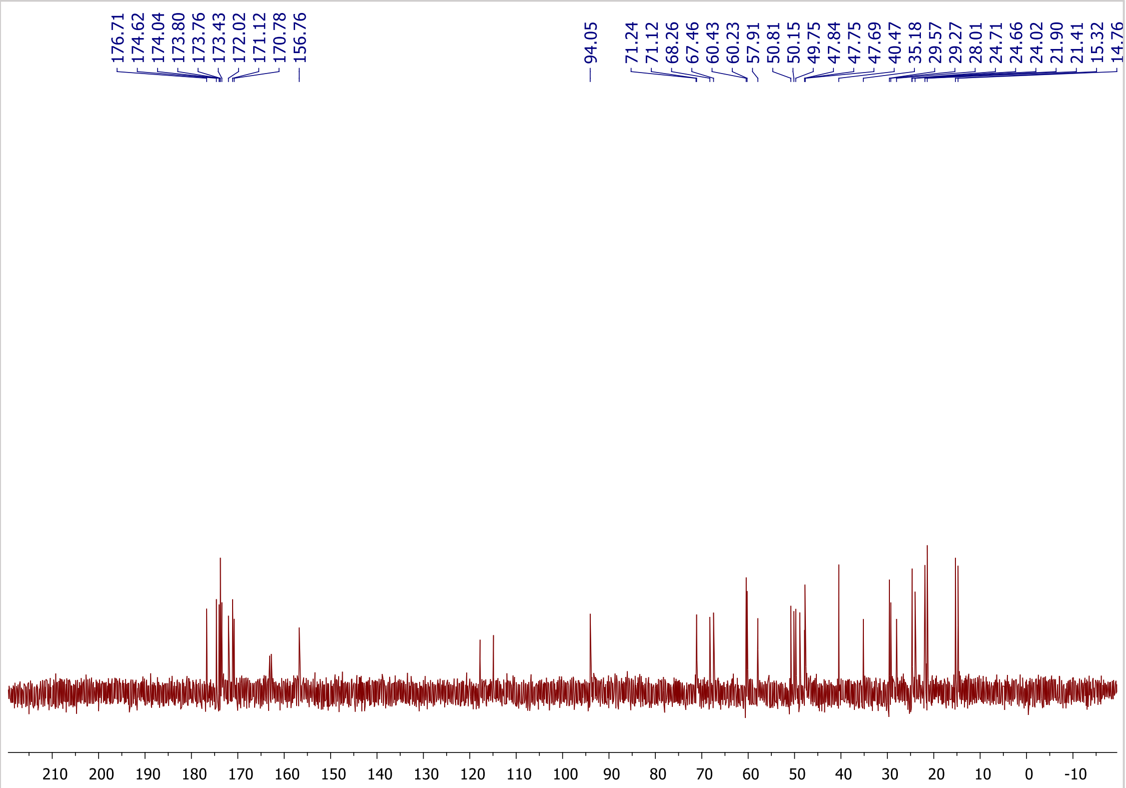

ROESY NMR (400 MHz) in H_2O/D_2O (9:1) registered at 298K for glycopeptide 2-aThr*

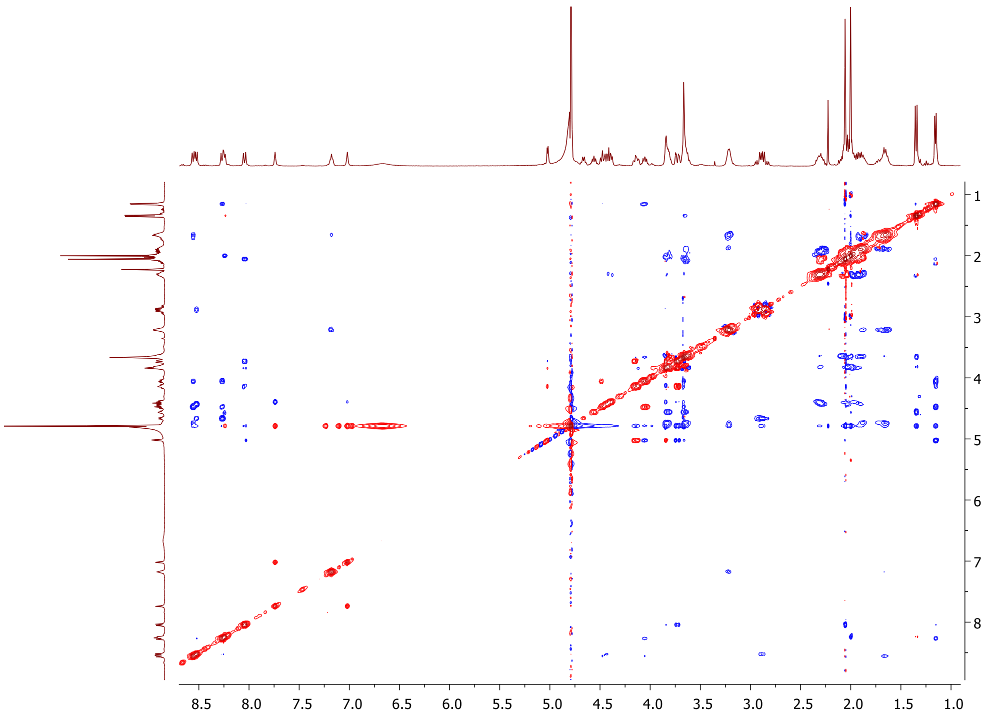

^{1}H NMR (400 MHz) in D_2O registered at 298K for glycopeptide 2-Hnv*

Note: A second set of signals (small relative intensity) is observed. They correspond to the cis configuration of the amide bond of proline residues.[5]

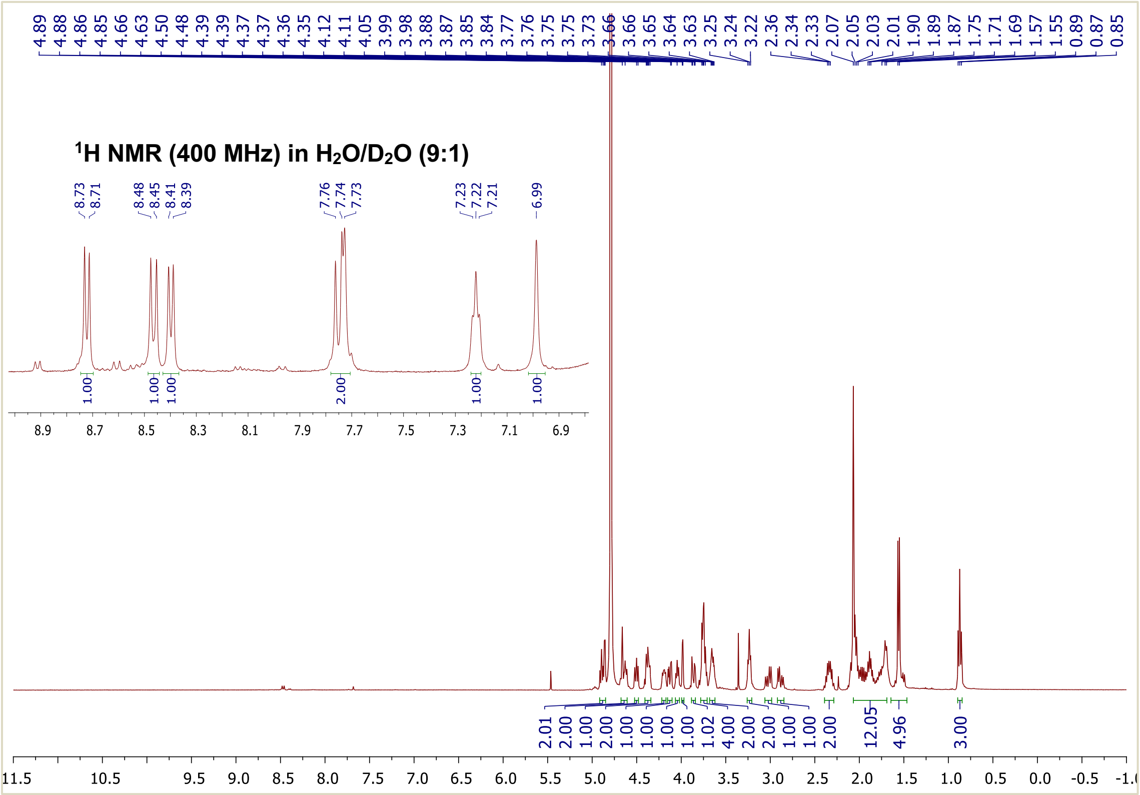

^{13}C NMR (75 MHz) in D_2O registered at 298K for glycopeptide 2-Hnv*

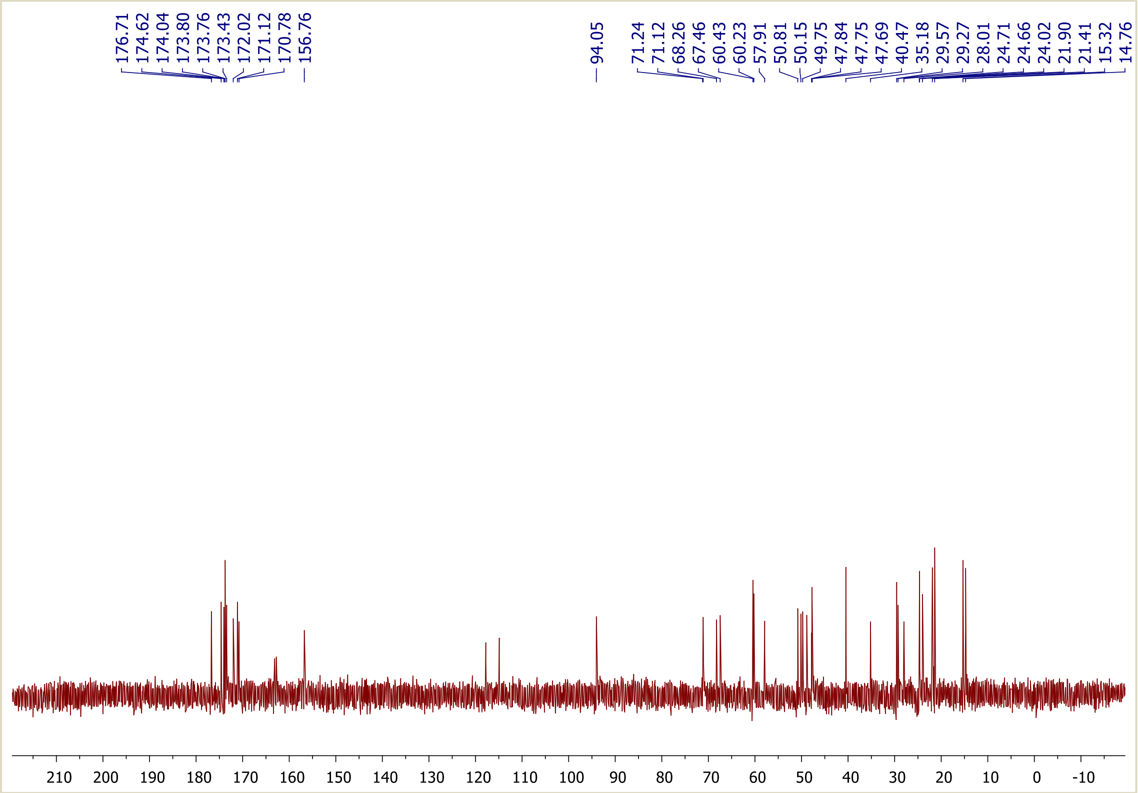

ROESY NMR (400 MHz) in H_2O/D_2O (9:1) registered at 298K for glycopeptide 2-Hnv*

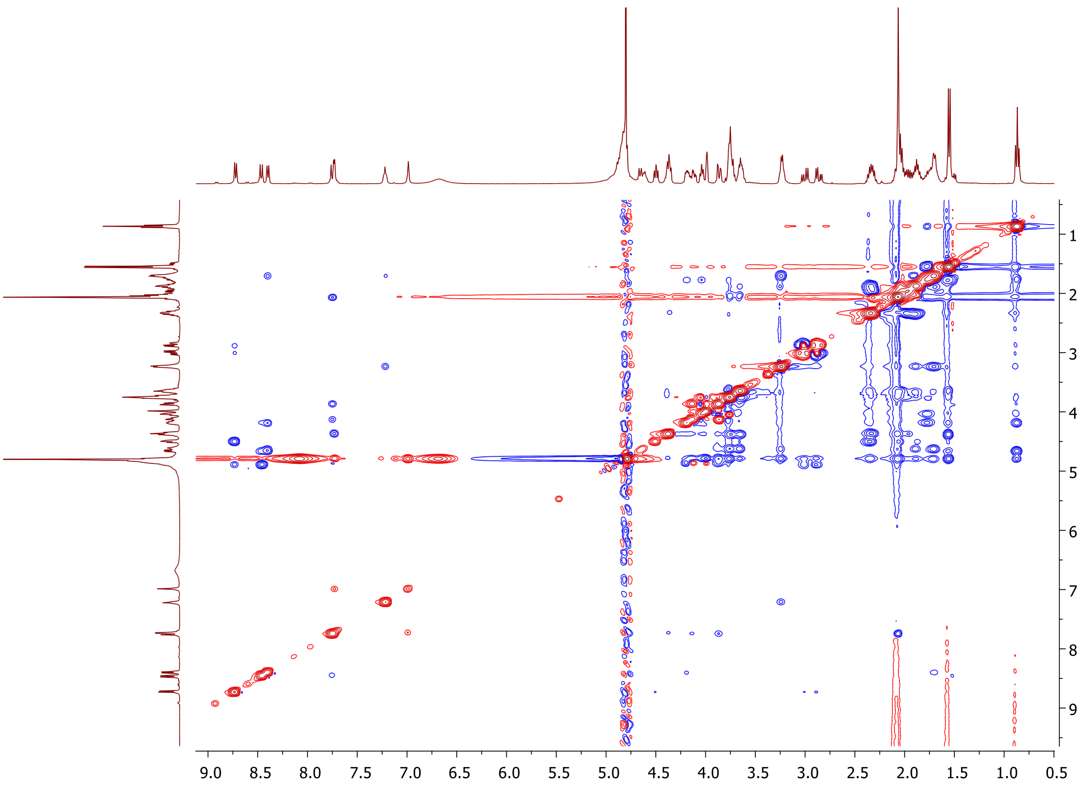

References

(1) Plattner, C.; Höfener, M.; Sewald, N. One-Pot Azidochlorination of Glycals. *Org. Lett.* **2011**, *13*, 545-547.

(2) Martinez-Saez, N.; Castro-Lopez, J.; Valero-Gonzalez, J.; Madariaga, D.; Companon, I.; Somovilla, V. J.; Salvado, M.; Asensio, J. L.; Jimenez-Barbero, J.; Avenoza, A.; et al. Deciphering the Non-Equivalence of Serine and Threonine O-Glycosylation Points: Implications for Molecular Recognition of the Tn Antigen by an anti-MUC1 Antibody. *Angew. Chem. Int. Ed.* **2015**, *54*, 9830-9834.

(3) Karsten, U. Binding patterns of DTR-specific antibodies reveal a glycosylation-conditioned tumor-specific epitope of the epithelial mucin (MUC1). *Glycobiology* **2004**, *14*, 681-692.

(4) Coelho, H.; Matsushita, T.; Artigas, G.; Hinou, H.; Cañada, F. J.; Lo-Man, R.; Leclerc, C.; Cabrita, E. J.; Jiménez-Barbero, J.; Nishimura, S.-I.; et al. The Quest for Anticancer Vaccines: Deciphering the Fine-Epitope Specificity of Cancer-Related Monoclonal Antibodies by Combining Microarray Screening and Saturation Transfer Difference NMR. *J. Am. Chem. Soc.* **2015**, *137*, 12438-12441.

(5) Dziadek, S.; Griesinger, C.; Kunz, H.; Reinscheid, U. M. Synthesis and Structural Model of an α(2,6)-Sialyl-T Glycosylated MUC1 Eicosapeptide under Physiological Conditions. *Chem. Eur. J.* **2006**, *12*, 4981-4993.

(6) Martinez-Saez, N.; Supekar, N. T.; Wolfert, M. A.; Bermejo, I. A.; Hurtado-Guerrero, R.; Asensio, J. L.; Jimenez-Barbero, J.; Busto, J. H.; Avenoza, A.; Boons, G. J.; et al. Mucin architecture behind the immune response: design, evaluation and conformational analysis of an antitumor vaccine derived from an unnatural MUC1 fragment. *Chem. Sci.* **2016**, *7*, 2294-2301.

(7) Madariaga, D.; Martínez-Sáez, N.; Somovilla, V. J.; Coelho, H.; Valero-González, J.; Castro-López, J.; Asensio, J. L.; Jiménez-Barbero, J.; Busto, J. H.; Avenoza, A.; et al. Detection of Tumor-Associated Glycopeptides by Lectins: The Peptide Context Modulates Carbohydrate Recognition. *ACS Chem. Biol.* **2015**, *10*, 747-756.

(8) Case, D. A. B.-S., I.Y.; Brozell, S.R.; Cerutti, D.S.; Cheatham, T.E.; III, Cruzeiro, V.W.D.; Darden, T.A.; Duke, R.E.; Ghoreishi, D.; Gilson, M.K.; Gohlke, H.; Goetz, A.W.; Greene, D.; Harris, R.; Homeyer, N.; Izadi, S.; Kovalenko, A.; Kurtzman, T.; Lee, T.S.; LeGrand, S.; Li, P.; Lin, C.; Liu, J.; Luchko, T.; Luo, R.; Mermelstein, D.J.; Merz, K.M.; Miao, Y.; Monard, G.; Nguyen, C.; Nguyen, H.; Omelyan, I.; Onufriev, A.; Pan, F.; Qi, R.; Roe, D.R.; Roitberg, A.; Sagui, C.; Schott-Verdugo, S.; Shen, J.; Simmerling, C.L.; Smith, J.; Salomon-Ferrer, R.; Swails, J.; Walker, R.C.; Wang, J.; Wei, H.; Wolf, R.M.; Wu, X.; Xiao, L.; York, D.M.; Kollman P.A. (2018), AMBER 2018, University of California, San Francisco.

(9) Maier, J. A.; Martinez, C.; Kasavajhala, K.; Wickstrom, L.; Hauser, K. E.; Simmerling, C. ff14SB: Improving the Accuracy of Protein Side Chain and Backbone Parameters from ff99SB. *J. Chem. Theory Comput.* **2015**, *11*, 3696-3713.

(10) Wang, J.; Wolf, R. M.; Caldwell, J. W.; Kollman, P. A.; Case, D. A. Development and testing of a general amber force field. *J. Comput. Chem.* **2004**, *25*, 1157-1174.

(11) Kirschner, K. N.; Yongye, A. B.; Tschampel, S. M.; González-Outeiriño, J.; Daniels, C. R.; Foley, B. L.; Woods, R. J. GLYCAM06: a generalizable biomolecular force field. Carbohydrates. *J. Comput. Chem.* **2008**, *29*, 622-655.

(12) Jakalian, A.; Jack, D. B.; Bayly, C. I. Fast, efficient generation of high-quality atomic charges. AM1-BCC model: II. Parameterization and validation. *J. Comput. Chem.* **2002**, *23*, 1623-1641.

(13) Jorgensen, W. L.; Chandrasekhar, J.; Madura, J. D.; Impey, R. W.; Klein, M. L. Comparison of simple potential functions for simulating liquid water. *J. Chem. Phys.* **1983**, *79*, 926-935.

(14) Darden, T.; York, D.; Pedersen, L. Particle mesh Ewald: An N·log(N) method for Ewald sums in large systems. *J. Chem. Phys.* **1993**, *98*, 10089-10092.

(15) Stenutz, R.; Carmichael, I.; Widmalm, G.; Serianni, A. S. Hydroxymethyl Group Conformation in Saccharides: Structural Dependencies of 2JHH, 3JHH, and 1JCH Spin−Spin Coupling Constants. *J. Org. Chem.* **2002**, *67*, 949-958.

(16) Kabsch, W. XDS. *Acta Crystallogr. D Biol. Crystallogr.* **2010**, *66* (Pt 2), 125-132.

(17) Winn, M. D.; Ballard, C. C.; Cowtan, K. D.; Dodson, E. J.; Emsley, P.; Evans, P. R.; Keegan, R. M.; Krissinel, E. B.; Leslie, A. G.; McCoy, A.; et al. Overview of the CCP4 suite and current developments. *Acta Crystallogr. D Biol. Crystallogr.* **2011**, *67*, 235-242.

(18) Emsley, P.; Cowtan, K. Coot: model-building tools for molecular graphics. *Acta Crystallogr. D Biol. Crystallogr.* **2004**, *60*, 2126-2132.

(19) Murshudov, G. N.; Skubák, P.; Lebedev, A. A.; Pannu, N. S.; Steiner, R. A.; Nicholls, R. A.; Winn, M. D.; Long, F.; Vagin, A. A. REFMAC5 for the refinement of macromolecular crystal structures. *Acta Crystallogr. D Biol. Crystallogr.* **2011**, *67*, 355-367.

(20) Laskowski, R. A.; MacArthur, M. W.; Moss, D. S.; Thornton, J. M. PROCHECK: a program to check the stereochemical quality of protein structures. *J. Appl. Cryst.* **1993**, *26*, 283-291.

(21) Dokurno, P.; Bates, P. A.; Band, H. A.; Stewart, L. M.; Lally, J. M.; Burchell, J. M.; Taylor-Papadimitriou, J.; Snary, D.; Sternberg, M. J.; Freemont, P. S. Crystal structure at 1.95 A resolution of the breast tumour-specific antibody SM3 complexed with its peptide epitope reveals novel hypervariable loop recognition. *J. Mol. Biol.* **1998**, *284*, 713-728.

(22) Companon, I.; Guerreiro, A.; Mangini, V.; Castro-Lopez, J.; Escudero-Casao, M.; Avenoza, A.; Busto, J. H.; Castillon, S.; Jimenez-Barbero, J.; Asensio, J. L.; et al. Structure-Based Design of Potent Tumor-Associated Antigens: Modulation of Peptide Presentation by Single-Atom O/S or O/Se Substitutions at the Glycosidic Linkage. *J. Am. Chem. Soc.* **2019**, *141*, 4063-4072.

(23) Maggi, V.; Bianchini, F.; Portioli, E.; Peppicelli, S.; Lulli, M.; Bani, D.; Sole, R. D.; Zanardi, F.; Sartori, A.; Fiammengo, R. Gold Nanoparticles Functionalized with RGD-Semipeptides: A Simple yet Highly Effective Targeting System for αVβ3 Integrins. *Chem. Eur. J.* **2018**, *24*, 12093-12100.
